# Ecomorphological divergence and habitat lability in the context of robust patterns of modularity in the cichlid feeding apparatus

**DOI:** 10.1186/s12862-020-01648-x

**Published:** 2020-07-31

**Authors:** Andrew J. Conith, Michael R. Kidd, Thomas D. Kocher, R. Craig Albertson

**Affiliations:** 1 Biology Department, University of Massachusetts Amherst, Amherst, MA 01003 USA; 2 Department of Biology & Chemistry, Texas A&M International University, Laredo, TX 78041 USA; 3 Department of Biology, University of Maryland, College Park, MD 20742 USA

**Keywords:** Cichlid, Morphometrics, Modularity, Integration, Morphological evolution

## Abstract

**Background:**

Adaptive radiations are characterized by extreme and/or iterative phenotypic divergence; however, such variation does not accumulate evenly across an organism. Instead, it is often partitioned into sub-units, or modules, which can differentially respond to selection. While it is recognized that changing the pattern of modularity or the strength of covariation (integration) can influence the range or rate of morphological evolution, the relationship between shape variation and covariation remains unclear. For example, it is possible that rapid phenotypic change requires concomitant changes to the underlying covariance structure. Alternatively, repeated shifts between phenotypic states may be facilitated by a conserved covariance structure. Distinguishing between these scenarios will contribute to a better understanding of the factors that shape biodiversity. Here, we explore these questions using a diverse Lake Malawi cichlid species complex, *Tropheops,* that appears to partition habitat by depth.

**Results:**

We construct a phylogeny of *Tropheops* populations and use 3D geometric morphometrics to assess the shape of four bones involved in feeding (mandible, pharyngeal jaw, maxilla, pre-maxilla) in populations that inhabit deep versus shallow habitats. We next test numerous modularity hypotheses to understand whether fish at different depths are characterized by conserved or divergent patterns of modularity. We further examine rates of morphological evolution and disparity between habitats and among modules. Finally, we raise a single *Tropheops* species in environments mimicking deep or shallow habitats to discover whether plasticity can replicate the pattern of morphology, disparity, or modularity observed in natural populations.

**Conclusions:**

Our data support the hypothesis that conserved patterns of modularity permit the evolution of divergent morphologies and may facilitate the repeated transitions between habitats. In addition, we find the lab-reared populations replicate many trends in the natural populations, which suggests that plasticity may be an important force in initiating depth transitions, priming the feeding apparatus for evolutionary change.

## Background

Characterizing the pattern and magnitude of covariation among traits has been a central theme of evolutionary biology for more than 200 years [1, 2]. However, it was not until Olson and Miller [3] that the understanding of trait covariation was formalized into a statistical framework. They suggested that both developmental and functional interactions result in the observed correlations between traits that they called morphological integration. Since Olson and Miller, much effort has gone into characterizing the patterns and strength of trait correlations at various scales – from between populations to across phyla (e.g., [4, 5]). These studies often characterize the types of correlation that exist using two interdependent terms: modularity and integration. Suites of traits that appear more correlated with each other than to other traits are termed modules. The number and identity of modules across a structure is generally referred to as the pattern of modularity, whereas the strength of correlation among traits within a module is termed integration. ‘Tinkering’ (sensu [6]) with both the pattern of modularity and the magnitude of integration provides a means to alter phenotypic variation in a way that may impact how a population can respond to selection [7, 8]. For example, an anatomical structure may be more able to respond (i.e., more evolvable) if the direction of selection aligns with the major axis of covariation [9]. In addition, by parsing an organism into discrete anatomical units, each module can become a separate target for natural selection. If regions of an organism can develop and evolve independently, then this could permit an increase in morphological diversity (i.e., disparity [10]), open up unique or unoccupied niches [11], and influence the rate of evolution [7, 12].

**Fig. 1:**
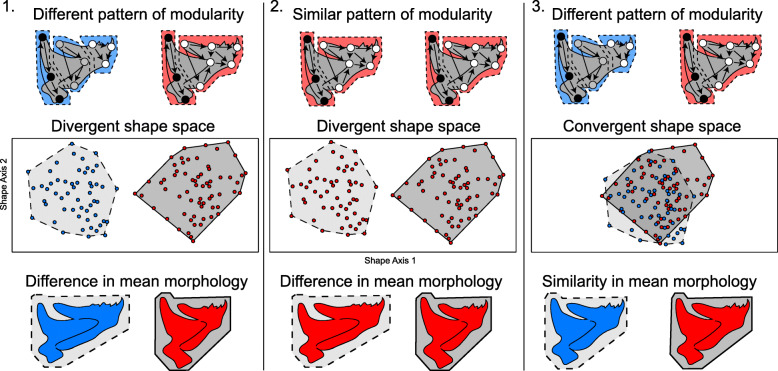
Three possible scenarios depicting the relationship between modularity and organismal morphology. Points on mandible schematics reflect example landmark coordinates, and arrows denote covariation between landmarks. Red colors reflect a pattern of covariation based on two mandibular modules, blue color reflects three mandibular modules. Numbers refer to descriptions noted in the main text

Empirical and simulation studies suggest three scenarios for potential relationships between modularity and shape (Fig. 1): 1) Differences in the pattern of modularity or magnitude of integration are associated with a corresponding change in shape. 2) A similar pattern of modularity or magnitude of integration is associated with differences in shape among populations. 3) Differences in the pattern of modularity or magnitude of integration are associated with no concomitant change in shape. Whereas scenario 1 is well supported by the literature [13–16], scenarios 2 and 3 appear to be a less common occurrence [17–19]. While all these scenarios are supported by empirical evidence, each scenario can send morphological evolution on a drastically different trajectory. As outlined above, these divergent trajectories may result in rate or disparity differences, and likely have implications for evolvability (the ability to generate adaptive phenotypic variation [20]), depending on how variation is partitioned among modules.

Cichlid fish from the East African Rift Valley provide an opportunity to examine these different scenarios of concordant or discordant changes in modularity and morphology. This lineage displays many convergent phenotypes that have evolved to exploit similar habitats and trophic niches in different lakes [17, 21]. It has been proposed that similarity in the pattern of modularity across lakes may have facilitated the iterative nature of cichlid evolution [22]. In fact, selection may act on the degree of covariation between traits, given modularity itself can evolve (reviewed by [23]), and there appears to be little overlap between loci that control shape of individual traits and those that control covariation between traits [18, 23–25]. Lake Malawi boasts the greatest taxonomic and morphological diversity of cichlids of any other African Rift Valley lake [26]. Taxa frequently partition their habitat by depth. Position along a depth gradient correlates with large differences in light, temperature, and oxygen that lead to differences in diet, predators, physiology, and sensory systems [27]. Previous studies have documented substantial morphological differences among such eco-types, especially with respect to the craniofacial skeleton [17, 22, 28]. However, there appears to be a general conservation in integration across broad taxonomic levels and feeding morphologies [29], and only minor differences in patterns of modularity [18, 30].

Ecomorphological divergence can occur via genetic mechanisms or via phenotypic plasticity. While the former may facilitate adaptation over many generations, the latter permits populations to respond to variation in environmental conditions within a generation by remodeling their morphology. Many studies have documented how populations exposed to different diets can adaptively remodel their trophic morphology to permit more efficient feeding behaviors [31–33]; however, the extent to which levels of integration or patterns of modularity can be influenced by plasticity remains an open question [but see [34] for some examples]. As Lake Malawi cichlids are known to partition habitat by depth, a feature that has led to broad differences in trophic morphology due to divergent feeding behaviors [17], phenotypic plasticity could be a means to facilitate depth transitions allowing adaptive morphological changes that could later become canalized. The mechanisms that would underlie these plastic morphological changes could center on changes to the pattern of modularity and/or the level of integration. If plasticity can facilitate a change in modularity and/or integration, this would change how variation is partitioned and accumulated in different regions of the feeding apparatus. Alternatively, if plasticity retains a particular pattern of modularity or level of integration, this may facilitate rapid morphological change along a ‘line of least resistance’ in the feeding apparatus of a population via genetic assimilation in order to adapt to a new habitat [35, 36].

Here we use the Lake Malawi cichlid genus, *Tropheops*, to more explicitly explore the relationship between morphology, modularity and evolution. *Tropheops* are one of the most rapidly evolving clades of cichlids, with speciation rates estimated to be as high as 1 per 1000 years [37]. Given this rapid diversification, we use amplified fragment length polymorphism (AFLP) to construct a phylogenetic tree of *Tropheops* populations to examine the frequency of transitions between depths and to more formally test hypotheses concerning morphological evolution. Furthermore, we focus on characterizing the feeding apparatus of *Tropheops* (mandible, lower pharyngeal jaw, maxilla, pre-maxilla), as members of this species complex occupy a spectrum of depths from shallow sediment-free conditions that require a more robust feeding apparatus to pluck attached filamentous algae from rocks, to deep sediment-rich habitats that require a more gracile feeding apparatus to sift through sediment on and between the rocks [28, 38]. First we compare the patterns of craniofacial modularity and within-module integration between *Tropheops* in shallow and deep habitats. We then examine rates of morphological evolution and disparity both between depths, and among modules of a given skeletal element. If *Tropheops* undergo multiple transitions between deep and shallow habitats, we expect these transitions to be accompanied by morphological change, due to differences in feeding behavior, and increases in the rate of morphological evolution in functionally important modules (i.e., muscle attachment sites). Similarly, we examine differences in disparity to assess whether specific depths facilitate the exploration of novel regions of morphospace, indicative of greater dietary range or more relaxed selection. Finally, we examine the role of plasticity in *Tropheops* morphological evolution by attempting to experimentally recapitulate the trends in morphology, modularity, and disparity observed in the natural populations.

We predict that *Tropheops* species exhibit a general conservation in the patterning of their craniofacial modules, and that this attribute is associated with the repeated evolution of ‘shallow’ and ‘deep’ ecomorphs. While we expect shallow and deep populations to exhibit similar rates of morphological evolution and disparity overall, we predict the fastest rates of morphological evolution should arise in those modules with the greatest functional importance. We also predict that we can mirror those patterns observed in natural populations via experimentally inducing plastic differences in morphology by raising *Tropheops* in conditions that mimic shallow or deep environments. We expect that plastic change in morphology will occur without concomitant changes in the pattern of modularity. Taken together, this would support that assertion that the cichlid craniofacial skeleton is characterized by robust patterns of modularity, despite differences in functional demands and morphology, and that this attribute may facilitate the rapid colonization of divergent habitats.

## Results

### *Tropheops* phylogenetic tree

Our Bayesian tree constructed using AFLP markers exhibited monophyletic groupings for the *Tropheops* and *Maylandia* species complexes within the *mbuna* (Figure S1; Table S1). Nodal support for many clades was typically low (i.e., posterior probabilities < 0.75). Low nodal support is not unusual for Malawi cichlids given their rapid radiation, and high hybridization rates [39]. Notably, support for monophyly of *Tropheops* ‘species’ is rare, and in many cases species are conspicuously polyphyletic. Exceptions to this include species from isolated/island populations such as Chinyamwezi and Chinyamkwazi. Groupings by locality are also rare. Different species from the same locality (e.g., Mazinzi Reef, “MZ” in Figure S1) are widely distributed across the phylogeny. However, replicate individuals from the same locality and species almost always cluster together, indicating the robustness of the genetic data. There are only three *Tropheops* taxa that are not monophyletic: *T. microstoma* from Domwe Island, *T. lilac* from Thumbi West, and *T. sp* “zebra mumbo” from Mumbo Island. Individuals from these species/populations group within a larger clade that appears to be more reticulate.

We also examined a number of tree statistics output from MrBayes. The MCMC run produced a high effective sample size (ESS), > 500 for all parameters, indicating that the trace contained few correlated samples, and represented the posterior distribution well. We also report a potential scale reduction factor (PSRF) value close to 1 for our convergence diagnostic, indicating that we have a good sample from the posterior probability distribution. We then discarded 25% of the initial trees for burn-in and randomly sampled 1000 trees from the Bayesian posterior distribution (BPD). We used the BPD trees for comparative methods that assessed differences in rates, disparity, and modularity.

### *Tropheops* morphology

**Fig. 2:**
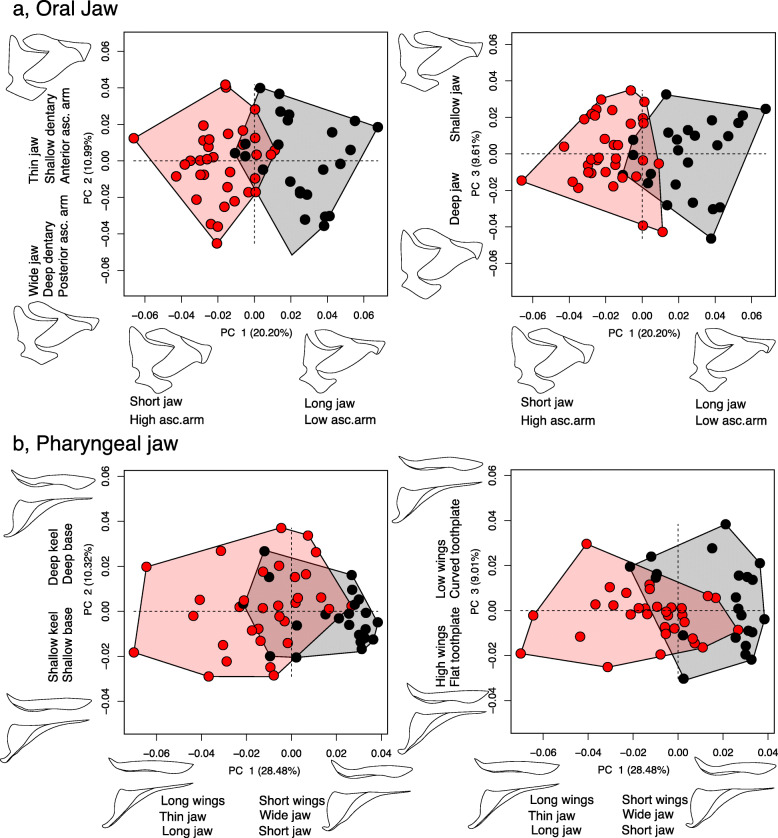
Morphospace occupation for natural *Tropheops* feeding bones from different depth regimes. Wireframe models reflect morphology at the extreme of a given axis. **a**, mandible PC1–3; **b**, lower pharyngeal jaw PC1–3. Red, individuals from shallow populations; black, individuals from deep populations

When species were grouped based on their occupation of either shallow or deep habitats, bone shapes exhibited some overlap, but generally occupied distinct regions of principal component (PC) morphospace (Fig. 2; Table S2, S3). Previous work has shown that shallow water species typically possess short, stout jaws to forage on clean, filamentous strands of algae, whereas deep water species are generally characterized by longer jaws to comb and shift loose material from sediment-covered substrate [28]. Our morphometric analyses confirm and extend this trend. We discuss results from the mandible and lower pharyngeal jaw in the main text, maxilla and pre-maxilla results can be found in the supplementary information.

#### Mandible

The first PC axis for the mandible characterized the height of the ascending arm and mandible length (20.20% of the variation). PC2 explained differences in the position of the ascending arm (anteriorly to posteriorly projected), the depth of the dentary, and the width of the mandible (10.99% of the variation). PC3 represented change in the lateral compression, or depth, of the mandible (9.61% of the variation). Mandible morphology differed based on occupation of deep or shallow habitats (pMANOVA; F = 16.6, *P* < 0.01), however much of this separation was confined to PC1 (Table S3).

#### Lower pharyngeal jaw

The first PC axis for the pharyngeal jaw reflected change in wing length, tooth-plate width, and jaw length (28.48% of the variation). PC2 explained differences in the depth of the jaw base and keel (10.32% of the variation). PC3 represented change in the wing height and the concave to convex curvature of the tooth plate (9.01% of the variation). Pharyngeal jaw morphology separated based on habitat depth (pMANOVA; F = 12.8, *P* < 0.01), with much of this separation confined to PC1 (Table S3).

### Comparative methods

Our phylogenetic tree was constructed using multiple *Tropheops* ‘species’ from different localities and across a wide range of depth regimes to characterize transitions between deep and shallow habitats. We used stochastic character mapping (SIMMAP) on our 1000 BPD trees to quantitatively assess transition rates between deep and shallow habitats [40, 41]. We found 11.3 transitions between deep and shallow habitats on average, with more changes occurring in the direction of shallow to deep habitats (7.9 transitions), rather than deep to shallow habitats (3.4 transitions).

### Modularity and integration

**Fig. 3:**
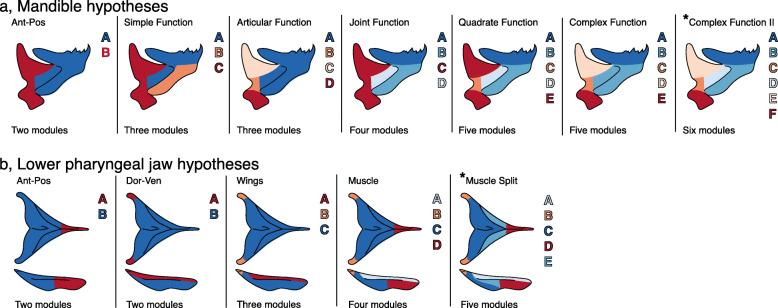
Partitioning schematics for competing modularity hypotheses. Colors reflect module partitions to be assessed by EMMLi. Letters correspond to the partitioning scheme (Table S4). The best fitting modularity hypothesis is illustrated by *. **a**, Mandible; **b**, lower pharyngeal jaw

We next examined the pattern of modularity and the strength of integration within modules in species from deep or shallow habitats to understand whether differences in feeding morphology are linked to divergent covariation patterns. We used the software package EMMLi [42] to assess the fit of different modularity hypotheses (Fig. 3; Table S4) in a phylogenetic framework with our 1000 BPD trees and recorded the frequency at which each modularity hypothesis was supported from populations in each habitat. Given modularity analyses can be biased by differences in sample size between populations, we also tested the sensitivity of our modularity models to uneven sample size as our shallow population had almost double that of the deep population. Evidence for retention of a single pattern of modularity between depths would suggest that modularity is robust to change despite the opposing functional demands associated with these different habitats. It would also provide another example of different morphologies evolving within the context of a conserved covariation structure (e.g., Fig. 1 [17, 18];). On the other hand, evidence for differences in the pattern of modularity between habitats would suggest that breaking of the covariation structure was involved in the evolution of these divergent eco-morphologies.

We found that the pattern of modularity was largely consistent between *Tropheops* from deep or shallow environments (Table 1; Table S5). In both groups, the best-fitting partitions were based on functional units including the attachment sites for muscles or ligaments, tooth bearing regions, and joints. This indicates that patterns of modularity within craniofacial bones (1) arise due to functional demands, (2) are robust to differences in habitat and foraging mode, and (3) are not associated with morphological divergence. While we found the pattern of modularity is similar between habitats, we found that the level of integration within modules differs between habitats, which suggests that eco-morphological divergence is associated with changes in the strength of covariation within modules.

**Table 1:** EMMLi output highlighting best supported modularity model(s) for the mandible and pharyngeal jaws in both natural and experimental populations. Additional competing models are presented if within two AICc units of the best supported model

Module Model	Population	Depth	K	AICc	Model Lik.	Post. Prob.
**Mandible**						
ComplexFunction.sep. Mod + same.between	Natural	Deep	7	− 6722	0.82	0.63
ComplexFunctionII.sep. Mod + sep.between	Natural	Deep	22	− 6719	0.34	0.20
ComplexFunctionII.sep. Mod + same.between	Natural	Shallow	8	−11,319	1.00	0.99
ComplexFunctionII.same. Mod + sep.between	Experimental	Benthic/Shallow	17	−13,989	1.00	0.75
ComplexFunctionII.sep. Mod + same.between	Experimental	Limnetic/Deep	8	−13,176	1.00	0.97
**Lower Pharyngeal Jaw**						
MuscleSplit.same. Mod + same.between	Natural	Deep	3	− 1057	1.00	0.94
MuscleSplit.sep. Mod + same.between	Natural	Shallow	7	− 1729	0.99	0.91
MuscleSplit.sep. Mod + sep.between	Experimental	Benthic/Shallow	16	− 1820	1.00	0.83
MuscleSplit.sep. Mod + same.between	Experimental	Limnetic/Deep	7	− 1763	1.00	0.99

#### Mandible

**Fig. 4:**
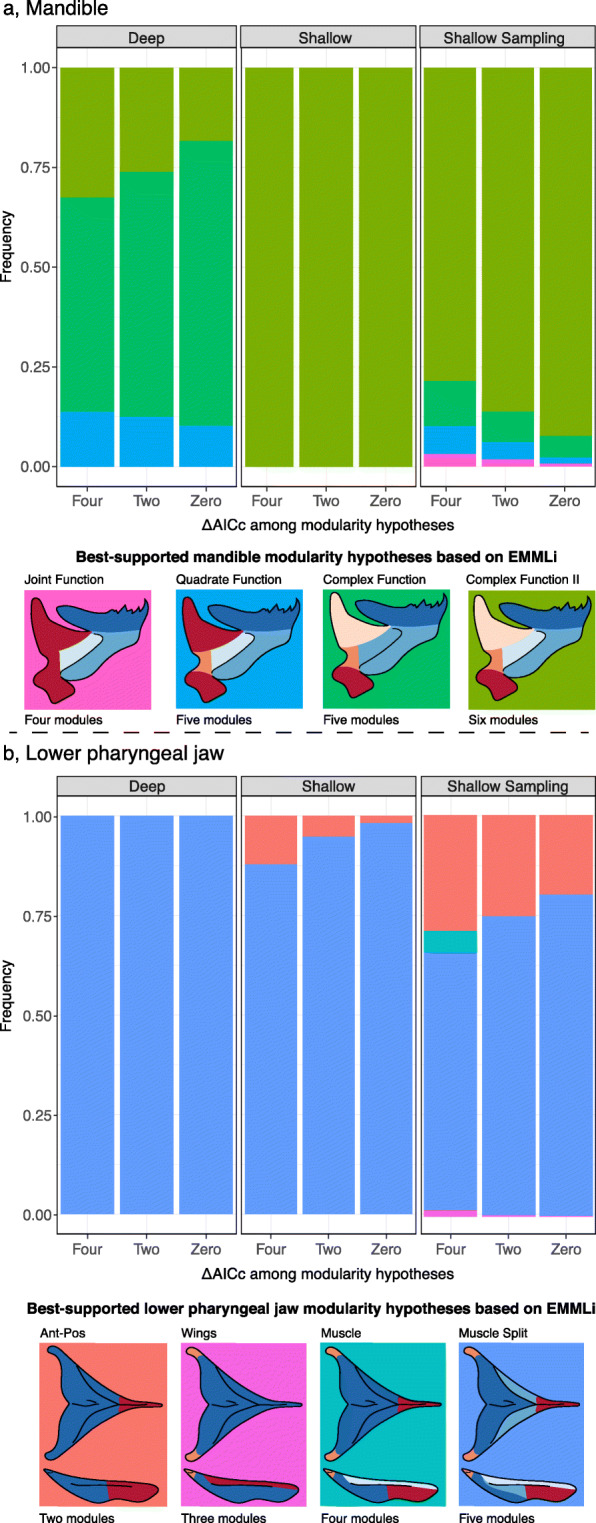
Support for competing modularity hypotheses between habitats as determined by EMMLi. **a**, mandible; **b**, lower pharyngeal jaw. The ‘Shallow Sampling’ plot reflects the frequency of module model selection derived from a sample size that matches the deep population. Colors used in the module model frequency plots are placed as background colors on module partition schematics that they represent

**Fig. 5:**
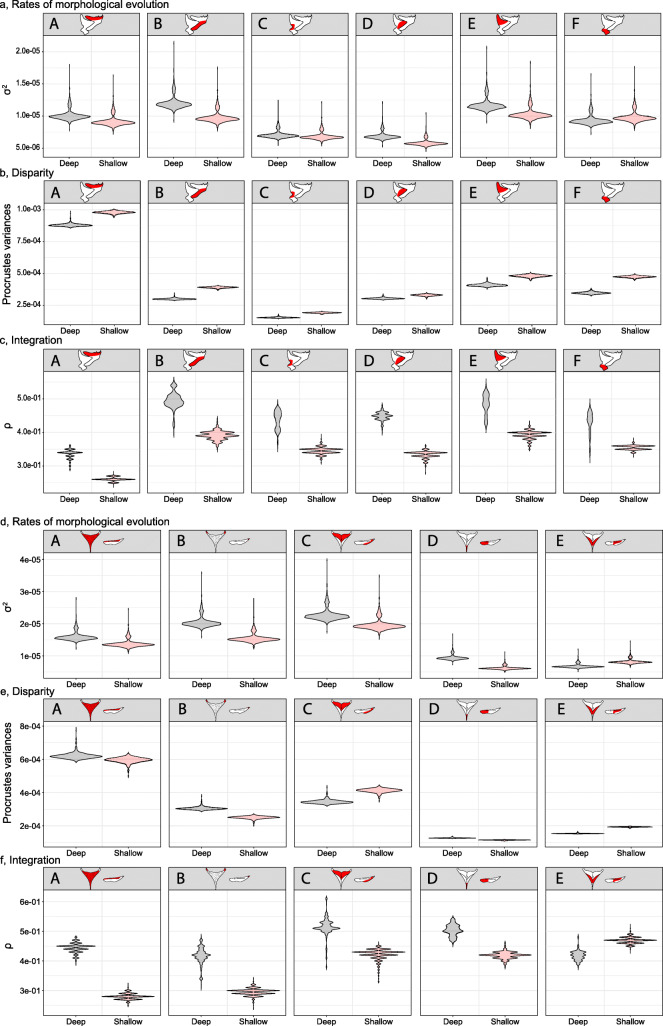
Violin plots depicting parameter values output from the rate of morphological evolution, disparity, and integration analyses across habitats for each module. Anatomical schematics illustrate the location of the module being tested in red. The depicted modules, and their associated letters, are based on the best-fitting modularity hypothesis determined by EMMLi. See Fig. 3 and Table S4 for full range of modularity hypotheses we tested. **a**-**c**, Mandible; **d**-**f** lower pharyngeal jaw. Red, shallow habitat; Black, deep habitat

For both shallow and deep datasets, a six module pattern was supported at the highest frequency for shallow individuals and ~ 30% of runs for deep individuals (Table 1, S5; Fig. 4a). This module-partitioning hypothesis breaks the mandible into a tooth-bearing region, lateral line canal, retro-articular process, quadrate-articular joint, ascending arm of the articular, and articular excurvation (Fig. 3a). This six-module pattern is highly similar to that reported by Parsons et al. [18] from a 2D landmark dataset in closely related *mbuna* species. In each habitat, EMMLi returned distinct values of integration (ρ) for both within- and among module comparisons for all six modules. Estimated values of ρ were highest for the lateral line canal and ascending arm modules (modules B and E respectively), lowest for the tooth-bearing module (module A), and intermediate for the quadrate-articular joint, articular excurvation, and retro-articular modules (modules C, D, and F respectively). Notably, *Tropheops* from deeper habitats exhibited relatively higher values of ρ (i.e., high levels of integration) within modules compared to species from shallow habitats (Fig. 5c; Table S6).

*Tropheops* from the deep habitat exhibited some variation in which modularity hypothesis was best supported. The five- and six-module models exhibited AICc scores within four units of each other in the deep sample, indicating overall similarity in model fit (Table 1). As a result, we cannot conclusively say which modularity model best-fits our mandible data. The five-module hypothesis differs from the six-module hypothesis in that it unites the articular excurvation and lateral line canal modules. The overall pattern is therefore fairly similar between the two hypotheses, and the ability of EMMLi to detect a difference between the five- and six-module hypotheses may be hindered by a small sample size in the deep taxa. Indeed, the five-module hypothesis was selected in ~ 50% of the models in taxa from the deep habitat, but when we assessed the sensitivity of our data to low sample size by sub-sampling the shallow taxa such that they mimic the sample size of the deep taxa, we find additional competing modularity hypotheses have increased levels of support (Fig. 4a).

#### Lower pharyngeal jaw

We found the pharyngeal jaw best fit a five-module hypothesis for both shallow and deep *Tropheops* members (Table 1, S5; Fig. 4b). EMMLi found support for partitioning into modules that include the tooth plate, pharyngeal wings, and attachment sites for the pharyngoclithralis internus, pharyngoclithralis externus, and pharyngohyoideus muscles (Fig. 3b). While there was some evidence for a two-module hypothesis in shallow taxa, reflecting partitions that divide the anterior and posterior portions of the lower pharyngeal jaw, this pattern arose in less than 10% of cases. The data may be somewhat sensitive to sample size, as additional modularity hypotheses gain support when we sub-sample the shallow taxa (Fig. 4b). Despite a small sample size in the deep populations, a five-module hypothesis was selected in 100% of the models. In both habitats, estimated values of ρ were typically highest for the three modules defined by muscle attachment sites (modules C, D, and E), and lowest for the tooth plate and pharyngeal wing modules (modules A and B respectively). Similar to the mandible, we found that the relative differences among module ρ were generally similar between deep and shallow species, but that *Tropheops* from deeper habitats exhibited higher values of ρ (Fig. 5f; Table S6).

### Morphological disparity and rates of evolution

Morphological disparity and rates of morphological evolution represent different measures of evolutionary potential (i.e., evolvability). Evidence for differences in disparity and/or rates between shallow- and deep-water habitats would suggest that evolvability of the feeding apparatus is influenced by foraging environment.

#### Morphological disparity

We detected no statistical difference in disparity for any feeding bones between *Tropheops* from shallow versus deep environments (Table S6). Similarly, we found no evidence for a difference in disparity between habitats when we compared subsets of landmarks defined by the best fitting modularity hypothesis suggested by EMMLi (Fig. 5b, e; Table S6). Thus, with respect to magnitudes of morphological variation, evolvability appears to be similar across shallow and deep foraging habitats. In spite of a lack of statistical difference, we note that for the mandible, disparity was consistently higher in the shallow population across modules (Fig. 5b). Distinct trends in the lower pharyngeal jaw were more difficult to determine, as disparity in shallow populations was higher in two of the five modules (Fig. 5e).

#### Rates of morphological evolution

Evolutionary rates were also compared across foraging habitats as well as between modules within bones (Table S6). For the former, we found no difference in rates of morphological evolution for craniofacial bones between species from shallow or deep environments. Further, no differences in rates were observed between depths when bones were partitioned into modules defined by EMMLi (Fig. 5a, d; Table S6). When the entire bone is considered, the results from both our disparity and rates analyses suggest that evolvability of the feeding apparatus is the same across foraging habitats. Again, despite the lack of statistical significance, we note deep populations typically exhibited faster rates of morphological evolution in both the mandible and lower pharyngeal jaw (Fig. 5a, d).

Alternatively, we observed statistically significant differences among several mandible and lower pharyngeal jaw modules (Table S7). In general, those modules representing bony processes or muscle attachment sites that have a direct association with feeding biomechanics evolved the most rapidly. For example, the ascending arm of the mandible (module E) is evolving more than 1.5x faster than the quadrate-articular joint (module C), (ascending arm, σ^2^ = 1.09 × 10^− 5^ (95% CI = 9.81 × 10^− 6^, 1.33 × 10^− 5^); quadrate-articular, σ^2^ = 6.94 × 10^− 6^ (95% CI = 6.30 × 10^− 6^, 8.55 × 10^− 6^); *p* < 0.01). Similarly, the pharyngeal jaw muscles attaching to the posterior surface were evolving almost three times as fast as muscles attaching to the anterior keel (pharyngoclithralis internus muscle attachment site (module C), σ^2^ = 2.08 × 10^− 5^ (95% CI = 1.88 × 10^− 5^, 2.56 × 10^− 5^); pharyngohyoideus muscle attachment site (module E), σ^2^ = 7.07 × 10^− 6^ (95% CI = 6.94 × 10^− 6^, 9.47 × 10^− 6^); *p* < 0.001). These data suggest that certain regions of the feeding apparatus exhibit greater evolutionary potential than others.

### Experimental recapitulation of shallow-deep water environments

Deep- and shallow-water foraging environments broadly mimic the benthic-limnetic eco-morphological axis that characterizes multiple cichlid radiations [17, 43, 44]. *Tropheops* from shallow water habitats generally possess benthic feeding morphologies in order to more efficiently forage on attached filamentous algae, whereas species that live at depth tend to possess more gracile, limnetic morphologies in order to suck and sift loose material from sediment covered rocks [28, 30]. Since the benthic-limnetic foraging axis can be re-created in the lab, we sought to test whether patterns of morphological divergence, modularity, and disparity observed across natural populations of *Tropheops* could be replicated within a single species reared under alternate foraging environments in the lab. If lab-reared *Tropheops* mimic the divergence, modularity, or disparity results found in the evolutionary sample, this would be consistent with a role for phenotypic plasticity in response to alternate kinematic demands in influencing patterns of evolutionary change. Alternatively, if patterns in lab-reared *Tropheops* do not match those from natural populations, this would suggest a larger role for genetic divergence driving evolution in this lineage.

#### Mandible morphology

**Fig. 6:**
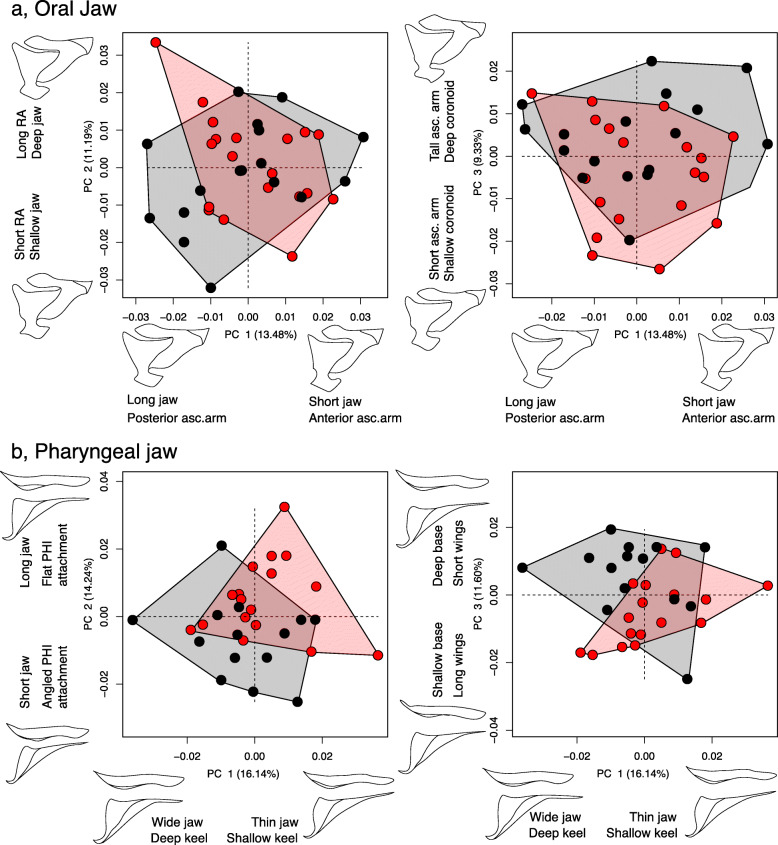
Morphospace occupation for experimental *Tropheops* feeding bones mimicking different depth regimes. Wireframe models reflect morphology at the extreme of a given axis. **a**, mandible PC1–3; **b**, pharyngeal jaw PC1–3. Red, benthic-shallow members; black, limnetic-deep members

The first PC axis for the mandible characterized differences in the position of the ascending arm (anteriorly to posteriorly projected), the length of the coronoid process, and the length of the mandible (13.48% of the variation). PC2 explained differences in the depth of the mandible, and RA length (11.19% of the variation). PC3 represented change in the height of the ascending arm and the height of the dentigerous portion of the mandible (9.33% of the variation). Unlike the natural populations, mandible morphology did not differ based on benthic or limnetic treatments (MANOVA; F = 1.73, *P* = 0.16), and there was substantial overlap in morphospace (Fig. 6a; Table S3).

#### Pharyngeal jaw morphology

The first PC axis for the pharyngeal jaw reflected change in the width of the tooth plate and depth of the keel (16.14% of the variation). PC2 explained differences in jaw length (14.24% of the variation). PC3 represented change in the depth of the jaw base (11.6% of the variation). Pharyngeal jaw morphology separated based on feeding treatment (MANOVA; F = 6.31, *P* < 0.01), with much of this separation confined to PC2 and PC3 (Fig. 6b; Table S3).

#### Modularity, integration, and disparity

While patterns of shape divergence between treatments were generally not the same as what was observed among natural populations (Table S8), patterns of modularity were highly similar. In particular, the experimental populations exhibited the same six-module hypothesis for the mandible and five-module hypothesis for the lower pharyngeal jaw as observed in the natural populations (Table 1, S9). We also found that a single pattern of modularity was favored regardless of whether the experimental population was subjected to an environment that mimicked a deep or shallow habitat. Thus, patterns of modularity within craniofacial bones appear to be highly robust among *Tropheops*.

We also found that the magnitude of within-module integration (ρ) differed between modules within a bone, and between modules across treatments (Table 2). Within the mandible, the greatest values of ρ were observed for the ascending arm in the deep/limnetic habitat, which is similar to what was observed in natural populations, although a larger difference in ρ was observed between treatments for this module in the experimental population. Another similarity between experimental and natural populations is that the lowest values of ρ were observed for the retro-articular and tooth bearing modules in lab-reared *Tropheops*. Across treatments, the experimental population also mirrored trends observed in natural populations insofar as deep/limnetic animals exhibiting generally higher levels of ρ relative to shallow/benthic animals.

**Table 2:** Comparison of within-module disparity and integration between depths for the mandible and pharyngeal jaw in the experimental populations

Module	Depth	Disparity (PV)	P-Value	Integration (ρ)
**Mandible**				
Full	Deep	1.60E-03	0.729	0.27
	Shallow	1.50E-03		0.25
A	Deep	5.46E-04	0.259	0.26
	Shallow	4.80E-04		0.24
B	Deep	1.74E-04	0.557	0.3
	Shallow	2.01E-04		0.28
C	Deep	1.46E-04	0.474	0.29
	Shallow	1.78E-04		0.28
D	Deep	2.11E-04	0.773	0.29
	Shallow	2.30E-04		0.27
E	Deep	2.22E-04	0.911	0.37
	Shallow	2.12E-04		0.26
F	Deep	2.61E-04	0.169	0.22
	Shallow	1.98E-04		0.21
**Lower Pharyngeal Jaw**				
Full	Deep	1.10E-03	0.293	0.32
	Shallow	1.00E-03		0.39
A	Deep	4.33E-04	0.098	0.33
	Shallow	2.94E-04		0.36
B	Deep	1.35E-04	0.171	0.25
	Shallow	9.68E-05		0.19
C	Deep	2.24E-04	0.352	0.5
	Shallow	2.77E-04		0.43
D	Deep	1.56E-04	0.473	0.4
	Shallow	1.27E-04		0.42
E	Deep	1.83E-04	0.593	0.52
	Shallow	1.62E-04		0.49

Finally, we found no differences in disparity between *Tropheops* individuals raised in environments that mimic deep or shallow habitats (Table 2). Similarly, we found no differences in disparity when we partition our landmarks into those modules defined by EMMLi and then compare disparity between depths (Table 2). These trends mirror those observed in the natural *Tropheops* populations (i.e., Fig. 5, Table S6). For example, the tooth-bearing module exhibited the highest disparity across treatments/environment for both the mandible and lower pharyngeal jaw.

In general, while differences were noted, trends in shape, disparity, and modularity were broadly similar between lab-reared and natural populations of *Tropheops*. These observations are consistent with the hypothesis that plasticity plays an important role in facilitating evolutionary divergence in response to selection for distinct foraging habitats (e.g., [45]).

## Discussion

### Many species, two ecomorphs

Lake Malawi hosts the greatest number of cichlid species of any of the East African Rift Valley lakes, and within this species-flocks, *Tropheops* exhibit one of the highest speciation rates [37]. Following the construction of an AFLP phylogenetic tree using a subset of *Tropheops* from the southern portion of the lake, we find that the evolution of this species complex may be characterized by multiple transitions between deep and shallow environments (approximately 11 transitions). *Tropheops* residing in more shallow habitats exhibited a more robust feeding apparatus, while those *Tropheops* members residing in deeper habitats exhibited a more slender and gracile feeding apparatus [38]. The specific differences in morphology are predicted to influence feeding performance and biomechanics [46, 47], and include the area for muscle or ligament attachment sites (i.e., increased maxillary shank area for A1 attachment, increase pharyngeal jaw keel for pharyngohyoideus attachment), as well as the length of bony process that would have the effect of changing the mechanical advantage (i.e., the ascending arm of the mandible, the wings of the pharyngeal jaw). These data formalize and extend previously published trends (e.g., [28, 30]), and suggest the existence of two general *Tropheops* ecomorphs within Lake Malawi.

### Two ecomorphs, one covariance structure

While we observed conspicuous differences in morphology between shallow and deep habitats, we found little evidence for differences in patterns of modularity within any bone examined. These results are consistent with previous studies [18], and suggest that patterns of cichlid craniofacial modularity can be robust to changes in habitat and functional demands. They also suggest that morphological diversification in *Tropheops* does not require ‘tinkering’ of an underlying pattern of covariation to produce adaptive phenotypic change. Indeed, recent evidence suggests there may be a finite amount of modularity patterns possible in the teleost skull [19], indicating a general conservation of covariation patterning could be common among taxa [7].

One possible evolutionary consequence of a conserved pattern of modularity is the ability of a lineage to undergo multiple transitions between discrete environments (e.g., [48]). Results from our stochastic character mapping supports this hypothesis, and suggests that transitions are fairly common and can occur in either direction between deep and shallow habitats. Seehausen [27] theorized that repeated transitions between these two habitats across cichlid lineages may occur due to ecological character displacement. In this model, a cichlid population would undergo intense local resource competition driving disruptive selection from shallow habitats toward deeper habitats. Subsequently, reproductive isolation would occur via depth-induced changes in signaling phenotypes (e.g., coloration due to visual sensitivities at different depths [49]). As resource competition grows in the deeper population, a transition back to the shallows may occur, and so on. We hypothesize that this iterative transitioning between habitats is aided by the conservation of craniofacial modularity. Felice et al. [50] used the metaphor of a fly in a tube to describe how trait covariation can influence morphological evolution, and demonstrated how a population can move around in morphospace, exhibiting different trait values, under a given covariational structure. Changing the pattern of covariation will, 1) take time, as this would likely involve the accumulation of multiple mutations over a period of generations, and 2) will change the range of morphologies that can evolve. Retaining a single pattern of covariation between habitats could allow morphological evolution to occur more rapidly (if the direction of selection aligns with the pattern of covariation [9]), but only if the limits of the morphospace includes those adaptive peaks for each habitat.

Alternatively, it is possible that a seemingly conserved pattern of modularity is a secondary consequence of shape being more evolvable than modularity. For instance, it is possible that *Tropheops* members have repeatedly and rapidly transitioned between shallow and deep environments due to fluctuating water levels in Lake Malawi. In this scenario, divergent patterns of modularity may not be able to evolve in such short time intervals between habitat transitions [51].

### Evolvability of the feeding apparatus is consistent across habitats but distinct among anatomical modules

Rates of morphological evolution and magnitudes of disparity represent different metrics of evolutionary potential (e.g., evolvability), and we find that both measures are similar between habitats, indicating that neither deep nor shallow habitats have the capacity to constrain or facilitate morphological evolution. However, when comparing modules within bones, notable differences in evolutionary potential arise. Specifically, modules differ with respect to both rates and magnitudes of evolutionary change. In general, those regions of the anatomy more directly associated with muscle and ligamentous input evolve at a higher rate than modules with no obvious functional role. This trend is consistent with the adaptive value of these traits with respect to feeding kinematics. Indeed, the trend in functionally relevant components of anatomical structures exhibiting fast rates and high evolvability has been noted in other teleosts groups (i.e., Electric fishes [52, 53], cichlids, pomacentrids, centrarchids, and labrids [54]), and may reflect a general trend across organisms [55, 56]. In terms of disparity, it is notable that the tooth-plate module exhibited the highest levels for both the mandible and lower pharyngeal jaw. Given the ability of dentition to respond plastically to shifts in diet [57, 58], this trend suggests an important role for phenotypic plasticity in promoting disparity in the cichlid feeding apparatus.

### Evolvability is not associated with the magnitude of phenotypic integration

We also document differences in the magnitude of phenotypic integration (ρ) between modules within bones. Differences in the strength of integration are predicted to have the capacity to either constrain or facilitate morphological evolution based on the direction of selection [59]. However, there is as yet no consensus for how (or whether) phenotypic integration influences evolutionary potential. Empirical studies looking at the association between evolutionary rates and integration have produced mixed results, ranging from no association [7], to positive [60] or negative associations between the two [10]. Simulation and theoretical studies have demonstrated that while morphological disparity can be governed by the strength of integration, more uncertainty surrounds the association between rates of morphological evolution and integration [7, 50]. Our results are more in line with these recent simulations. Specifically, they show little to no relationship between phenotypic integration and evolutionary rates or disparity. The only exception to this general trend is observed within the mandible (Fig. 5a-c), where there appears to be a negative correlation between disparity and integration; however, this association is largely driven by the tooth-bearing module which exhibits the highest level of disparity and lowest integration. Additionally, when we compare module parameters between habitats we find that, on average, taxa from the deeper habitats exhibit stronger within-module integration and faster rates of morphological evolution. Trends in disparity are more variable among feeding bones for a given module; shallow taxa typically exhibit greater mandible disparity, while deep taxa typically exhibit greater lower pharyngeal jaw disparity.

### Plasticity plays a large role in influencing patterns of evolutionary change

By experimentally subjecting a shallow dwelling *Tropheops* species to environments that mimic either a shallow or deep habitat, we were able to replicate patterns detected across the *Tropheops* species complex. In other words, *Tropheops* species appear capable of craniofacial remodeling that mirrors variation observed at the species level. In terms of morphology, similar aspects of shape are being affected, despite the fact that they sometimes load on different PC axes (e.g., height of the ascending arm loads on PC1 for natural populations and PC3 in experimental populations). We find it especially notable that lab-reared populations of a single species, forced to feed using alternate strategies, possess the same pattern of modularity described for the larger subsample of the *Tropheops* species complex. These data suggest that modularity within craniofacial bones appears robust between natural and laboratory environments, and between foraging habitats and behaviors. Finally, many similarities are also observed in terms of within-module levels of integration and disparity. The conservation of these morphological patterns illustrates how plasticity in the feeding apparatus can be responsive to change within a specific covariation structure that promotes the accumulation of morphological variation in areas that would have direct biomechanical implications for feeding. If plasticity can replicate the patterns we observe in the natural population, this suggests that the environment has the capacity to play a large role in shaping *Tropheops* phenotypic evolution, at least during early stages of divergence. Empirical studies have demonstrated that phenotypic plasticity facilitates adaptive phenotypic diversification [61] and can vary in its effects among taxa [62]. Thus, plasticity may help to expedite transitions between depths allowing them to be ‘morphologically primed’ for diversification.

While plasticity can assist in this morphological divergence, it is likely that the occupation of a different habitat following a transition would also require some genetic and developmental evolution. We find evidence for this based on the larger magnitude of divergence between the shallow and deep members from the natural population relative to the experimental populations. It appears that plasticity cannot match the range of morphologies present in the natural populations, at least under the conditions we provided. Previous studies have also shown differences in the allele frequency of *ptch1*, a gene involved in directing the development of bones involved in the feeding system, among *Tropheops* members from different depth regimes [63, 64]. These studies suggest that genetic differentiation could underlie some of the differences observed in the natural populations of *Tropheops*, and plasticity is not solely responsible for differences between the natural populations. Indeed, previous work has demonstrated variability in the ability of closely-related cichlids to plastically remodel the craniofacial skeleton in response to alternate feeding regimes, whereby ecological specialists exhibited little response, while generalists exhibited a marked, and predicable, morphological response [62]. As such, in more ecologically generalist taxa such as *Tropheops* there may be a benefit to retaining plasticity in order to facilitate rapid colonization of new niches, and the transition between niches, rather than becoming genetically canalized [65]. By possessing a feeding system that is readily able to respond to biomechanical stimulus and remodel itself, *Tropheops* can avoid intense competition with both congeners and other ecological specialists. As a consequence, this may allow *Tropheops* to exploit a much greater spectrum of depth regimes. Thus, plasticity appears an important first step toward an evolutionary response [45].

## Conclusion

Cichlid fish from the East African rift lakes are characterized by their extraordinary taxonomic and phenotypic diversity. Among the lakes, depth is considered a major axis of trophic niche partitioning among various cichlid populations [28, 66]. Habitat partitioning is the first component to many adaptive radiations [67], and the wealth of freshwater reefs, shallow bays, and isolated island environments provide ample ecological opportunity for cichlids to diversify along a habitat (i.e., depth) gradient [27]. Here we construct a phylogenetic tree focused primarily on a single species complex *Tropheops*, known to occupy multiple depth regimes in Lake Malawi, to examine how depth has shaped morphological evolution in the feeding apparatus. We found that selection drives feeding bone morphology toward different shapes based on their occupation of either shallow or deep environments. Despite differences in morphology we found no difference in disparity, rates of morphological evolution, or the pattern of modularity between *Tropheops* members residing at different depths. This indicates *Tropheops* exhibit a conserved pattern of modularity between depths, despite differences in morphology, a feature that may have facilitated the rapid depth transitions and subsequent colonizations. However, we do find differences in phenotypic integration and rates of morphological evolution among our modules, and these differences appear are most pronounced in those modules with functional roles (i.e., muscle attachment sites). Functionally salient modules are candidates for the greatest amount of morphological change given they must remodel in response to the differing biomechanical conditions experienced between the two depth regimes. Taken together, our data represent a rare example of morphological divergence coupled with module pattern conservation, indicating that multiple adaptive phenotypes can arise from a single pattern of covariation.

## Methods

### Specimen collection

We collected 58 individuals from across the *Tropheops* species complex from 12 different localities in the southern part of Lake Malawi during two field trips in 1996 and 2001. Our *Tropheops* sampling strategy obtained taxa from a wide depth gradient (deep, *n* = 24; shallow, *n* = 34). We skeletonized our specimens and then extracted four bones critical for food capture and processing (mandible, pharyngeal jaw, maxilla, and pre-maxilla). To gain 3D models of our bones of interest we used micro computed tomography (μCT) and scanned all specimens at 20–25 μm resolution at 90 kV and 75 μA. All μCT scans were performed using an X-Tek HMXST 225 μCT scanner (Nikon Corporation). We segmented the bones using Mimics v19 (Materialise NV), and then exported the 3D models for morphometric analysis.

We gathered a total of 136 individuals from multiple Lake Malawi cichlid genera along the southeast arm of Lake Malawi for phylogenetic analysis. Our sampling strategy focused on the subgenus *Tropheops tropheops*, and included 90 *T. tropheops* individuals from ~ 20 subspecies. Based on detailed transects performed by Ribbink et al. [38], we were able to comprehensively sample the *Tropheops* species complex from the southern part of Lake Malawi, implying that our results should provide a good estimate of the habitat transition rate. Many members of the *T*. *tropheops* species complex lack formal description, therefore species identification and nomenclature follow Ribbink et al. [38] and Konings [68]. We added a number of Lake Malawi cichlids to our sample from outside the *T. tropheops* complex including 18 *Maylandia* from four species and 17 non-mbuna from five genera. We also included two *Tropheus duboisi* individuals from Lake Tanganyika, which were purchased through the aquarium trade, bringing the total to 138 individuals (Table S1). We collected tissue samples from live fish via pectoral fin clips of each specimen and stored them in 95% EtOH prior to DNA extraction. Following DNA extraction, all specimens were skeletonized and stored. Whenever possible, individuals used in the phylogenetic analysis were also used as part of the morphological assessment.

### Phylogenetic tree construction

We extracted genomic DNA from the fin clips of 1–3 individuals of each taxon by phenol-chloroform extraction. We used Amplified Fragment Length Polymorphisms (AFLP) to generate a character matrix for phylogenetic analysis. AFLP is a DNA fingerprinting technique that characterizes thousands of restriction polymorphisms spread throughout the genome [69]. Genomic DNA was first double-digested using two restriction enzymes EcoRI and MseI. Double stranded adapters are then ligated onto the overhanging ends of the fragments. An initial PCR reaction amplifies a subset of fragments that match adapter primers containing an additional nucleotide (EcoRI-A and MseI-C). The product of this first amplification is then used as the template for a further 18 different amplifications performed with primers containing an additional 2-nucleotide extension (E-ACA, M-CAA, M-CAG, M-CTA, M-CTT; E-ACC, M-CAA, M-CAC, M-CAT, M-CTA; E-ACT, M-CAG, M-CAT, M-CTA, M-CTG, M-CTT; E-AGC, M-CAG, M-CAT, M-CTA, M-CTG, M-CTT). For detailed restriction-ligation and PCR protocol information see [69, 70].

Fragments were separated using a Beckman Coulter CEQ 8000 capillary sequencer. Peaks were scored using a quartic model with a slope threshold of 2.0% and relative peak height of 5.0%. Bands were scored as present/absent using Beckman Coulter’s Fragment Analysis Module. The presence of each fragment was confirmed manually. Fragments between 80 and 500 bp in size were binned (1 nucleotide bin width) using Beckman Coulter’s AFLP Analysis Software. The binary output was imported to an Excel spreadsheet and formatted for MrBayes v3.2.6.

We performed a Bayesian phylogenetic analysis of our AFLP data in MrBayes v3.2.6 [71] running on the Cipres computing environment [72]. Our complete dataset contained 7953 binary characters scored for 138 individuals, with 90 individuals coming from the *Tropheops* species complex. We used the F81-like restriction site model intended for analyzing restriction fragment data with the ‘noabsencesites’ coding bias (characters that are absent in all taxa are unobservable) correction parameter [73]. While this binary state model implemented in MrBayes is a simplistic model of the actual genetic processes operating on the evolution of AFLP markers, methods which provide a more accurate representation of AFLP marker evolution, are computationally demanding for larger datasets [74, 75]. We set the Dirichlet prior for the state frequencies to 0.73, 0.27 matching the empirical 0/1 frequencies in the dataset and selected gamma variation across sites. We set our outgroup taxa as *Tropheus duboisi* from Lake Tanganikya, and forced monophyly for all ingroup Lake Malawi taxa. We then placed two topology constraints on our tree. We placed constraints to force monophyly in the node leading to all non-mbuna (mostly sand dwellers) and *mbuna* (rock dwellers), and then forced monophyly at the node leading to all Mbuna. To estimate the posterior distribution of our model parameters and calculate tree topology we performed two runs of a Markov chain Monte Carlo (MCMC) analysis for 2 million generations with ten chains (one cold, nine hot) at a chain temperature of 0.1 and sampled every 200 generations. We assessed stationary distributions based on the potential scale reduction factor (PSRF) and effective sample size (ESS) statistics reported in MrBayes [76]. We discarded the first 25% trees for burn-in, based on the stationary distributions reviewed in Tracer v1.7 [77].

We then randomly sampled 1000 post burn-in AFLP trees from the Bayesian posterior distribution (BPD) to account for phylogenetic uncertainty in all future comparative method analyses. These 1000 BPD trees were then converted into ultrametric trees. To force the AFLP trees to become ultrametric we used a penalized likelihood method contained in the chronos function from the R package ape [78]. Chronos uses a penalized likelihood method to convert branch lengths from the number of substitution per site to time. We set one calibration point at the split between *Rhamphochromis* and the rest of the Lake Malawi radiation using soft bounds of 1–2 million years, corresponding to the age of Lake Malawi [79].

To assess the transition rate between deep and shallow habitats we applied stochastic character maps (SIMMAP) to each of our 1000 BPD trees [40, 41] using the R package phytools v.0.6–44 [80]. We then calculated the average number of transitions between habitats, and examined the direction of these transitions to ascertain whether shallow to deep, or deep to shallow transitions occurred more frequently.

### Geometric morphometric analysis

**Fig. 7:**
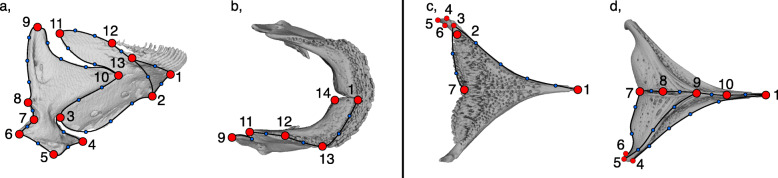
Landmarking scheme for *Tropheops* feeding bones. **a**, mandible lateral view; **b**, mandible dorsal view; **c**, pharyngeal jaw dorsal view; **d**, pharyngeal jaw ventral view. Red circles, fixed landmarks; blue landmarks, semi-landmark curve positions

We placed landmarks (LMs) across all four bones to best characterize functionally and developmentally relevant aspects of shape change occurring in the *Tropheops* species complex (Fig. 7; Figure S2; maxilla: 4 fixed LMs, 40 semi-LMs; pre-maxilla: 5 fixed LMs, 20 semi-LMs; mandible: 14 fixed LMs, 95 semi-LMs; pharyngeal jaw: 10 fixed LMs, 35 semi-LMs). We present results for the maxilla and pre-maxilla bones in the supporting information (available online), and the mandible and lower pharyngeal jaw results in the main text, as these two bones have been the focus of many previous studies into trophic evolution and plasticity in cichlids. All landmark data was collected using Landmark v3.6 [81] and processed using the geomorph package v3.0.7 [82] in R v3.5.1 [83]. We used the *digit.curves* function in geomorph to resample and array our semi-landmarks equidistantly along a curve between two fixed landmarks [84]. Following the placement of landmarks we performed a Procrustes superimposition to remove the effects of size, translation, and rotation such that the landmark configurations are in register [85]. To investigate the effects of allometry on our shape data we performed a Procrustes ANOVA between centroid size and shape for each bone. We found a significant effect of allometry for two bones (mandible: r^2^ = 0.050; *P* < 0.001; lower pharyngeal jaw: r^2^ = 0.053; *P* = 0.006). To remove the effects of allometry on all of our bones, we performed a regression of shape on geometric centroid size to generate a landmark data set based on residuals. Following allometric correction, we calculated mean shape configurations for each operational taxonomic unit (OTU) for use with future comparative methods, with each OTU represented by approximately three individuals (deep, OTU *n* = 8; shallow OTU *n* = 14). We then conducted a principal components (PC) analysis on all landmark configurations to reduce the shape data into a series of orthogonal axes that best explained the major variation in bone shapes among *Tropheops* taxa. We extracted five PCs from each bone, as subsequent PC axes accounted for < 5% of the variation in shape (Table S2). We constructed morphospaces for each bone from the first three PCs, and colored each individual based on their depth assignment, either shallow or deep, to illustrate the morphological variation present in the sample.

### Modularity, integration, and disparity

#### EMMLi

We used EMMLi (evaluating modularity with maximum likelihood) to simultaneously test multiple modularity hypotheses based on different landmark partitions in our *Tropheops* taxa (Fig. 3), and then assess the strength of covariation within those modules [42]. The EMMLi method compares a suite of modularity hypotheses using AICc to determine the best fitting modularity hypothesis. EMMLi extracts a vector based correlation matrix with size determined by the number of landmarks. This symmetric matrix provides a single number for covariance between landmarks, as opposed to one for each dimension, and are considered more biologically meaningful as each landmark is considered a separate trait [42]. We computed two vector correlation matrices for each bone; one matrix calculated using *Tropheops* taxa from the deep habitat and another matrix for those from a shallow habitat. EMMLi tests the fit of different partition hypotheses by allowing modules to either constrain or vary the correlation coefficient (ρ) within and among different modules. To correct for the evolutionary associations among our taxa, we used the phylogenetic independent contrasts (PICs) of shape in the EMMLi analyses [86, 87]. PICs of shape were generated from the 1000 post burn-in AFLP trees and used to derive a distribution of modularity analyses and ρ values. We used these 1000 EMMLi analyses to determine how frequently, and how strongly (based on AICc score), competing modularity hypotheses were selected across both habitats. Given the discrepancy in sample size between deep and shallow taxa (deep, OTU *n* = 8; shallow OTU *n* = 14), we randomly sampled individuals from our shallow population without replacement to mimic the sample size of the deep population 1000 times. To determine how sensitive our results were to sample size differences we then re-ran our EMMLi analysis on the ‘sampled’ shallow population and compared this with results from the original shallow population. The frequency of incorrectly selected modularity models will give an indication into how sensitive the data are to sample size.

Module partition hypotheses varied from more simplistic models whereby modules are split into anterior-posterior or dorsal-ventral landmark partitions, to more complex models that attempt to capture regions of functional importance, such as sites of muscle or ligament attachment, or regions where bones articulate (see Table S4 for all partition hypotheses). We performed the EMMLi analysis on two different datasets with the intention of investigating any similarity or differences in the best-supported partitions. The first analysis contained all *Tropheops* individuals sampled from the lake (natural population), and a second analysis contained a single species of *Tropheops* from a feeding trial experiment (experimental population, see the ‘Dietary plasticity’ section below). Differences in partitions between depths would suggest habitat might have differentially influenced the covariation structure of our bones, which would have implications for evolvability and differences in function or/and development.

### Comparative methods

As we often had multiple individuals for each *Tropheops* ‘species’, we calculated a mean landmark configuration and PC score for each species. We performed all subsequent comparative methods on these mean data. For all comparative methods we pruned any tips from the tree where we lacked trait data, which left a total of 22 *Tropheops* taxa: 14 from the shallow environment and 8 from the deep. All comparative methods were performed using a random sample of 1000 post burn-in AFLP trees to account for phylogenetic uncertainty and generate distributions of output parameters.

#### Phylogenetic MANOVA

We used a phylogenetic multivariate analysis of variance (pMANOVA) to test for differences in bone shape between *Tropheops* from shallow and deep habitats. The test statistic for the pMANOVA was calculated using the first five PC scores from each bone and compared to a null distribution of PC scores. We generated a null distribution by simulating 1000 new dependent variables based on the rate matrix obtained from the phylogenetic tree. The pMANOVA was conducted using the R package Geiger v.2.0.6 with the *aov.phylo* function [88].

#### Disparity

We compared the disparity of *Tropheops* taxa residing in either shallow or deep environments using Procrustes variance (PV). PV measures the distribution of taxa around the mean shape for a given habitat grouping. We used the *morphol.disparity* function in the R package geomorph to conduct the PV analysis [82]. During the disparity calculation for the mean *Tropheops* shapes we used landmark configurations that retained their allometric shape components and added in centroid size as a variable in the model. Phylogeny was accounted for in the model following the use of the *procD.pgls* function in the R package geomorph. Disparity is therefore calculated using the residuals obtained from a phylogenetic least squares estimation of coefficients, instead of the ordinary least squares estimation used in the standard PV analysis on the experimental *Tropheops* bone shapes. We also repeated the disparity analysis using subsets of landmarks defined by the best fitting modularity hypothesis determined by EMMLi and compared module-specific disparity between habitats. Differences in disparity between habitats would suggest some type of morphological constraint or morphological opportunity is operating on one habitat relative to the other, or would imply some differences exist in developmental robustness.

#### Rates of morphological evolution

We used the function *compare.evol.rates* in geomorph to compare the rate of morphological evolution between habitats using a complete landmark dataset for each bone, and a landmark dataset subset by the best-supported modularity hypotheses defined by EMMLi [89]. Note that we are limited to assuming a Brownian Motion (BM) model of evolution for this analysis, however the results returned should be conservative relative to a model of evolution that more closely fits our data.

We used the function *compare.multi.evol.rates* in the R package geomorph to compare the rate of morphological evolution among different modules in our mandible and lower pharyngeal jaw data sets under a BM model of evolution. Significance is determined via simulating tip data under BM and comparing this to the empirical data [90].

#### Dietary plasticity

To assess whether trends in the pattern of modularity and morphological disparity observed in the natural populations can be replicated via phenotypic plasticity, we conducted a feeding trial experiment to replicate the conditions experienced in deep or shallow environments using a single species, *Tropheops* ‘red cheek’ (TRC). While TRC naturally resides in more shallow habitats, previous studies have demonstrated that this ectomorph can exhibit a plastic phenotype [32]. We divided our experimental population of TRC into two tanks at 1 month old and subjected them two diet treatments that differed in the biomechanical demand placed on the feeding system to mimic the shallow-deep habitats. We raised 19 individuals in a shallow environment habitat whereby individuals consumed puréed algae flake food mixed with 1.5% food-grade agar spread over two lava rocks fed once per day. TRCs in the shallow environment must use a ‘tooth scraping’ feeding mode to separate the algae from the rocks, a feeding behavior that is considered more biomechanically challenging. We also raised 17 individuals in an environment mimicking a deep habitat. TRCs in the deep environment were provided with the same amount of food but ground down and sprinkled into the tank and fed once per day. This forced the TRCs to exhibit a greater degree of suction and ram feeding behaviors to consume their food, a feeding behavior that is considered less biomechanically challenging [32]. The feeding trial continued for 5 months on a 12 h light-dark cycle in water conditions mimicking that of Lake Malawi. Upon completion, all animals were euthanized by overdose with tricaine methanesulfonate (Aquatic Ecosystems Inc.), fixed overnight at room temperature in 4% paraformaldehyde (Sigma) in 1x phosphate-buffered saline, and stored in 70% ethanol. The Institutional Animal Care and Use Committee (IACUC) at the University of Massachusetts Amherst and the University of New Hampshire approved all protocols.

We μCT scanned and landmarked all experimental fish as described in the ‘Specimen collection’ and ‘Geometric morphometric analysis’ sections above. We placed landmarks for all individuals using the same schemes used in the evolutionary analysis and again tested for allometry in our bones of interest. We found a significant effect of allometry on our shape data (mandible: r^2^ = 0.158; *P* < 0.001; lower pharyngeal jaw: r^2^ = 0.207; *P* < 0.001). We removed the allometric component of shape as described above to generate a landmark data set based on residuals. Finally, we replicated the EMMLi, disparity, and MANOVA analyses described above in the experimental populations to assess differences between those individuals raised in deep versus shallow conditions. Note that we used non-phylogenetic versions of the MANOVA and disparity analyses as we are examining the plastic response of a single *Tropheops* species.

## Supplementary information

**Additional file 1.**

**Additional file 2.**

**Additional file 3.**

## Data Availability

All morphometric data and phylogenetic trees are contained in the supplementary information.

## Abbreviations

AFLP: Amplified Fragment Length Polymorphism
AICc: Akaike Information Criterion corrected;
BM: Brownian Motion
BPD: Bayesian Posterior Distribution
EMMLi: Evaluating Modularity with Maximum Likelihood
ESS: Effective Sample Size
IACUC: Institutional Animal Care and Use Committee
LM: Landmark
MANOVA: Multivariate Analysis of Variance
MCMC: Markov Chain Monte Carlo
OTU: Operational Taxonomic Unit
PIC: Phylogenetic Independent Contrasts
PC: Principal Component
PCA: Principal Component Analysis
PV: Procrustes Variance
PSRF: Potential Scale Reduction Factor
TRC: *Tropheops* ‘red cheek
μCT: Micro-Computed Tomography
