# Supplementary material for: Ecomorphological divergence and habitat lability in the context of robust patterns of modularity in the cichlid feeding apparatus
Source: BMC Evol Biol. 2020 Jul 31;20:95. doi: 10.1186/s12862-020-01648-x (PMC7393717; doi:10.1186/s12862-020-01648-x)

Figure S1. AFLP Tree

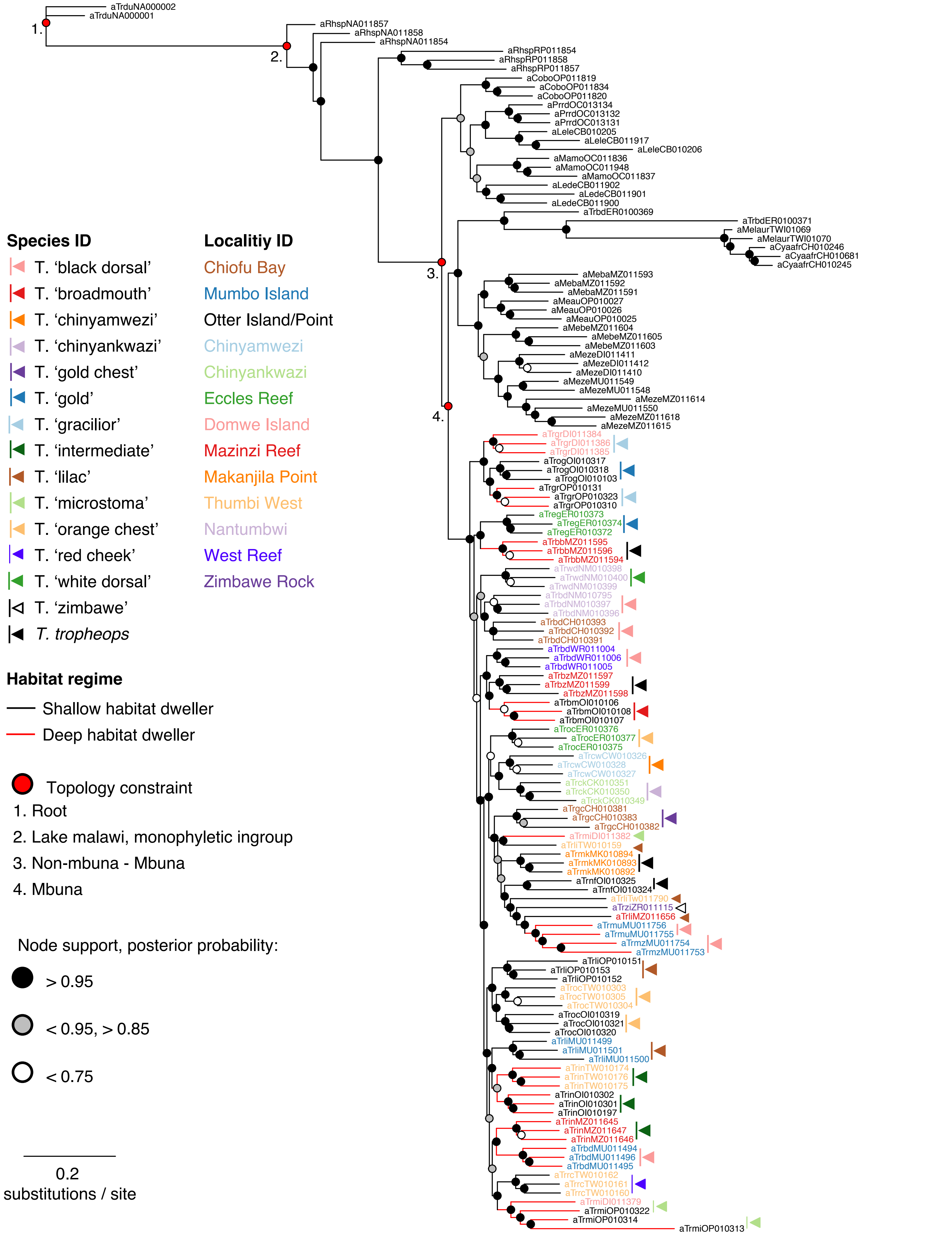

Figure S2. Maxilla and Pre-maxilla Landmarking Schemes

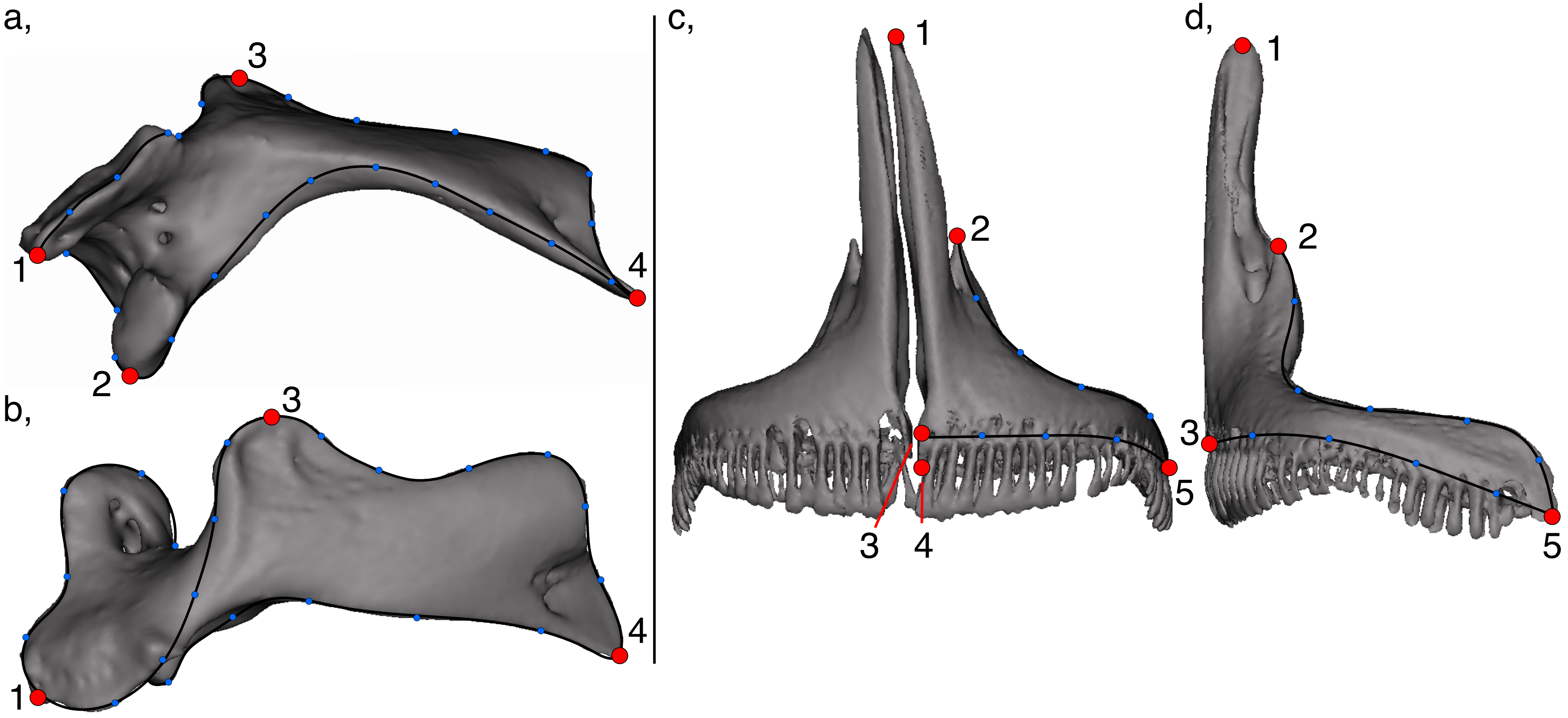

Figure S3. Natural Population Morphospaces

a, Maxilla - Individuals

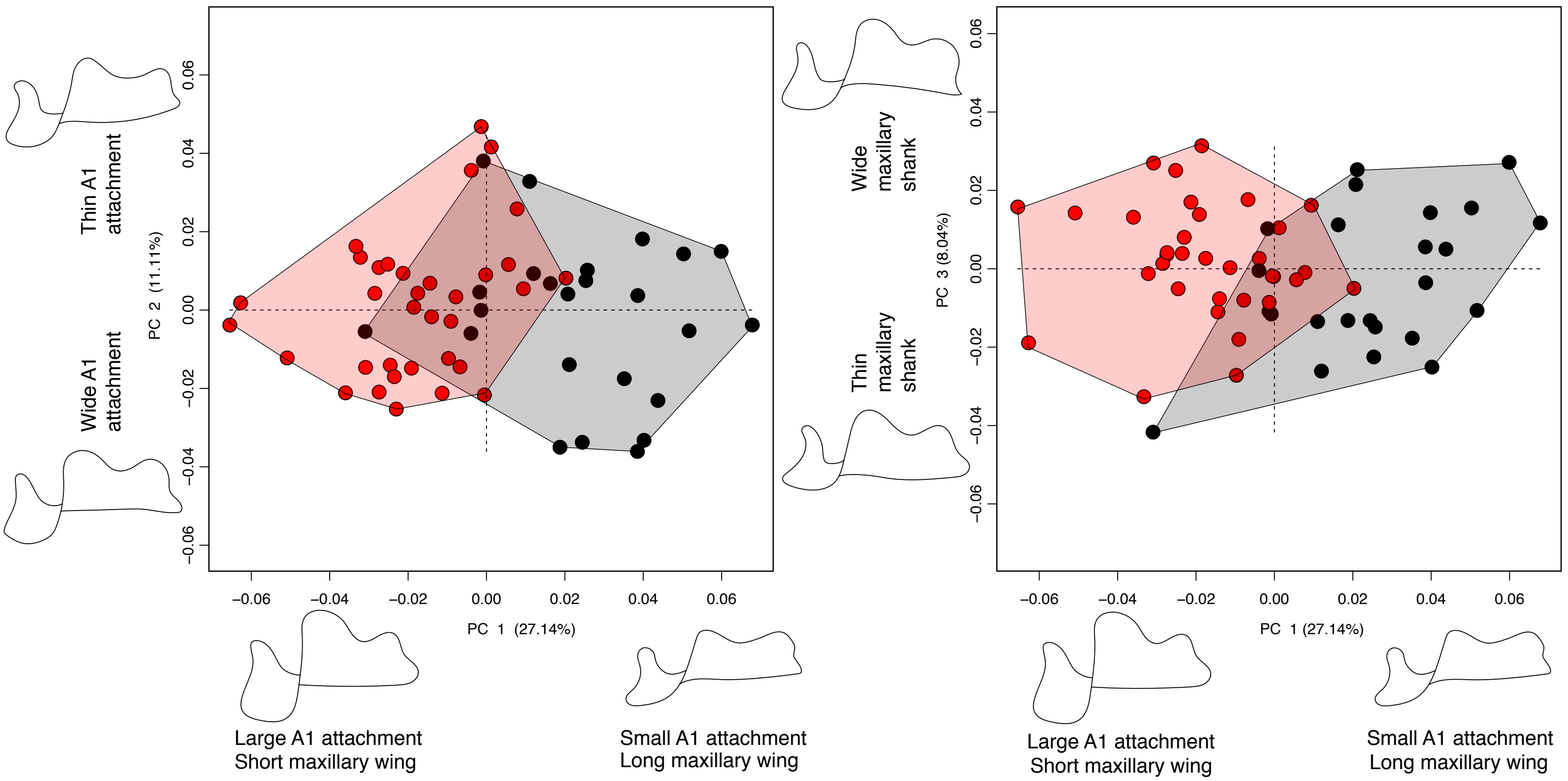

b, Pre-maxilla - Individuals

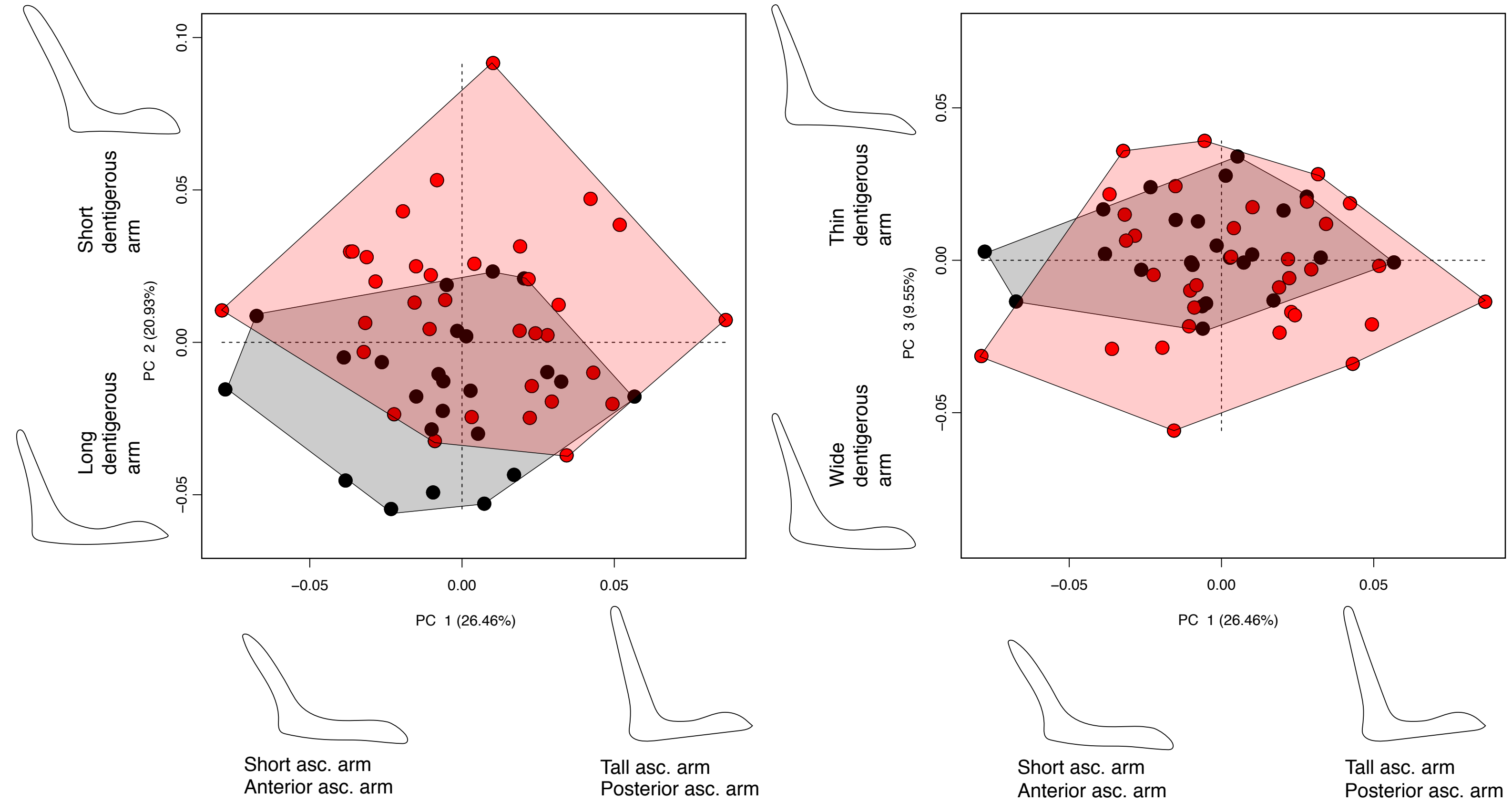

Figure S4. Schematics of Competing Modularity Models

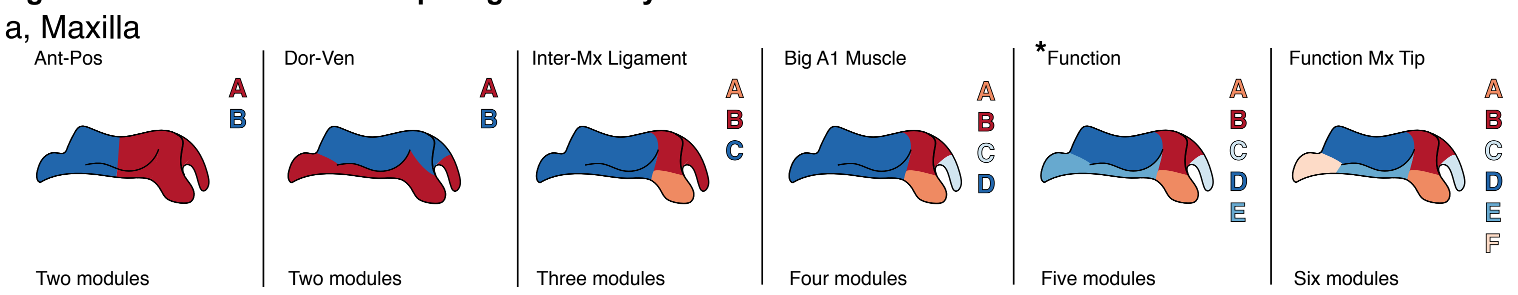

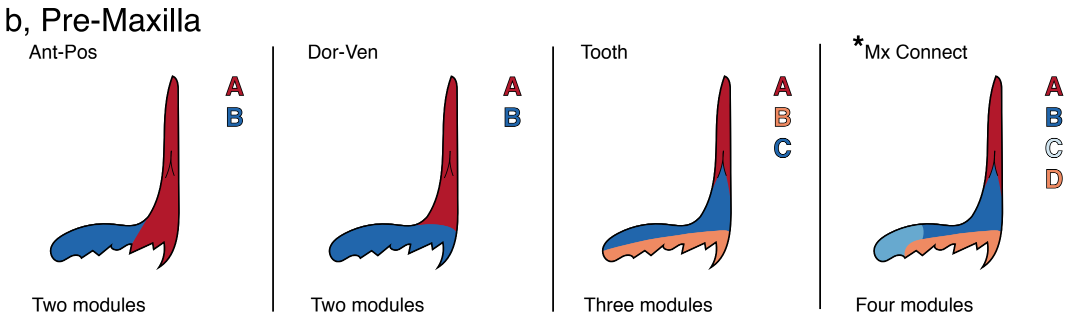

Figure S5. Maxilla

a, Rates of morphological evolution

b, Disparity

c, Integration

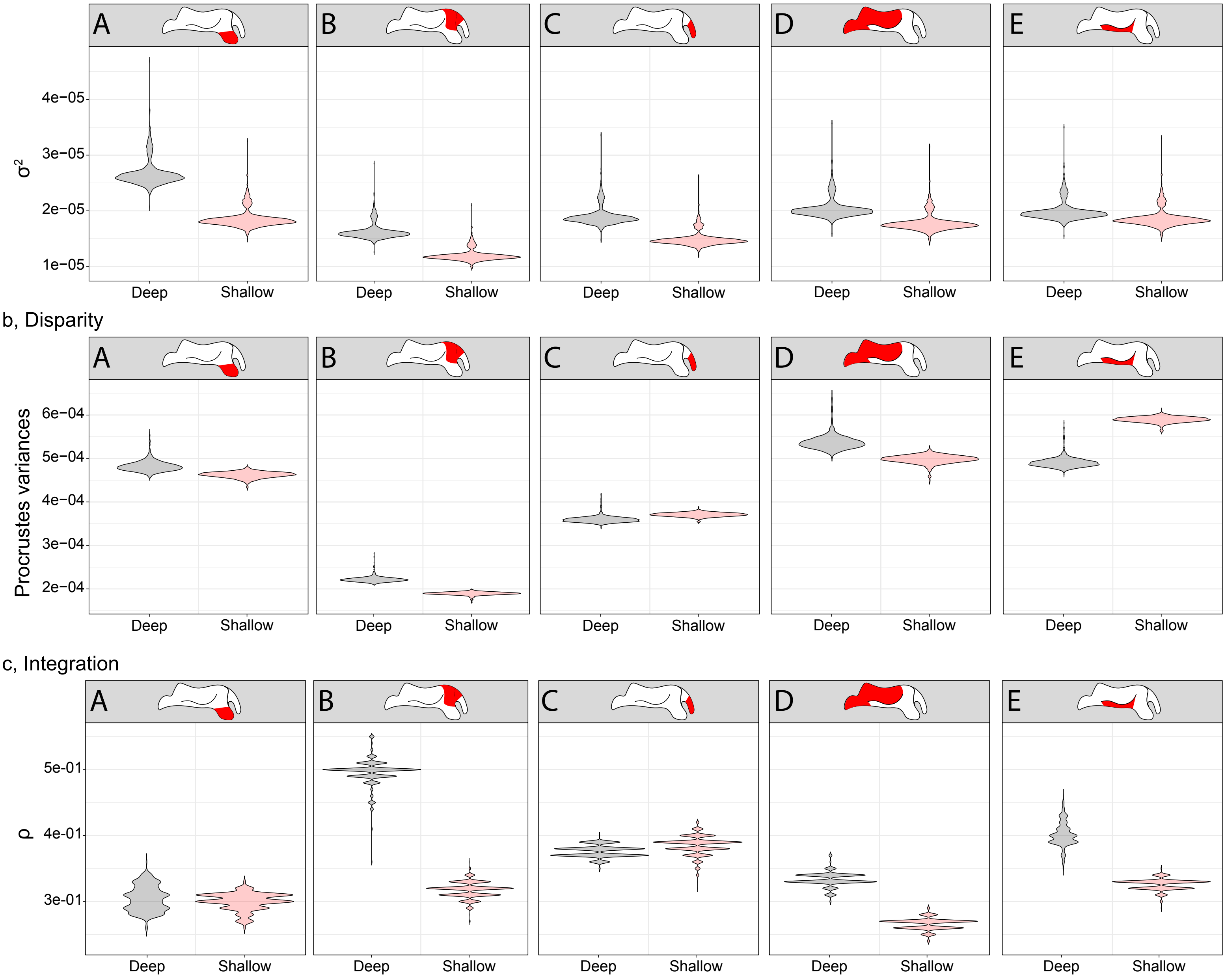

Figure S5. Pre-maxilla

d, Rates of morphological evolution

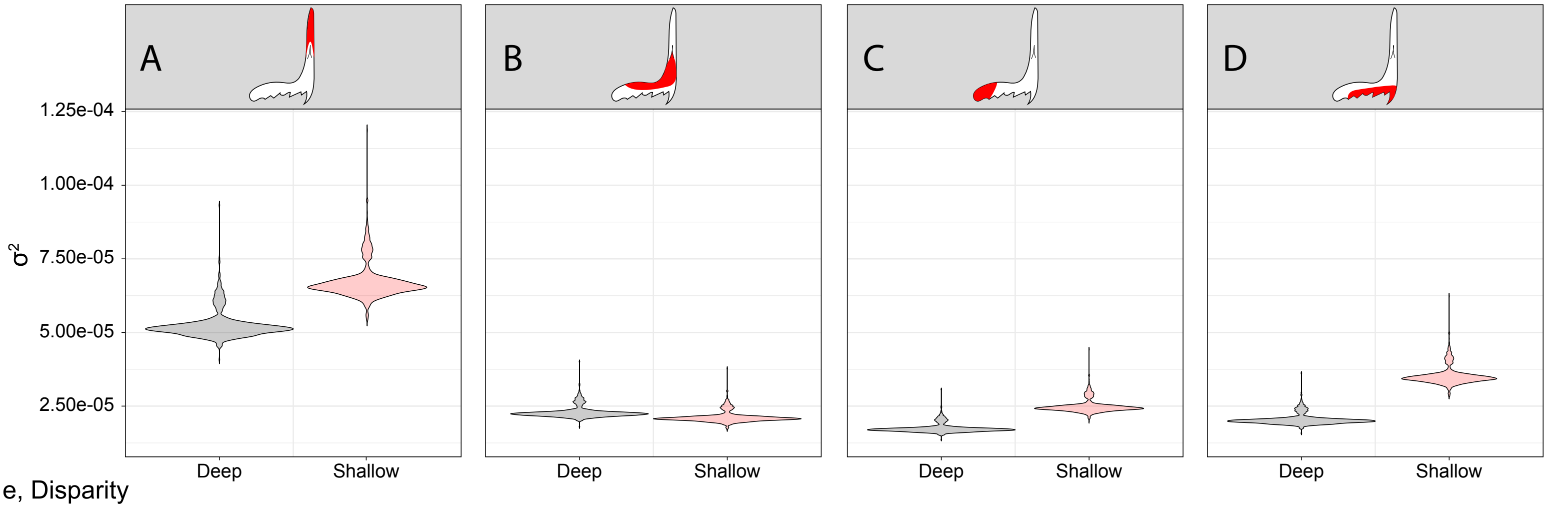

e, Disparity

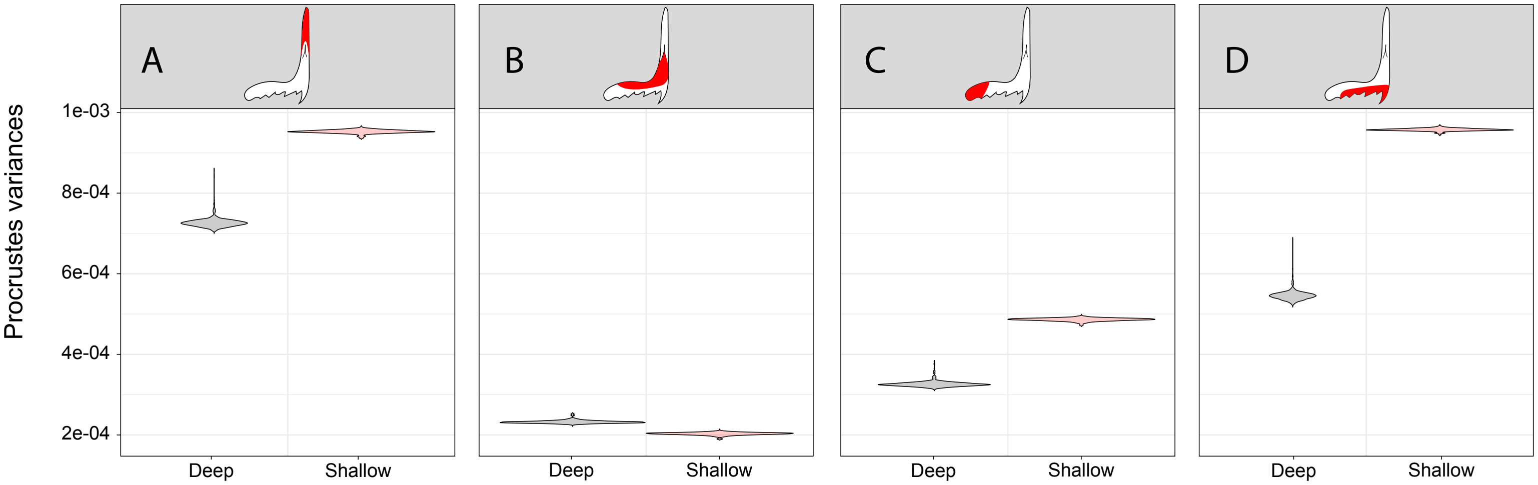

f, Integration

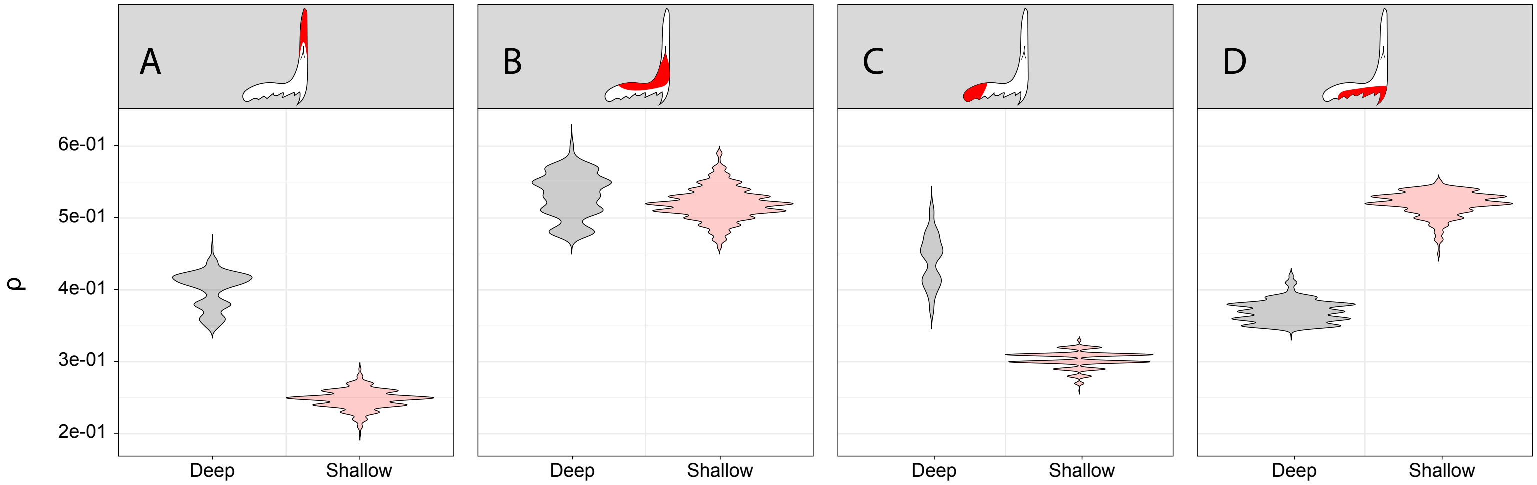

Figure S6. Selection Frequency of Competing Modularity Models

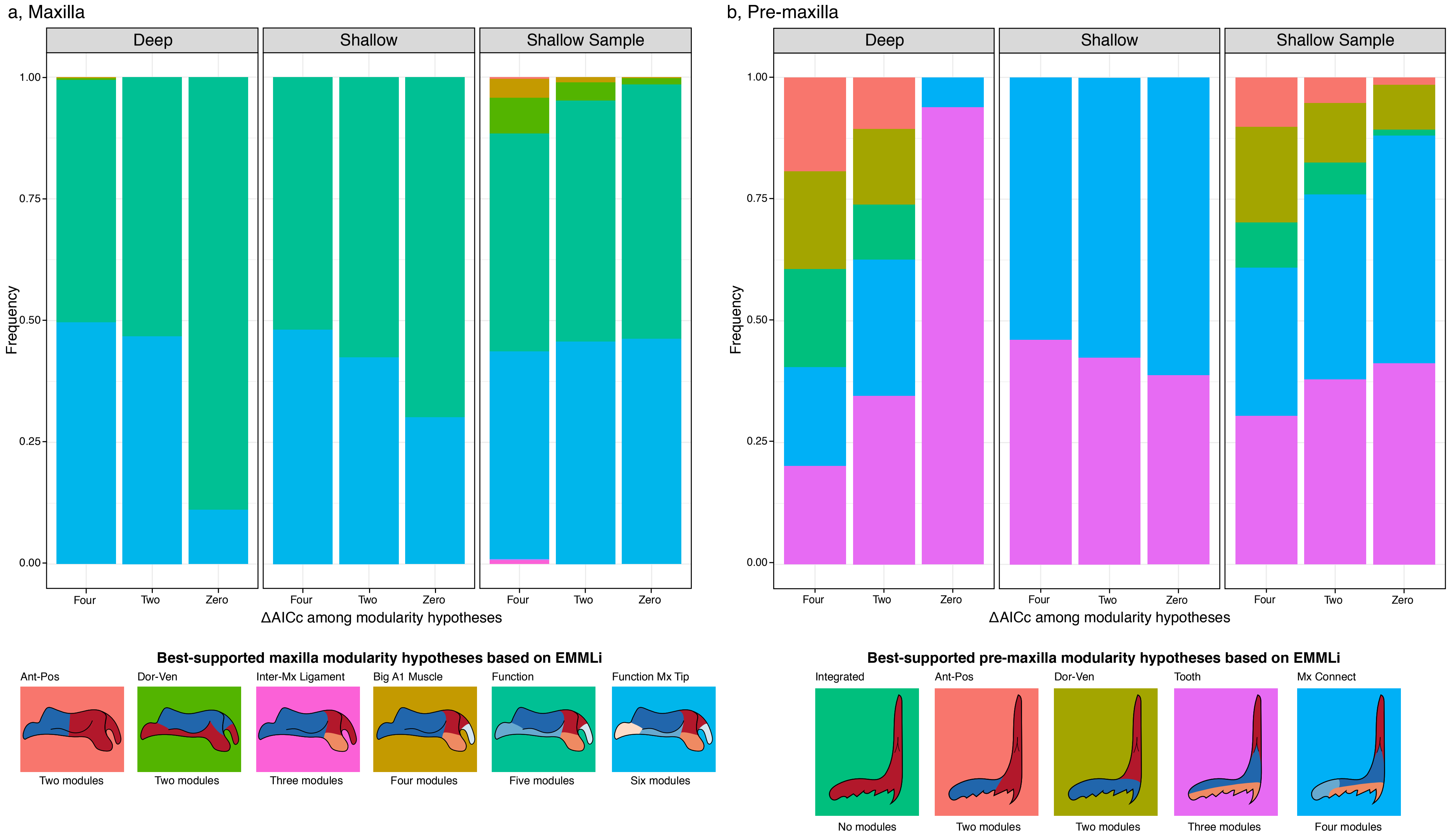

Figure S7. Experimental Morphospaces

a, Maxilla - Experimental

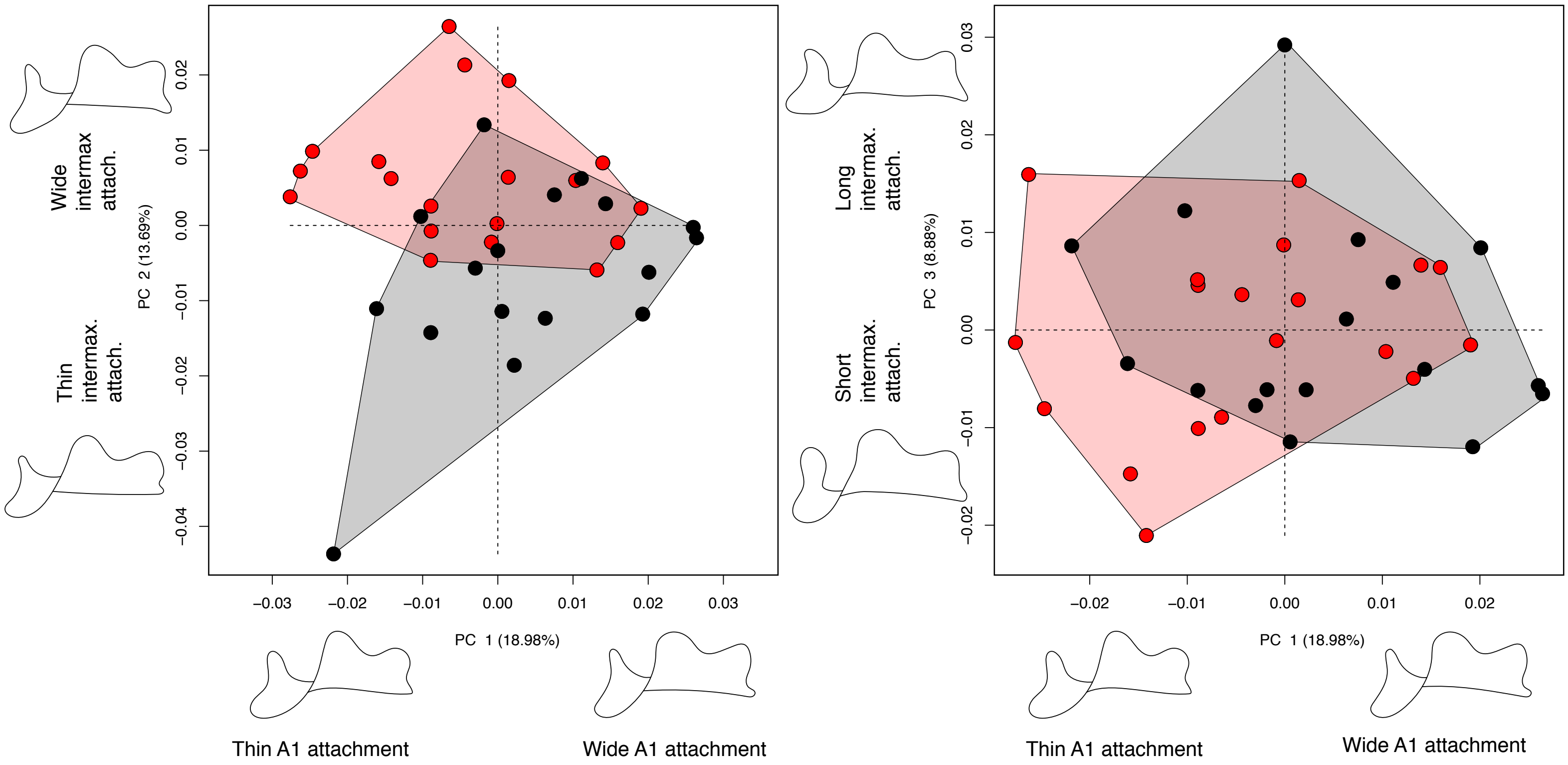

b, Pre-maxilla - Experimental

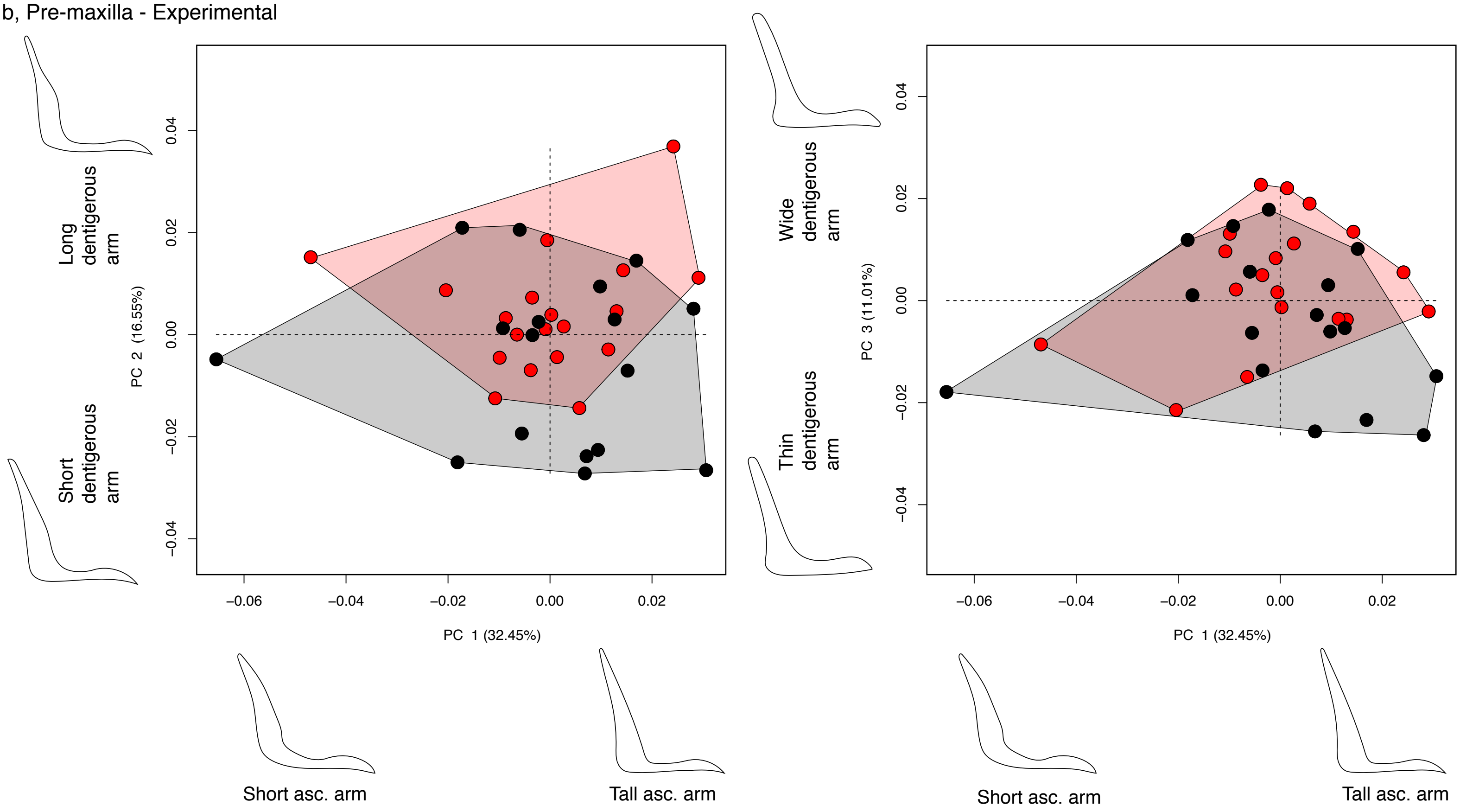

Supplement: Supplementary file 1 — Additional file 1. [file 12862_2020_1648_MOESM1_ESM.pdf]
