# Supplementary material for: Ecomorphological divergence and habitat lability in the context of robust patterns of modularity in the cichlid feeding apparatus
Source: BMC Evol Biol. 2020 Jul 31;20:95. doi: 10.1186/s12862-020-01648-x (PMC7393717; doi:10.1186/s12862-020-01648-x)
Supplement: Supplementary file 3 — Additional file 3. [file 12862_2020_1648_MOESM3_ESM.docx]

Supplementary Information for

Ecomorphological divergence and habitat lability in the context of robust patterns of modularity in the cichlid feeding apparatus

Andrew J. Conith^1^, Michael R. Kidd^2^, Thomas D. Kocher^3^, R. Craig Albertson^1^

^1^ Biology Department, University of Massachusetts Amherst, Amherst, MA, 01003

^2^ Department of Biology & Chemistry, Texas A&M International University, Laredo, TX, 78041

^3^ Department of Biology, University of Maryland, College Park, MD, 20742

**Contents**

**Part I. Natural Populations**

**Part II. Experimental Populations (Plasticity)**

**Part III. Additional Citations**

**Part IV. Supplementary Figure Legends**

**Part V. Supplementary Data and Trees**

All methods, results, and discussion concerning the upper oral-jaw bones, the pre-maxilla and maxilla, are restricted to the supplement. Broadly speaking, many of the major trends present in the mandible and lower pharyngeal jaw are replicated in the upper oral jaw (i.e., conservation of module patterns across habitats, similarity in disparity and rates across habitats, functionally salient modules exhibiting the fastest rates of morphological evolution).

**Part I.**

**Natural Populations**

*Morphological analysis*

We found a significant effect of allometry for the pre-maxilla (r^2^ = 0.109; P<0.001), but not the maxilla (maxilla: r^2^ = 0.020; P=0.290). See Figure S2 for maxilla and pre-maxilla landmark configurations, and Table S2 for all principal component scores.

*Maxilla*

The first PC axis for the maxilla characterized differences in the size of the intermaxillary ligament attachment site, the height of the adductor mandibulae 1 (A1) attachment site, and the length of the maxillary wing (27.14% of the variation). PC2 represented differences in the width of the A1 attachment site, and a ventral to dorsal project of the tip of the maxillary shank (11.11% of the variation). PC3 reflected change in the curvature of the maxillary shank base, the length of the maxillary shank, and the thickness of the maxillary body (8.04% of the variation). Maxilla morphology differed based on depth regime (pMANOVA; F=14.2, *P*<0.01), although most of this separation was confined to PC1 (Figure S3a; Table S3).

*Pre-maxilla*

Pre-maxilla PC1 characterized change in the height of the ascending arm, anterior-posterior projection of the ascending arm, and the thickness of the dentigerous arm (26.46% of the variation). PC2 explained differences in the length of the dentigerous arm (20.93% of the variation). PC3 reflected change in the width of the pre-maxilla (9.55% of the variation). Pre-maxilla morphology differed depending on occupation of deep or shallow habitats (pMANOVA; F=9.54, *P*<0.01), separation was primarily determined by PC2 (Figure S3b; Table S3).

*EMMLi Maxilla*

We found the best-supported module hypothesis for the maxilla partitioned variation into five-modules (Table S4, S5, S10; Figure S4a). A wealth of ligaments, muscles, and bony contacts are present across the maxilla, and this five-module ‘functional’ hypothesis attempts to capture all these disparate soft- and hard-tissue connections. These modules can be summarized as the dorsal shank (‘D’), ventral shank (‘E’), palatinad wing (‘A’), premaxillad wing (‘C’), and the neurocraniad process (‘B’) (See Conith et al. 2019 and Barel et al. 1976 for a more detailed break-down of cichlid craniofacial anatomy). Values of within-module integration (ρ) in the maxilla were found to be different between deep and shallow populations in three of the five modules: module B, the neurocraniad process (encompasses the subcondylar canal for a nerve); module D, the dorsal shank (forms the attachment site for the A1 muscle); and E, the ventral shank (forms a connection with the mandible). In all cases shallow populations exhibited the lowest overall ρ value and deep populations exhibited the greatest ρ values (Table S6, Figure S5c).

A six-module model (Figure S4a, ‘Function Mx Tip’) was also well supported, exhibiting AICc scores within two units of the five-module model (Table S5, S10). The six-module model splits the ventral shank into posterior and anterior regions, with the posterior region reflecting the connection between the maxilla and the mandible. The five-module model was selected at a slightly high frequency (50%-70%) relative to the six-module model, and these results are consistent across depths (Figure S6a). This result does not appear to be too susceptible to sample size differences, as alternate models are selected, on average, 10% of the time.

*EMMLi Pre-maxilla*

The best-fitting modularity hypotheses suggested by EMMLi typically identified four modules in the pre-maxilla (Table S4, S5, S10; Figure S4b). For deep members, EMMLi could not recover overwhelming support for a single hypothesis. While the ‘Tooth’ and ‘MxConnect’ hypotheses were within two AICc units of each other, all other models, including a model that allowed the pre-maxilla to be completely integrated, received AICc support within four units. For shallow members, EMMLi again could not recover support for a single hypothesis, with similar AICc support for the ‘Tooth’ and ‘MxConnect’ partitions (Table S5, S10), although the ‘MxConnect’ partition is typically selected more frequently (Figure S6b). ‘MxConnect’ partitions the pre-maxilla into four functionally important modules: the ascending arm (‘A’), tooth bearing region (‘D’), central portion of the premaxilla (including the medial region of the dentigerous arm, and the maxillad spine (‘B’)), and the posterior portion of the dentigerous arm that abuts the maxilla (‘C’). For two out of these four modules, deep members exhibited higher levels of within-module integration (Table S6; Figure S5f). Shallow members exhibited higher levels of within-module integration in the posterior portion of the dentigerous arm (‘C’) and the ascending arm of the pre-maxilla (‘A’). In contrast, shallow members exhibited higher levels of integration in the tooth-bearing region of the pre-maxilla (‘D’). We observed the highest overall levels of integration in the central portion of the dentigerous arm, which may result from this region being the point of articulation with the maxilla, so integration may be higher to facilitate this functional integration (Figure S5f).

In contrast to other bones we analyzed, the pre-maxilla appeared particularly susceptible to sample size differences, as many alternate models are selected in a reduced ‘Shallow’ sample (Figure S6b).

*Disparity and rates of morphological evolution*

We found no difference in disparity for any feeding bones between *Tropheops* from shallow versus deep environments, and this trend holds even when we compare the disparity of each of the five modules between depths (Table S6). Similarly, we found no differences in the rate of morphological evolution between deep and shallow environments. Finally, we also noted little difference in disparity or the rate of morphological evolution between depths when each bone was divided into modules based on the output provided by EMMLi (Table S6). While on average evolutionary rates were higher in deep-water populations for the maxilla and shallow-water populations for the premaxilla, this was only significant for the tooth-plate module (module ‘D’) of the pre-maxilla (σ^2^ deep = 2.04x10^-5^ (95% CI = 1.84x10^-5^, 2.52x10^-5^), σ^2^ shallow = 3.52x10^-5^ (95% CI = 3.17x10^-5^, 4.33x10^-5^), P=0.028). Taken together, results from both our disparity and rates analyses suggest that evolvability of the feeding apparatus is fairly similar across foraging habitats.

We found a few significant differences in the rate of morphological evolution among modules in the maxilla and pre-maxilla (Table S6). For example, the palatinad wing of the maxilla (the insertion point of the intermaxillary ligament, module ‘A’; Figure S4a) is evolving more than 1.5x faster than the neurocraniad process module (‘B’), which includes the subcondylar canal. Rapid evolution of the palatinad wing of the maxilla is notable from a functional perspective, as this bony element is the point of articulation for both the premaxilla (rostrally) and the palatine (caudally); thus, it likely plays a critical role in functionally integrating the upper jaw during feeding. In the pre-maxilla, the spine of the ascending arm (Figure S4b, module ‘A’) is the most rapidly evolving region. This too is notable as the length of this process is a critical determinant of jaw protrusion.

**Part II.**

***Experimental Populations (Plasticity)***

*Morphological analysis*

We found a significant effect of allometry on our shape data in the experimental population (maxilla: r^2^ = 0.134; P<0.001; pre-maxilla: r^2^ = 0.203; P<0.001). Overall, we observed less separation between treatments than in the natural populations (Figure S7; Table S8).

*Maxilla*

The first PC axis for the maxilla characterized differences in the width of the A1 attachment site on the dorsal wing, and a ventral to dorsal projection of the tip of the maxillary shank process (18.98% of the variation). PC2 represented differences in the length of the maxillary shank process, and the width of the intermaxillary ligament attachment site (13.69% of the variation). PC3 reflected change in the length of the intermaxillary ligament attachment site (8.88% of the variation). Maxilla morphology differed between those raised in benthic-shallow and limnetic-deep environments (MANOVA; F=6.32, *P*=0.007), although most of this separation was confined to PC2 (Figure S7a; Table S3).

*Pre-maxilla*

Pre-maxilla PC1 characterized change in the height of the ascending arm (25.83% of the variation). PC2 explained differences in the length of the dentigerous arm and the angle of the ascending arm (15.55% of the variation). PC3 reflected change in the depth of the dentigerous arm (13.02% of the variation). Pre-maxilla morphology differed depending on if they were raised in a benthic-shallow or limnetic-deep feeding environment (MANOVA; F=3.34, *P*=0.016; Figure S7b; Table S3).

*Experimental EMMLi Maxilla*

In contrast to the other bones we measured, the maxilla exhibits differences in the pattern of modularity between deep and shallow members (Table S9, S10). We found the benthic-shallow members best fit a hypothesis that partitions the maxilla into five modules (posterior probability (PP)=75.95%) that mimics the modularity hypothesis observed in the natural populations (see the *EMMLi Maxilla* section above). However, we found the limnetic-deep members best fit a hypothesis that partitions the maxilla into six modules (PP=94.33%). This hypothesis splits the maxillary shank module, observed in the benthic-shallow members, into two modules (Figure S4a). Specifically, EMMLi finds support for an extra module that connects the maxilla to the mandible. Values of within-module integration (ρ) in the maxilla were higher in the limnetic-deep population in 3/5 instances (Table S11). Notably, differences in ρ for the neurocraniad process (module ‘B’) were highly similar in experimental and natural populations, with the highest overall ρ value observed for the limnetic-deep treatment and one of the lowest values observed in the benthic-shallow treatment (Table S6, S11). This pattern suggests that plasticity may underlie trends among natural populations.

*Experimental EMMLi Pre-maxilla*

We found the best fitting modularity hypothesis for the experimental populations matched the four-module pattern observed in the natural population (see the *EMMLi Pre-Maxilla* section above). Both the benthic-shallow members (PP=32.48%) and limnetic-deep members (PP=63.15%) best fit a four-module hypothesis (Table S9, S10). The benthic-shallow members have all four ‘MxConnect’ hypotheses fitting within ~1 AICc unit of each other with the main differences between them coming from whether ρ is allowed to vary within and among modules. Little differences were noted for within-module integration between treatments. Similar to the natural population, the greatest levels of within-module integration were observed in the central portion of the premaxilla (module ‘B’), whereas the lowest levels were documented for the posterior portion of the dentigerous arm (module ‘D’) (Table S11).

*Morphological disparity*

We found no difference in the overall disparity for either upper jaw bone between *Tropheops* raised in the benthic-shallow versus limnetic-deep environments (Table S11). A similar trend was observed when disparity was compared within modules. The only exception to this was the ascending arm of the pre-maxilla (module ‘A’), where animals reared in the limnetic-deep treatment exhibited significantly higher levels of disparity (Table S11). Changes in the height of the ascending arm has large implications for suction feeding performance, so it is perhaps unsurprising that we observe significant plastic differences between treatments in this structure.

**Part III.**

**Additional Citations**

Conith, A. J., D. T. Lam, and R. C. Albertson. 2019. Muscle-induced loading as an important source of variation in craniofacial skeletal shape. genesis 57:e23263.

Barel, C. D. N., F. Witte, and M. J. P. Van Oijen. 1976. The shape of the skeletal elements in the head of a generalized *Haplochromis* species: *H. elegans* trewavas 1933 (Pisces, Cichlidae). Netherlands J. Zool. 26:163–265.

**Part IV.**

**Supplementary Figure Legends**

Figure S1. Phylogenetic tree of *Tropheops* and other closely related cichlid taxa from Lake Malawi. Four topology constraint positions are illustrated by red nodes. Node color is determined by posterior probability. See Table S1 for name translations.

Figure S2. Landmarking scheme for *Tropheops* maxilla and pre-maxilla. a, maxilla lateral view; b, maxilla ventral view; c, pre-maxilla anterior view; d, pre-maxilla lateral view. Red circles, fixed landmarks; blue landmarks, semi-landmark curve positions.

Figure S3. Morphospace occupation for natural *Tropheops* feeding bones from different depth regimes. a, Maxilla PC1-3; b, pre-maxilla PC1-3. Red, shallow members; black, deep members.

Figure S4. Partitioning schematics for competing modularity hypotheses. Colors reflect module partitions to be assessed by EMMLi. Letters correspond to the partitioning scheme (Table S4). The best fitting modularity hypothesis is illustrated by *. a, maxilla; b, pre-maxilla.

Figure 5. Violin plots depicting parameter values output from the rate of morphological evolution, disparity, and integration analyses across habitats for each module. Anatomical schematics illustrate the location of the module being tested in red. The depicted modules, and their associated letters, are based on the best-fitting modularity hypothesis determined by EMMLi. See Figure S4 and Table S4 for full range of modularity hypotheses we tested. a-c, maxilla; d-f pre-maxilla. Red, shallow habitat; Black, deep habitat.

Figure 6. Support for competing modularity hypotheses between habitats as determined by EMMLi. a, maxilla; b, pre-maxilla. The ‘Shallow Sampling’ plot reflects the frequency of module model selection derived from a sample size that matches the deep population. Colors used in the module model frequency plots are placed as background colors on module partition schematics that they represent.

Figure S7. Morphospace occupation for experimental *Tropheops* feeding bones from different depth regimes. a, Maxilla PC1-3; b, pre-maxilla PC1-3. Red, benthic-shallow members; black, limnetic-deep members.

**PART V.**

**Supplementary Data and Trees**

**A, Ultrametric ‘species’ tree – pages 9-10**

(Rhamphochromis_sp.:1.692615442,((Copadichromis_borleyi:0.6433309806,((Lethrinops_auritus:0.5411157818,Mylochromis_mola:0.5411157818):0.0417524597,(Lethrinops_lethrinus:0.4892527216,Protomelas_taeniolatus:0.4892527216):0.09361551984):0.06046273908):0.1373125293,(((Petrotilapia_sp.:0.4676946627,(Melanochromis_auratus:0.08351454336,Cynotilapia_afra:0.08351454336):0.3841801193):0.2453996348,((Maylandia_aurora:0.5389860949,Maylandia_barlowi:0.5389860949):0.04174844667,(Maylandia_benetos:0.5496374552,(Maylandia_zebra-DI:0.4632293378,(Maylandia_zebra-MU:0.4140915459,Maylandia_zebra-MZ:0.4140915459):0.04913779194):0.08640811733):0.03109708639):0.1323597559):0.02967175458,((Tropheops_gracilior-DI:0.3267056527,(Tropheops_gracilior-OP:0.2968460334,Tropheops_otter_gold-OI:0.2968460334):0.02985961928):0.06869011836,((Tropheops_bb-MZ:0.311031835,Tropheops_gold-ER:0.311031835):0.02674665687,((Tropheops_white_dorsal-NM:0.2809174147,(Tropheops_black_dorsal-CH:0.2604637526,Tropheops_black_dorsal-NM:0.2604637526):0.02045366211):0.02011141177,((Tropheops_black_dorsal-WR:0.2495532674,(Tropheops_broadmouth-OI:0.2406566589,Tropheops_bz_MZ:0.2406566589):0.008896608515):0.02277673737,(((Tropheops_orange_chest-ER:0.224908799,(Tropheops_chinyankwazi-CK:0.1984591757,Tropheops_chinyamwezi-CW:0.1984591757):0.02644962326):0.007209711054,(Tropheops_gold_chest-CH:0.2159717524,(Tropheops_lilac-TW:0.2066550465,(Tropheops_mk-MK:0.1992798495,(Tropheops_nf-OI:0.1927184117,(Tropheops_zimbawe-ZR:0.173800739,(Tropheops_lilac-MZ:0.1596090676,(Tropheops_mu-MU:0.1386693515,Tropheops_zebra-MU:0.1386693515):0.02093971606):0.01419167146):0.0189176727):0.006561437808):0.007375196951):0.009316705881):0.01614675767):0.01771281064,((Tropheops_lilac-OP:0.2141164297,(Tropheops_orange_chest-OI:0.1996444978,Tropheops_orange_chest-TW:0.1996444978):0.01447193189):0.0213600047,((Tropheops_lilac-MU:0.21945655,(Tropheops_intermediate-OI:0.2003748895,Tropheops_intermediate-TW:0.2003748895):0.01908166056):0.007590074967,((Tropheops_black_dorsal-MU:0.2014040499,Tropheops_intermediate-MZ:0.2014040499):0.01514513377,(Tropheops_red_cheek-TW:0.2014516921,(Tropheops_microstoma-DI:0.1655049839,Tropheops_microstoma-OP:0.1655049839):0.03594670815):0.01509749158):0.01049744131):0.008429809441):0.01435488624):0.02249868413):0.02869882163):0.03674966548):0.05761727917):0.347370281):0.03787745781):0.9119719317);

**B, AFLP full tree – pages 11-12**

(aTrduNA000001:0.07705392,aTrduNA000002:0.1197094,(aRhspNA011857:0.05658028,(aRhspNA011858:0.07298015,(aRhspNA011854:0.1080443,((aRhspRP011854:0.2034409,(aRhspRP011857:0.1580699,aRhspRP011858:0.1341926):0.05197207):0.04599811,(((aCoboOP011819:0.07179698,(aCoboOP011820:0.07071964,aCoboOP011834:0.07488981):0.02263126):0.05046238,(((aLedeCB011902:0.1226037,(aLedeCB011900:0.09243236,aLedeCB011901:0.1473722):0.02892942):0.01854013,(aMamoOC011836:0.06371877,(aMamoOC011837:0.09949134,aMamoOC011948:0.04673039):0.02051193):0.08043447):0.01315991,((aLeleCB010205:0.05583513,(aLeleCB010206:0.1937898,aLeleCB011917:0.08753202):0.03372556):0.06714597,(aPrrdOC013134:0.06700861,(aPrrdOC013131:0.06475211,aPrrdOC013132:0.06387624):0.01752458):0.04677899):0.03045814):0.01935176):0.03793258,(((aTrbdER0100369:0.1490601,(aTrbdER0100371:0.3432063,(aMelaurTWI01069:0.01539498,(aMelaurTWI01070:0.04414334,(aCyaafrCH010246:0.03075097,(aCyaafrCH010245:0.03550618,aCyaafrCH010681:0.05122556):0.01034813):0.03845357):0.0119315):0.3167706):0.124342):0.09266753,(((aMeauOP010027:0.09460481,(aMeauOP010025:0.1027227,aMeauOP010026:0.0727825):0.01822189):0.03351119,(aMebaMZ011593:0.1379475,(aMebaMZ011591:0.09672284,aMebaMZ011592:0.06989394):0.01141902):0.04943759):0.01185573,((aMebeMZ011604:0.08420255,(aMebeMZ011603:0.1027034,aMebeMZ011605:0.116168):0.02571809):0.06453229,((aMezeDI011411:0.09074106,(aMezeDI011410:0.08784426,aMezeDI011412:0.1015995):0.01573746):0.042156,((aMezeMU011548:0.1032347,aMezeMU011549:0.07092849):0.03075411,(aMezeMZ011614:0.2090573,(aMezeMU011550:0.0942628,(aMezeMZ011615:0.08902219,aMezeMZ011618:0.1071852):0.03318045):0.01791806):0.0372306):0.01852023):0.02932637):0.01091477):0.04076811):0.01935719,(((aTrgrDI011384:0.0882702,(aTrgrDI011385:0.09184367,aTrgrDI011386:0.09359333):0.01198825):0.01896991,((aTrgrOP010131:0.07429809,(aTrgrOP010310:0.0873186,aTrgrOP010323:0.08938586):0.01598226):0.013949,(aTrogOI010317:0.07741476,(aTrogOI010103:0.08807766,aTrogOI010318:0.07113011):0.01220914):0.02053853):0.01236359):0.02798931,(((aTrbbMZ011595:0.0694551,(aTrbbMZ011594:0.08740989,aTrbbMZ011596:0.06501776):0.01384824):0.04472431,(aTregER010373:0.05430319,(aTregER010372:0.0634185,aTregER010374:0.08345018):0.00742305):0.05283189):0.01287435,(((aTrwdNM010398:0.08716881,(aTrwdNM010399:0.06769517,aTrwdNM010400:0.08913306):0.009778096):0.04972377,((aTrbdCH010391:0.07953954,aTrbdCH010392:0.1007623,aTrbdCH010393:0.08448937):0.01744677,(aTrbdNM010795:0.09366604,(aTrbdNM010396:0.1011726,aTrbdNM010397:0.08381921):0.007411805):0.01847437):0.00758561):0.008269002,(((aTrbdWR011004:0.09178725,(aTrbdWR011005:0.06957946,aTrbdWR011006:0.08724818):0.01664069):0.02080219,((aTrbmOI010106:0.09022121,(aTrbmOI010107:0.08144526,aTrbmOI010108:0.09551717):0.01960616):0.02994576,(aTrbzMZ011597:0.08743446,(aTrbzMZ011598:0.1035365,aTrbzMZ011599:0.06651055):0.01409421):0.02059565):0.00535473):0.01248577,((((aTrocER010376:0.07338127,(aTrocER010375:0.07033402,aTrocER010377:0.09647638):0.01170173):0.0433092,((aTrckCK010351:0.08004453,(aTrckCK010349:0.09284882,aTrckCK010350:0.06232681):0.01850173):0.0430602,(aTrcwCW010326:0.1272478,(aTrcwCW010327:0.09208471,aTrcwCW010328:0.07171923):0.01360789):0.02201003):0.01655097):0.003771758,((aTrgcCH010381:0.08209994,(aTrgcCH010382:0.1320873,aTrgcCH010383:0.08759139):0.01366457):0.04489229,((aTrliTW010159:0.1059746,aTrmiDI011382:0.1227341):0.01095643,((aTrmkMK010894:0.07946288,(aTrmkMK010892:0.06560395,aTrmkMK010893:0.06856153):0.0175595):0.03899038,((aTrnfOI010324:0.1215042,aTrnfOI010325:0.08856102):0.04474435,(aTrliTw011790:0.1914727,(aTrziZR011115:0.1816676,(aTrliMZ011656:0.1725369,(aTrmuMU011756:0.1134399,(aTrmuMU011755:0.1147671,(aTrmzMU011753:0.1405443,aTrmzMU011754:0.1107001):0.03694989):0.01398059):0.02160084):0.01644095):0.01245056):0.01149816):0.007128576):0.006701541):0.006800268):0.01119076):0.00970787,(((aTrliOP010151:0.1403469,(aTrliOP010152:0.08803968,aTrliOP010153:0.06464805):0.01195891):0.0304451,((aTrocOI010319:0.09632062,(aTrocOI010320:0.08124667,aTrocOI010321:0.09824589):0.007879094):0.0178741,(aTrocTW010303:0.1001694,(aTrocTW010304:0.08926141,aTrocTW010305:0.07421564):0.0245404):0.01794193):0.008485164):0.01038444,(((aTrliMU011499:0.06728833,(aTrliMU011500:0.1286647,aTrliMU011501:0.07584884):0.01331843):0.04195734,((aTrinOI010302:0.08346053,(aTrinOI010197:0.07971994,aTrinOI010301:0.08143724):0.009881737):0.02232965,(aTrinTW010174:0.0960653,(aTrinTW010175:0.07719206,aTrinTW010176:0.07747472):0.0167242):0.0309613):0.01032363):0.005263826,(((aTrbdMU011494:0.08631041,(aTrbdMU011495:0.06562531,aTrbdMU011496:0.06974056):0.01265526):0.05305705,(aTrinMZ011645:0.06878023,(aTrinMZ011646:0.0896597,aTrinMZ011647:0.07564487):0.009938447):0.04003973):0.008323891,((aTrrcTW010162:0.06726509,(aTrrcTW010160:0.07252459,aTrrcTW010161:0.07046107):0.01706241):0.03669708,(aTrmiDI011379:0.1286261,(aTrmiOP010322:0.1183192,(aTrmiOP010313:0.2877228,aTrmiOP010314:0.07423265):0.02085225):0.01883808):0.02710909):0.01025976):0.005865783):0.00483742):0.006045315):0.00707554):0.007400287):0.005347886):0.008429047):0.04322004):0.01305891):0.127063):0.1150611):0.01546899):0.05284179):0.4823206);

**C, Nexus file and associated MrBayes block for constructiung AFLP phylogenetic trees – pages 13-404**

#Nexus

begin data;

dimensions ntax=138 nchar=7953;

format datatype=restriction interleave=no gap=- missing=?;

matrix

aTrduNA000001 11011001110101010011010000001011111011101011110110001000100110001101011111001001001011000101001000001101001110101010001001010011101000100001100000001000000011010001001101000100101100010001000000010000000000000000000100100000000000101010000000000000110000000000000000000000000100000000010000000100001100010010000011000001000000000010000100100001001000001000000000000010000000000000000000000000010001000010000000101000000000010100000000000000000000000000000001000000000001010111110011100110101101101101011011110100110110010001001001101100100011010100110111110100000110100001010011010000000101000010101111100110100001010110001010101100011010000011000001101000100010011100101100001101111010010001000001000000011100000000110001100000100100001000110001000001110000000011100000000001000000000010100000010001010011000000100001010001000000010000000000000000000000000000000010001000011000000001000000000000000000000000100001000000000000000000000000000000010000000000000000000000000000000000000000000000000000000000001000000000000000000000000000000000000000000010000000000000000000000000000000000000000000000000000000000000000000000000000000000000000000000000000000000000000000000000000000000000000000000000000000000000000000000000000000000000000000000000000000000000000000000000000000000000010110100100001110101011110010001011100111011001000111011001000010100101001010001100010101001010100101011010100101000000010001000101010010010111010110100100101001100101000100101010001000010010000011100000100001000000101000000000100000011100000000000100011010100010000010001000000000000010000000000100000000000010000000010110001000000000000000000000000000000000000000000000000000010000000000000000001000000000000000000000000000000000000000000000000000000000000000000000000000000000010001001010000010100000010110011001011000101010011011010100101001000110100010001101111001100001001100010010000000100101010001000010011001001000000000000100000001000000110000101100000101000101101000000000000000010010000010100000001010000110000001000001000010000000100000100000100110000000000000000011000001000000000000000100110000010000100100010010001000000000100000000000000110000010000000000000010100000000000000000000000001??????????????????????????????????????????????????????????????????????????????????????????????????????????????????????????????????????????????????????????????????????????????????????????????????????????????????????????????????????????????????????????????????????????????????????????????????????????????????????????????????????????????????????????????????????????????????????????????????????????????????????????????????????????????????????????????????????????101111010111100101101000100111001001010110110001010100010010100111101101011001100001001000101000011000010011010010010001001011011100001010000010001000100000110000000001010000000001011000101000011100000000000000110001001010000100000001010000000100101000110001000000000010000010100000000100000010000000011000010000000000000000000000000000000000001000000000001000000100000000000000000000000001000100001010000000000000000000000000000000000001000000000011111100011011110111010101100111111010001110010110011101101101010101101101011001101110100011001101101000110010110110100000001010101001000100100000111001000010010000011101001110101010000001000001010000000101101011111101000000100010001000000000001000000001100000000000100000000100000000000000000100000000101000000000001000010011100000000100000000000000000010000000000000010000000000000000000000000000000000000010000000000000000000000000000000000000000000000000000000000000000010110101110111011000101111011000101100010101101101110101001011011110100101100110000111101010010010110100111001011101001010000111100011000001001101001001100110001100010101100010010011001000010000100000100101101010100110010010010000010000000000000000000000100000100100010010001001010001001000110001000000001000001000000000100010000000001000100000001000000101000000000000100100000000000000000000000000000000000000000000000000000???????????????????????????????????????????????????????????????????????????????????????????????????????????????????????????????????????????????????????????????????????????????????????????????????????????????????????????????????????????????????????????????????????????????????????????????????????????????????????????????????????????????????????????????????????????????????????????????????????????????????????????????????????????????1010110001100110001100011001000110100111000100111001111100110110001111011101000100101001100000100000111011000000000000000000101000001110000000011100010000010000011000000110000100000100000010000000000000000000010110001000000001000000000000000001000010000010000000000011000011010000000010001000000000000000100000000000000000000000001010000000000000100100000100000010000001000100001000000000000010100100010111001100110000010101001010010000001000001001000000100010110000010000011000000001000000000000000000000000101001001000000000000000001000000000000000010000010100000000000000000000100010000000000000001000000000100000000000101001000100100000000000000000000000000000000000000000000000000000000000000000000000000000001000000000000000000100000000000000000000000000000000000000000000000000000000000000000000000000000000000000000000000000000000000000000000000000000000000000000000010001000011110101010000010100011001000010001001011101011000100000000100001100110101001010001000001100010011100100101011001100100000000001000001000110001000001000011000100001001000000010000000000001000000000100000000000001000000100000000001000000000000100000001011000000000000001000000100000000000000000000000000001000100000000000000000010000000000010000001000000000000000000000010000000000000100000000000000000000000000000000000001000011010101110101011011001011001101001010011011100010101101001010010001101100001000000001010100100010001011110001000000000010010010110010100001000100000000000001100101001000000100000000000000011010000000100100000000000001000001000010000110000100100100000000000000000001000000100100010100001001000000110000001100000000000000010010000100000001000000000000000100000000000000000000000000000010000000000000000000000000000000000001001000000000000001000001000000000000000000000000000000000??????????????????????????????????????????????????????????????????????????????????????????????????????????????????????????????????????????????????????????????????????????????????????????????????????????????????????????????????????????????????????????????????????????????????????????????????????????????????????????????????????????????????????????????????????????????????????????????????????????????????????????????????????????????????????????????????????????????????????????????????????????????????????????????????????????????????????????????????????????????????????????????????????????????????????????????????????????????????????????????????????????????????????????????????????????????????????????????????????????????????????????????????????????????????????????????????????????????????????????????????????????????????????????????????????????????????????????????????????????????????????????????????????????????????????????????????????????????????????????????????????????????????????????????????????????????????????????????????????????????????????????????????????????????????????????????????????????????????????????????????????????????????????????????????????????????????????????????????????????????????????????????????????????????????????????????????????????????????????????????????????????????????????????????????????????????????????????????????????????????????????????????????????????????????????????????????????????????????????????????????????????????????????????????????????????????????????????????????????????????????????????????????????????????????????????????????????????????????????????????????????????????????????????????????????????????????????????????????????????????????????????????????????????????????????????????????????????????

aTrduNA000002 11011101110101010011010100001011111011101111010110001000110100001100011111001001001111000101101000001101010110101010001101010011101000100001000000001110000010001001101101000100101100010001000000010000000000000000010100100000000000101010000000000000110000000000000000000000000100000000010000000100001100010010000010100001000000000010000100100001001000001000000000000010000000000000000000000000010001000010000000101000000000010100000000000000000000000000000001000000000001010111010011100111011101101100011011111101110101010011011101100000100011010111111111110110000110100001010010010000001101100001101111100110100000000110001010100100010010000000000001111000100000001110101100001101111010011111010001000000011100000000110001010100100010000100100001000000000000000001000000000000000000000000010000000101000001000001000001000001000000010000000000000000000000000000000011001000001000000001000100000000000001000000000001000000000000000000000000000000010000000000000000000000001000000000000000000000000000000000001000000000000000000010000000000000000000000010000000000000000000000000000000000000000000000000000000000000000000000000000000000000000000000000000000000000000000000000000000000000000000000000000000000000000000000000000000000000000000000000000000000000000000000000000000000000010100101001001010101101110011000100100111011101000101011001000010100101001010001100011010010010000001010010010001000001100001000001000010000011001100000100101001100000000100001010001000011000000000100010100000001001000000000000100000010000000000000100011010100010000010000000001000000010000000000100000000000010000100010001001000000000000000100000000000000000000000000000000000010000000000000000001000000000000000000000000000000000000000000000000000000000000000010000000000000000001010011010010001101010010100011010111010101011001011101010101001010110101000101011111010100001001010010001000000100100011010000010001011001000001000000100000001000000101000101110000110110001011000001100101001010010100010100001010000001010000001000000000011000110000000000100100100000000000000000101000001000000000000000000100000000000100100000000001000000000100000000000000100000010000000001000010000000000100000000000000001??????????????????????????????????????????????????????????????????????????????????????????????????????????????????????????????????????????????????????????????????????????????????????????????????????????????????????????????????????????????????????????????????????????????????????????????????????????????????????????????????????????????????????????????????????????????????????????????????????????????????????????????????????????????????????????????????????????101111010101101010111000100110001001010110111001010100010010100111101101011001100000001000101000011100010011010010010001001010011100001010000010001000110000110000000000110000000001011000101000010100000000000000110001010001000100000001010000000100100000110001000000000010000010100000000100000010000000011010010000000000000000100000000000000000000100000000001000001000000000000010000000000001000100001010000000000000000000000000000000000001000000000011111100011011110111010101100010111110100000010110011101101100010101001101010001101100000011001100101000100010110110000000001000101000100100100000101001000010110000011101001101001010000001000001010000000101101011101101000000100010001000000000001000000000100000000000100000000100000000000000000100000000101000000000001000010010100100000100000000000000000010000000000000010000000000000000000000000000000000000010000000000000000000000000000000000000000000000000000000000000010010110101110111011000001111011000101000110101101101110001001010111110101101101111000111101100010010110110111000011101001010100110100011000001001001011001100110001100000101100010010011001000010000100000100100101010100110010010011000010000000000000000000000100010100100010100001001010001001000010010000000001000001000000000100010000000001000100000001000000101000000000000100100000000000000000000000000000000000000000000000000000???????????????????????????????????????????????????????????????????????????????????????????????????????????????????????????????????????????????????????????????????????????????????????????????????????????????????????????????????????????????????????????????????????????????????????????????????????????????????????????????????????????????????????????????????????????????????????????????????????????????????????????????????????????????1010110001100110001101010001000110100111000110110101011100110110001111011001000100101001100000100000111110100000000000000000101000001110000000010100010000010000011000000110000000000100000010000000000000000000000110001000000001000000000000000001000010000010000000000011000011010000000010000100000000000000100000000000000000000000001010000000000000100100000100000010000001000010001000000000000001000000001000000100000000010010010100001010001000000010000001000101000000000001000000000000001000001000000100000000000001010000100000000010000000000110000010100101011000000000100000100001000000010000000100000100001001011000100000010000100000000000000000100000000000100000101001000000000101010000000101001010110000000001100010000101001000011000000010010000000001000010000010011010110111110001010000100100000001001000000000000101001000010000101000110001001000000000100000011000100010010010010111110110110000010100111101100011101101010101111110101000010110001010110101011010101000011100010011010101101010001010100000011001100001100110010000011100011000100001011000000011001001010001000000001100000000100011000000100100000001000100000000101100101001000001010000011000010010000000000000100000000000001000100000000000001000010000000000010100001000010000000010010000010000000000000100000000000000000000000000000011010001000010010101100100011011001010000100010010011011000010101001001000001000101100101000010101011100110010001010110011001000000010011010000010110010010100010000000001100001001000000000000000000100001000000000100100010000000001000001000010001110000000000010100000100000100000000000100000110100000001000000100000010000000000000100010010000100000000010101000000001100000000000000000000000000000000100000001000100000000000000000100001001000000000000010000001000000000000000000000000000000000??????????????????????????????????????????????????????????????????????????????????????????????????????????????????????????????????????????????????????????????????????????????????????????????????????????????????????????????????????????????????????????????????????????????????????????????????????????????????????????????????????????????????????????????????????????????????????????????????????????????????????????????????????????????????????????????????????????????????????????????????????????????????????????????????????????????????????????????????????????????????????????????????????????????????????????????????????????????????????????????????????????????????????????????????????????????????????????????????????????????????????????????????????????????????????????????????????????????????????????????????????????????????????????????????????????????????????????????????????????????????????????????????????????????????????????????????????????????????????????????????????????????????????????????????????????????????????????????????????????????????????????????????????????????????????????????????????????????????????????????????????????????????????????????????????????????????????????????????????????????????????????????????????????????????????????????????????????????????????????????????????????????????????????????????????????????????????????????????????????????????????????????????????????????????????????????????????????????????????????????????????????????????????????????????????????????????????????????????????????????????????????????????????????????????????????????????????????????????????????????????????????????????????????????????????????????????????????????????????????????????????????????????????????????????????????????????????????????

aRhspNA011854 11011101110101010111011001000101101011001110010110110101100100111101101010101101001110000101101010001001001010101001101111010011101111001101110000010011000110001011010101011000100100000110000000000010000000000000001100000000000000011000000000100000101000000000001001100000010000010000010000000100001000100010000001010000000000001000000000000001001010000100100010000010000001000000000000001000100001000000100000000000000000000000000001000000000000000000000001000000100010010111100011100111001101111100010111010101110000110001001001100001100011010101110110110101000100111000111001010101010011010110101011000110000000001001011010000010010000010000000001000000000000111100000110000100011000010101000100000000111010000000000001010001000000000001100010000000000000010001000000000000000000000000100100000000000100000000000001000000000000000000000000000000000000000000010001001000000001000001000100000001000000000000010000000000000100000000000000000000000000000000000000000000001000000000000000000000000000000000000000000000000000000000000000100000000000000000000000000000000000000000000000000000000000000000000000000000000000000000000000000000000000000000000000000000000000000000000000000000000000000000000000000000000000000000000000000000000000000000000000000000000000000000000000000000010101000001000100101001100010000000010100111101000100110001100010100101001001001010100010000000000101010000000001000000010101000101010011000001010110000100101001000000111000000010000000000010000010100000100010000101000000000000000000000000000000000010010010001000000010010000100000000000000000000000000000000010000001000000100010000000000000000010000000000000000000000000000000000000010000000000001000000000000000000000000000000000000000000000000000000100000000000000000000000000000010101010100001000110010011011010101000100011010001001000100000100100011010100100100001000000001100011010000001000000000001000000010000000000000000000010000000000001000000000000000010000000000000000010000000000100000000110000000000000000000010000000000000000000000000000101000000000000000000000000000000000000000000000000000000000000000000000100000000000000000000001000000000000000000000000000000000100000000000100000000000??????????????????????????????????????????????????????????????????????????????????????????????????????????????????????????????????????????????????????????????????????????????????????????????????????????????????????????????????????????????????????????????????????????????????????????????????????????????????????????????????????????????????????????????????????????????????????????????????????????????????????????????????????????????????????????????????????????110110011101101110111010110110101111010110011101010000000000000000001010000101100000010000100000001000000000011100000000001000000000000100001000000000100000010000000000010000000000000000000000000000000000000001010000000000000000000000001000000010000000000000000000000000000000000000000000000000000000000000000000000000000000000000000000000000000000000000000000000000000000000000000000000000000000000000000000000000000000000000000000000000000000000010011100010111110111010101110010110101001101010110101101101011011001101100010001111101011011101010101000110011000010000001000000100000100100100100101000000010010100010100100100001111010000000000000000001001100010101101000000100000000000000010000000000000100000000000000000000100000000100001000100000100001000001000000000000000010000000100000000000000000000010000000100100000000000000000000001000010000000001000000000000000000000000000000000000000000000000000000000000000000110111011111011011000101111011010101011100100001101010001001010111101100111101001100011010100010010000100101000011010001010000110100000100110000000101000100100100010010101001000101011000001010100100001000110000000000110000010000000000100000000000000000110000010010011010000000000000001001000000000011000001000000000000000000000000001000000011100000000001101000001100000001010000000000000000000000000010100000000000000000000000???????????????????????????????????????????????????????????????????????????????????????????????????????????????????????????????????????????????????????????????????????????????????????????????????????????????????????????????????????????????????????????????????????????????????????????????????????????????????????????????????????????????????????????????????????????????????????????????????????????????????????????????????????????????1011111001100100001110011011000101001111001101010011011101110110000011001101001100101000000000100010001100100001000001010000101000000010000010010100010001000000101001110100000001100000000010100100000100000000000100000000000000000000000000000010000000000010000000000001100000001000000010000000000000000000000000000000000000000000000010000000000000000000000000000000010000000100000000000000000001100010100100010000000100100100011011000101011001000100001000000001000000010010000000010001000010000000000000000000000000000000000000000000000000000000000000000000000000000000000000000000000000000000000000000000000000000000000000000000000000000000000000000000000000000000000000100000000000000000000000000000000000000000000000000000000000000000000000000000000000000000000000000000000000000000000000000000000000000000000000000000000000000000000000000000000000000000000000000000000000010001011011101010110111010100111101101101101101011101011000011100100100000110111011011010001000000100011011100111001011010000110010110010010101000110011000001100001000101001100010010010000000000001010010000010001001000001000010100000000011100100000000000000000001100000000000011000000000000000000000000000000000000010001000000000000000100000001000000000000000000010000000100000010000001000000000000010000000000000100000000001100001010111010001100100001101001011001111011000011011100010101110001010000001111100101100100101011100100010000111010010010010000110101101000110010001001110000000000000100001001000001101011100000100011000011000111101000000000001000001001000010100000000000000000000000000000001000000100000001001001011000000000000010000000100010001001010100000110000010000000000001000000000010000100000001000000000011000001000100000000000000000000000000000010000000000000000000000100000000001000100100000000???????????????????????????????????????????????????????????????????????????????????????????????????????????????????????????????????????????????????????????????????????????????????????????????????????????????????????????????????????????????????????????????????????????????????????????????????????????????????????????????????????????????????????????????????????????????????????????????????????????????????????????????????????????????????????????????????110101100110010101011100111010111000011110110110110100100000110000110010110010001100100000000010101000000100010000100001110010000000001001100000001001001000011000100000001001110110100011000101000011101110000100001000010100000000000010000000100010100001000000001011010000000000010000000000000100001100010000001000000100000000000000001010000000100000000000000000000100000000000000101000010000100000000000010000000000000001000100000000000101000000111000000000011000100000???????????????????????????????????????????????????????????????????????????????????????????????????????????????????????????????????????????????????????????????????????????????????????????????????????????????????????????????????????????????????????????????????????????????????????????????????????????????????????????????????????????????????????????????????????????????????????????????????????????????????????????????????????????????????????????????????????????????????????????????????????????????????????????????????????????????????????????????????????????????????????????????????????????????????????????????????????????????????????????????????????????????????????????????????????????????????????????????????????????????????????????????????????????????????????????????????????????????????????????????????????????????????????

aRhspNA011857 11011101010101101011011001010101101011101111010110110101110100101101101010101011001011000101101010001001001000101001101100100011001111001001100001010111000110000001010101111000100110000100000010000010000000000000001100100000000000000000000000110000100000100000000001000000010000000000010000000100001001000010000011010000000000001001000000000001001001000100000000000010000001000000000000000000100010000000010000000000000000000000000010000000000000000000000001000000000010010110010011100110001101111100001111111100100010111001001001000000100011010101110110110101100110111000010001010001010011010100111010000110100000001001011010100010010001010000000001001000000000111100000101000100011000010111000100000001011010000000000001010001000000000001100010000000000000100001000000000000000000000000100100000000000100000000000001000110000000000000000000000000000000000000010001001100000001000001000100000000100000001000010000000000000000000000000000000000000000000001000000000000000000000000000000000000000000000000000000000000000100000000000000000000000000000000000000000000000000000000000000000000000000000000000000000000000000000000000000000000000000000000000000000000000000000000000000000000000000000000000000000000000000000000000000000000000000000000000000000000000000000000000000000000010110100010001101101110111010100101010101101101000100110101100011010100001011001010100010000100000001010110001001000000000001001001010011010001010110000110101001000000100100000010010001000010000011100000100000000101100000101000100000000000000000000010010010001000000010010000110010010000000000000000000001000010000001000000100100000000000000000010000000000000000000000000000000000000001000000000001000000000000000000000000000000000000000000000000000000000000000000000000000000000000010011011000000101000010110011110011010101010011001000100100001000100100000001101111000100000001010010000000000000000010010000010011000001000001000000100000000000001100000000100000110000000010000000000000000010010000010100000000000000010000001000001000000000000000000000000100000000000000000000001000000000000000000000000000000000000100000000000001000000000000000000000000000000010000000000000000000000000000000000000000001??????????????????????????????????????????????????????????????????????????????????????????????????????????????????????????????????????????????????????????????????????????????????????????????????????????????????????????????????????????????????????????????????????????????????????????????????????????????????????????????????????????????????????????????????????????????????????????????????????????????????????????????????????????????????????????????????????????101110010101100011101010100100101011010110001001010000010010100010001101010101100000010000100000011000101010011110110000001000101000001110101001001001100000011001000001010000001000011000001000000101000000100001010000000000000000100101001000000010100001000000000000010001010000100001000000000000000001011000001000000000000000000000000000000000000000010000000000001000000000000000000000000000000000010000000010000000000000000010000000010100000000000011011101010110110111010101110111101101001101010110101111101011011101101100010001011101001111101010101000110011000011000001000000100000100100110100101000000010010100010100100100001101010000000000000000001001110010001101000000100000000000000010000000000000100000000000000000000100000000100001000100000100001000001000000000000000010000000100000000000000000000010000000100000000000000000000000001000010000000001000000000000000000000000000000000000000000000000000000000000000000010110101111011011100101111011010001000100100001001010001001010111001100101100001101011011110010010000110111000011010001010001010100100100110000000101000100101010010010101001000110011000001010011100001000101000000000100000010000000100010000000000000000110000010010011010000000000000000101000100000011010000000000000000100000000000011000000011100000000000101000000110000001010000000000000000000000000010100000000000000000000000???????????????????????????????????????????????????????????????????????????????????????????????????????????????????????????????????????????????????????????????????????????????????????????????????????????????????????????????????????????????????????????????????????????????????????????????????????????????????????????????????????????????????????????????????????????????????????????????????????????????????????????????????????????????1010111001100100001110011011000110100111000101011001011101110110000011001101001000101000000000100001001100110001000011000000101000000010000011011101010001000000101001110100000001100000000010100100000100000000000110000000000000100000000000000010000000000010000000000001100000001000000010000000000000000000000000000000000000000000000010000000000000010000000000000000100000000100000000000000000100000000000000000000000000000000000000000000000000000000000000000000000000000000000000000000000000000000000000000000000000000000000000000000000000000000000000000000000000000000000000000000000000000000000000000000000000000000000000000000000000000000000000000000000000000000000000000000000000000000000000000000000000000000000000000000000000000000000000000000000000000000000000000000000000000000000000000000000000000000000000000000000000000000000000000000000000000000000000000000000000000001011011101010110111010100111101100100101001011111011100010100010100000110111011011000001000000101011011100110001011001000110010111010110101110111011000001100001000111000101000010010000000000001010010000010000001000001001010110000000001000100000000000000000001000000000000011000010000000000000000000000000000000010001000000000001000100000001100000000000001000001000000000000010000000000000000000000000000000010100000000000101001010010011001100100101111101011110101001000011011100010101000001010011001101001000100100101111110100010000010110100000000000110110001000110010000010100000100000000100000001000110101000000001000001010011000100101100000010011000001000010000100000000000000001000000000000011000000100000011000001001000000000000010100000010000000001000000000000000000000000000000100000000000000000000000000110000001000000010000000000000000000100001000000000000000000000000100000100000000000000000000000000???????????????????????????????????????????????????????????????????????????????????????????????????????????????????????????????????????????????????????????????????????????????????????????????????????????????????????????????????????????????????????????????????????????????????????????????????????????????????????????????????????????????????????????????????????????????????????????????????????????????????????????????????????????????????????????????????110001110101110100001010111011011010111010110110110100100000100100110011111000000000010100000010101000010100101110110001000010000000101001001010000000000100000000100000010000100000100101010000100001000100010000001000010000000000101010001000001000110001001010000000000000100001000000000000000000000000000000000001010000000000000000000010000000001000000000000000000100000000001001000000000000000000001000000000000000000100100001000000100000000000000000000000000000000001???????????????????????????????????????????????????????????????????????????????????????????????????????????????????????????????????????????????????????????????????????????????????????????????????????????????????????????????????????????????????????????????????????????????????????????????????????????????????????????????????????????????????????????????????????????????????????????????????????????????????????????????????????????????????????????????????????????????????????????????????????????????????????????????????????????????????????????????????????????????????????????????????????????????????????????????????????????????????????????????????????????????????????????????????????????????????????????????????????????????????????????????????????????????????????????????????????????????????????????????????????????????????????

aRhspNA011858 11010001010101010011010001001011101011101010010110110101100100111101101110001100001011000101001010001001001010111001111100100001101101001011110000010011100110000001000101101000110110000110000010000010000000000000000100100000000000011010000000110000101000100000001001000000010000000000010000000100001000100010000001001000000100001001000000000001001010000100110001000110000001000000000000000000010001000000100000000000000000000000000001000000100000000000000001000000000010010110000011000110001101111000000011010101110000110001001101000001100011010100110110110101100110101000010001010101011011010100101010000110100000000011000010000010010000010000000001000000100000011100000101000100011000000101000100000001011000000010000001010000000000000001000010000000000000100001000000000000000000000000100100000000000100000000000001000000000000000000000000000000000000000000010001001000000000000101000100000000001000000000010000000000000000000000000000000000000000000001000000000000001000000000000000000000000000000000000000000000000000000000000000000000000000010000000000000000000000000000000000000000000000000000000000000000000000000000000000000000000000000000000000000000000000000000000000000000000000000000000000000000000000000000000000000000000000000000000000000000000000000000000000000000010101000110010110101101110010000100010101101010001111110101100010110101001001001010100010000110000101010100001001000000010101000100010010010001001110000100110001000000000110000010000001000010000000100000100010000101100000101000100000000000000000000010010010001000000010010000100000010000000000000000000000000010000001000000100010000000000000000010000000000000000000000000000000000000010000000000001000000000000000000000000000000000000000000000000000000100000000010000000000000000000010011100100011011010010010011000001000101010010001101000100000100100010100100100100001000100001110011010000001000000000001000010001000000100001000000010000000000011000000000000001010000000000000000010000000000110000000110000000000000000000010000001000000000000000000000101000000000000000000000000000000000000000000000000000000000000000000000100000000010000000100010000000000000000000000000000000000100000000001000000000000??????????????????????????????????????????????????????????????????????????????????????????????????????????????????????????????????????????????????????????????????????????????????????????????????????????????????????????????????????????????????????????????????????????????????????????????????????????????????????????????????????????????????????????????????????????????????????????????????????????????????????????????????????????????????????????????????????????????????????????????????????????????????????????????????????????????????????????????????????????????????????????????????????????????????????????????????????????????????????????????????????????????????????????????????????????????????????????????????????????????????????????????????????????????????????????????????????????????????????????????????????????????????????????????????????????????????????????????????????????????????????????????????????????11011010010111110111010010111010101101010101010101111101101011010101101100010001111111011011101010101000110011000101000001000000100000100100100100101000000010010100010100100100001111010000000000000000000111100010001101000000100000000000000010000000000000100000000000000000000100000000100001000100000100001000001000000000000000010000001000000000000000000000010000000100100000000000000000000001000010000000001000000000000000000000000000000000000000000000000000000000000000000000110101111011010100101111011010101101010100001101010001001010111101100101101001100011010010010010000110111000011010001010000110100100100110000000111000100000110010010101001000111011000010010011100001000101000000000110000010000000100100000000000000000110000010010011000000000000000000101000100000011000001000000000000000000000000001000000011100000000001001000000100000001010000000000000000000000000010100000000000000000000000???????????????????????????????????????????????????????????????????????????????????????????????????????????????????????????????????????????????????????????????????????????????????????????????????????????????????????????????????????????????????????????????????????????????????????????????????????????????????????????????????????????????????????????????????????????????????????????????????????????????????????????????????????????????1010111001100100001110011011000111101101000100111011011111110110000011001101001100101000000000100010001100100001000001000000101000000010000011011100010001000000101001110100000001100000000010100010000100000000001110000000000000100000000000000010000000000010000000000001100000001000000010000000000000000000000000000000000000000000000010000000000000010000000000000000010000000100000000000000000111010011001011010000010100110101001001000011001001101101101010110100010000010001001000000000000000001000000100000000000000000000000000000000000001000000000000000000000000000000000000000000000000000000000000000000000000000000000000000000000000000000000000000000000000000000000000000000000000000000000000000000000000000000000000000000000000000000000000000000000000000000000000000000000000000000000000000000000000000000000000000000000000000000000000000000000000000000000000000000000000010010010111101010110011010100111001110001001001011101111100010100000100010110111011011011101000100100111011100111001111010001110010101010010101110111111000001100011000101000110000010010000000000011010010000010010001000001000010100100000011000100000000000000101101000000000000011100010000000000000000000000000010000010001001000000000000100000001100000000000001000001000000100000010000000100000000000000000000000001000000000000100001010010010001101100101011101011001111011001111011000001101100010010011001101000101110100101111110110010000010110010010000011010111001000010011001000100000000001000100010001100000111000000010000001000011000100101000000010011000001000001000100000000001000000101000000000000000000100000011001000001000000000000010100000000000001000000000000010000000000100000011000000010000000100000000000010000011000000000100000000000000000100000000010000000100000000000100000010000000000000000000000000???????????????????????????????????????????????????????????????????????????????????????????????????????????????????????????????????????????????????????????????????????????????????????????????????????????????????????????????????????????????????????????????????????????????????????????????????????????????????????????????????????????????????????????????????????????????????????????????????????????????????????????????????????????????????????????????????110001110001010101011010111001010000011001110110110100000000110001110000100001000010010100000010101000100010000110000011111000110001001001000000000001001000000100100001110000100000101001101100000001000100000100101000000100010000000011110001001000100001000000100001000000000000000000001100000100001000000000000000000000000000000000000000000000000000000000000000100000100101000000000000010000000000000000010000000000000000000000000000010000000000000100000000000000000001???????????????????????????????????????????????????????????????????????????????????????????????????????????????????????????????????????????????????????????????????????????????????????????????????????????????????????????????????????????????????????????????????????????????????????????????????????????????????????????????????????????????????????????????????????????????????????????????????????????????????????????????????????????????????????????????????????????????????????????????????????????????????????????????????????????????????????????????????????????????????????????????????????????????????????????????????????????????????????????????????????????????????????????????????????????????????????????????????????????????????????????????????????????????????????????????????????????????????????????????????????????????????????

aRhspRP011854 110110010101011101100100000010111000110010100101000001011001000001010010100100000010100011010010000010010010001000001011010000010010010010010000000000100000100000010000010100000000000000000000100000100000000000000001000000000000000000000000000000000000000000000000000000000000000000000000000000000010000000000000000000000000000000000000000000100000000000000000000000000000000000000000000000000000000000000000000000000000000000000000000000000000000000000000000000000000000101111000110101100011011011001010110111011100011100010011010000011000110101001101101100011001001010000100000100010100100100001011110001101000000000110000100000100100000100000000010000000000000111000001100001000110000011000001000000000110000000000000010100000000000000011000100000000000001000010000000000000000000000001001000000000010000000000000010000010000000000000000000000000000000001001111010011110101010011011111101011111111001110110100010100101011101001100001110000000101010111011101001101011111001000000100011101110000100001000000000000000111010000100000001000000100000110000000000000100001000000001100000000000000000000010000100010010000010010010000010000000000000000000000000100001000000110000000010000000000001010000000000000000000000000000000000000000000001000000000000000000000000000000010000100000101010000100101111011011110100011000101011011000011111101011000110101110010110010101100100011010001010111000010010000010001010001010100110100010101101001101110010001001101001010100100100000100000111000001001100011010011001110001000000000000000000000100100100010101000100101001000000110000010000000000000000000000000010000001000100000000000000000100000000000001000100000000000000000001100000000000100000000000000010000000000000000000000000000000000000000000000001000000000000000000000100111000000010000100100100110000010000000100100011010001000000001000000000001001000010000000000000110000000000000000000000000000000000000000000000001100000000000000000000000000000000000000000000000100000000001000000000100000000000000000000100000000000000000000000000001000000000000000000000000000000000000000000010000000000000000000000000000000000000000000000000000000000000000000000000000000000000000000000000000000000001111110011101100011011110110111010001100100101101000101001101011101111010101011101000101010110111100000010011001110001111010100001000101110100100010000110100000000001110000011001010100000100001000101011100010100011010010110100010000010000010110000000000000000100001000000001000000001001000100001000010000000000100010000000000001000000100000000000000001000000100000000000000000000000100001110000000000000000000000000000000000001000000000000000100000000000000000000000000000000000000000000000000000000000000000000000000000000000000000000000000000000000000000000000000000000000000000000000000000000000000000000000000000000000000000000000000000000000000000000000000000000000000000000000000000000000000000000000000000000000000000000000000000000000000000000000000000000000000000000000000000000000000000000000000000000000000000000000000000000000000000000000000000000000000000000000000000000000000000000000000000001001111001101111011101010111011011111000110101010010110110101101100100100101000111110001101110101010101011101100010000100100100110001100010010010010100100001101010001010011010000101110000000010100000000100110001010110100000010100000110000001000010000000010000001000000000000010000001010100100010000101010100000100000000100000011000010100000100010100000000001000000010100000000000000000000000100000000000000100000000001000100000000000000000000000000100000000000000000000000001101100111101101110010111101101010111010110100110101000110101011110000011010000110001110110001001000011010100001101100101000011010000010011100100101100011100001010000010100000001100100000000010000000100010001000000010000001000100000010000000000000000011000001001001100000000000001000000100010000010100000100010000000000000000000000100000000100000000000000100000000000000110000000000000000000000000001010000000000000000000000000111101010111110101101111001110111111111101010110110101001010001100101100010011001011000100101000100001000110111011100110000101100011011010000000001011001100101001000100000010100101000000000010000111010001010000000000010101011101010100000000000000000000000000100000010001000000001001000000001000100000000000000000000000000001000000001000010000000000110000000000000000000000000000000100000000000000000000000000000000000001000000000101110011000011110000000100000000000000000000000000000000000000000000000000000000000000000000000000000000000000000000000000000000000000000000000000000000000000000000000000000000000000000000000000000000000000000000000000000000000000000000000000000000000000000000000000000000000000000000000000000000000000000000000000000000000000000000000000000000000000000000000000000000000000000000000000000000110101010010001000001101010011010101100010101100100010101100001010100000000001000010001100100001100000100000101000000000000000000000010010000000100000000010110000000000000000000010000000000000001001001000001000000010000001000000000000011000000000000000000000000000000000000000010000000000000000000000000000000000000000000000000000000000000000000000000000000000000000000000000000000000000000000000000000000000000000000000000000000000000000000000000000000000000000000000000000000000000000000011110001010000001011101100110000100110001010101100001000000010000000011000101000000000000010001000110010000001000000111000000000000000100001000000000100000100010000000000000000000000000000000001000000100000100000100000000000000000100000000000000000000000100000000000000000000000000000000000000000000000000000000000000000000000100000000100000000000000000000010000000000001000000000000000000000000000000000000000000000010000100001101100110010011111111101110110100100111101111001011110100100010100110111010111010010111111011011010001011001111010100011100000100001001100101110001000000000010010010100100110100000000001000100001100010010001000000001100010100001111010000000000011001010000000000000100000010111101100110010100000000000010010000000000000000100000000000001000000000000000010000001000000000000000010000000100100000100000000010000000000000010000000000000000000000000001000001000000000100000010000000010011101011010000010000001111000010000000000001101000000000001001000000000010110010001000000001100001100110010100010010000100000000000001001100000001000000000000100000000000001000010100000000000000001010000000000000000000000000000000000000000001000000000000000000000000000000000000000000000000000000000000000000000000000000000000000000000000000000000000000000000000000000000000000000000000000000000000000000000000000000000000000000000000000000000000001001011101001111010111001110100100001111111101011001101100001110101111011110100000010110010100101011001011001001010101001000010101000010110000101111010000110001001000101100101100101110010000010011010011010000001010000101000110000000101011000010011001010000000000011000000001100000010000000001000000000000000000000000000001010100000000100000000000000010000000000001000000000000000000000100001000000010001000000000000000100000000000001000000010000000000000000100000000110000000000000000000000000000000000000000000000000000000000000000000000000000000000000000000000000000000000000000000000000000000000000000000000000000000000000000000000000000000000000000000000000000000000000000000000000000000000000000000000000000000000000000000000000000000000000000000000000000000000000000000000000000000000000000000000000000000110111001101010110011001010011000110100100011001100001000101001001100010001110000010110110100001000010010010101010000001000000000010000010001110000010001010000000000000001000000000011000000000000001000001000000100000000100000000010000000000000000000000000000000010000100000000000000000000000000000000000000000110000000000000000000000000000000000000000010000000000001000000000000000001000000000000000000100000000000000000000000000000000000000000000000000000000000000000000000000010

aRhspRP011857 000000000000000000000000000000000000000000000000000000000000000000000000000000000000000000000000000000000000000000000000000000000000000000000000000000000000000000000000000000000000000000000000000000000000000000000000000000000000000000000000000000000000000000000000000000000000000000000000000000000000000000000000000000000000000000000000000000000000000000000000000000000000000000000000000000000000000000000000000000000000000000000000000000000000000000000000000000000000000101110000111001100011011111000101110101011100111110010111010000011000110101001101101100011001101010000100110100010100110101001010110001111000000000010000100000100100100100000000010000001000001110000001010001000110000001000001000000010110000000000000010100000000000000011000100000000000001000010000000000000000000000001001100000000001000000000000010000100000000000000000000000000000000001001100111011100111010001011111100011111011000010110110010110001011001001100000100001011001000100010000001101000110000000000010000100111010100001000001000100000011100100000000001000000100010110000000100000000001000001000100000010001000000000010000000000010010010010000000010000000000000101000100000000001000000110000001000100000000001010001000000000000001000000000000000000000000000000000000000100000000000000000010000100001101010000000011111011101110101001100101011111010001001101010000100001000010110000101000100000000000010110000000000000010000010000000000100000000001100001000110010000000000000000000000000000000000001000001000000001011000001000000000000000000000000000100000100010000000100000001000000000000000000000000000000000000000010000001000000000000000000000000000000000000000000000000000000000000000000000000000000000000000000000000000000000000000000000000000000000000000000000000000000000000000100111000000010100100101100111000010000000100100010010001000000001000000000001001000010001000001000110000000000000000000000000000000000000000000000001100000000000010000000000000000000000000000000000100000000001000000001100000000000000000000100000000000000000000000000001000000000000000000000000000000000000000000000000000000000000000000000001000000001000000000000100000000000000000000000000000000000000000000000000000000001011100011101100011011110010111010000101101111101100101001001011001111010001111011000100110101110110000110111001110001101000100000000001010100000011000000101000000001010101000101011100110100001001101001000010000010010010010101010010010000010000000100000000000010101000000000000000101010010000000000010000000000100010000000000000000000000000000000000001000000000001001000000000000000000000000000000000000000000000000000000000000000000000000000000000000000000010111101010110101110101010011000101111011101010101001011011010111010111101010111100101001010001001101001001101110101100000100010100001011000101000110110010001101010100101000001100001100010100010110100001010010101000100001000000010010100110101001010100110100100000001010011011010001100000000000000100001100000100000000000000000000000000100010100101000000100000000100001000000100000000000000000000001000000010000100000010000000000000010100001000100001101111001011011011001000110001110110000110101010110111110101101010100110001100111110001111110101010100011001101010000010100100110101010010010010110100010001001010001010010110000101110000000011000001100100110001000110100110110000001000000001000000000100010000000000010000000010000000000000100010000100000100000100000000000000011000100100010000000001000000001000000010000000000000000000000000100000000000000100000000000000000000000000000000000000000000000000000000000000000101011010111101101010010111101101010101100010100100101000101101101100000010010000110001101000000000000001001100001101000101000101010000010011100110100100010010001010010110100000001010100001000010100000100010101000000100000001000100010010000000000000100011000001001101110000000000001000100100010000001110000000010000000010000000000000000000010100000000000100100000000000000101000000000000000000000000001010000000000000000000000010101101010011010101101111001110111101111101010110110101001010001100001100010010001011000100101000100100000110111011000110100101100011000010000000001011001000101001000000000010100001000000000010000111010101010000000001010001001111010100000000000000000000000000100000010001000000001001000000001000100000000000000000000000000000000000001000000000000000000000000000000000000000000000000000000000000000000000000000000100000001000000000101011100110111000111001101100010000011100110011100101100101000000000000100100100011000000000100000000100010000000001100000011000000001000001001000001000100000000000000000000000000000000000000000000000000000000000000000000000000000000000000000000000000000000000100000000000000000000000000000000000000000000000000000000000000000000000000000000000000000000000000000000000000000000000000000000000110101000011001000001101010011100101100010101100100010101100001100100000000001000010001000100001100000100000101000000000000000000000010010000010100000100010110000000000100000000011000000000000001001001000001000000010000001000000000000011000000000000000000000000000000000000000010000000000000000000000000000000000000000000000000000000000000000000000000000000000000000010000000000000000000000000000000000000000000000000000000000000000000000000000000000000000000000000000000000000000001001001101110101011101101011011100100100110100101010111110001110101010000011011000101010010111100011001001110010000111101110011000010001011010100011101100000110000100010000011101000001101000000000001101000101101000100000100001010000000001100000000000000000000000100000100000000100001001000000000000010000000000000000010100000000001001010000000100000000000000100101010000000000010000000100000000000000000000000001000000000000000000000000000000000000000000000000000000000000000000000000000000000000000000000000000000000000000000000000000000000000000000000000000000000000000000000000000000000000000000000000000000000000000000000000000000000000000000000000000000000000000000000000000000000000000000000000000000000000000000000000000000000000000000000000000000000000000000000000000000000000000000000000000000000000000000000000000000000000000000000000000000000000000000000000000000000000000000000000000000000000000000000010010101011000000010000001100100010000000000001110000000000001001000000000010110010011000000101100001100110010100010010000000000000000001001100000010000000000000100000000000001000010100000000000000001010000000000010000000000000000000000000000000000000000000000000000000000000000000000000000000000000000000000000000000000000000000000000000000000000000000000000000000000000000000000000000000000000000000000000000000000000000000000000000000000000000000001101011101001101011011111110000110001111011101101001010100001110001110111110101010010010110000101010001001110100001001001000010101011011010011101101000011101100101100000100101100001001011101010000011011101111010010000101000000100000100000000000001000010110001000110001000000000000000000010101000000000000001000000000000000000000000001000000000000010000000000000000010000010000000000000100000000000000001000000000000000000000000000000100000000000000000000000001000000000001000011000001011100010000000000110000000010000001001000000011000000000000000000000001000000000000000000000000000000000000000010000000000000000000000000000000000000000000000000000000000000000000000000000000000000000000000000000000000000000000000000000000000000000000000000000000000000000000000000000000000000000000000000000000000000000000000111111001001010101011000000011000111111111011010100001110111101001101111000110000000110110110001110111010010110010001001101010001000010011001000100110101001000000000001000000000001101110010000100001000000101000100010000100000000010100000001001100001000010000000000110101010010000000001000010000110110000000010010000010000000000000001000001000000000010000000000100001000000000000000000000000100000000000000101000000000000000000000000000000001000000000000000000000100000000000000000

aRhspRP011858 110110010101010101110100010010111100100010100101000011011001010111010010100000000010100001010010000010010010001000001010011000010010010010010000000100100000100000010000011000000001000001100000000000100000000000000001000000000000000000000000000000000000000000000010010000000100000000000000000000000000000000000000000000000000000000000000000000100000000000000000000000000000000000000000000000000000000000001000000000000000000000000000100000000000000000000000000000000000000101100000110001110111011111000111110111011100111110110011010000010000110101001101101100001001101110000110010100010110110100001011110001111000000000110000100000101100000100000000010000001000000111000001011001000110000001000001000000010110000000100000010100000000000000011000100000000000000100010000000010000000000000001001000000000001000000000000010000000000000000000000000000000000000001101101110111001110010001011101100010101011000010000100011100101001001001000000100000000101000101010110111100111111001000011100000101110000000001000001000100100111000000100000001000000100000100001001000000100001000000000000000010000000000000010010010000010000010000010100010000000000000100000000000010000000000110000000000001000000000010001000000000000001000000000000000001000000000000000000000000000000000000000000001110101101110001001011011011111110100011110101101110100001011101011110110101110010110010101101100011000000010111000100011000010001010010110100100100010011110001001100011001001010010000110100001000000010011000001001100011011010001000000010000000000000000000110110100010101000100101001000000100000000100000000000000000000000010000001010100100000000000000100000000000001000100100000000000000000100000000000100000000000000010000000000000000000000000000000010000000000000000000000000000000000000010010001100010001100100100110000010000010100110010010001000010001000000000001001000010000000001000100000000000000000000010000000000000000000000000001100000000000000000000000000000000000000000000000100000000001000000001100000000100000000000100000000000000000000000000001010000000000000000000000000000000000000000000000000000000000000000000001000000000100000001000100000000000000000000000000000000000000000000010000000000001111110110101100111010010010111011000101101111111001101001101011001111011101011011000100000100110101001110101001111101001010100010100000010100010010000000100000000001110000000001011000000100001001001011000010000011010010010000000000010000110000000000000000000010101000000001000000001000000100101000010000000000100010000000000000000000100000000000000001000000000000000000000000000000100000000000000000000000000000000000000000000100000000000001000000000000000110110101010110101010101010010000101101010001001101001001001010011010111101010110000001000010000001100000001001110101000000100010100000011000100100000110000001100100000001000000100001100000100000010100000000000101000000000000000000000000100000001010000000000000000000000000000010000100000000000000000000100000100000000000000000000000000000000000000000000000000000100000000000000000000000000000000000000000010000000000000000000000000000100000000000001001111011001111011101010110011011110100010101110110110111101101010110110101001111110101101110101010101011101100010100000100010110000010010011010110100010001001010001010010110100101110000000010100000000101110001000110000000010100000000000001000000000100010000000000010000000010000000000000100010000010000100000100000000000000011000001000000000000001000000001000000000010000000000000000000000100000000000000100000000000000000000000000000000000000000000000000000000000000000001101010101101101010010111101101010101010010100100101000110101101110000010110000101001101010001001000011011100001101100101000011010000010011010110101100010000101010001110100000001101100000011010110000100010001000000101000001000100010010000000000000100011000001001001110000000000001000100100010000001100000100010000000000110000000000100000010100000000000100100000000000000101000000000000000000000000001010000000000000000000000000101100010011110100100001000100000101001001000100000101000000001000001100000000001011000100001000100000000110111001000000000101000000000010000000001000000000000000000000000000100000000000000000000000000000000000000000000000000000000000000000000000000000000000000000000000000000000000000000000000000000000000000000000000000000000000000000000000000000000000000000000000000000000000000000000000000000000000000000000000000000000000000101011100110111000111101101100010110010100110101000101110101010000001000100100100010100000000010001000100010100100001010000010100000001000001001010001000100000010100111010000000100000000001010000000010000000000010000000000000000000000000000001000000000001000000000000110000000100000001000000000000000000000000000000000000000000000010000000000000000000000000000000000000000000000000000000000000110101010011001000001101010011010101100010101100100100101100001010100000000001000010001000100001100000100000101000000000000000000000000010000010100000100010110000000000100000000011000000000000001001001000001000000010000001000000000000010000000000000000000000000000000000000000010000000000000000000000000000000000000000000000000000000000000000000000000000000000000000010000000000000000000000000000000000000000000000000000000000000000000000000000000000000000000000000000000000000000000001001101111001111101001010011100100110100110101010101010001010101010000011011100101101010100000010001000110011000011101000111010011001011000100011111110000100001100010100010001000001100000010001101101100101001000100000100001011110000001100000000000000000010100100000100000001101001000000000000000000000000000000001000100000000010000100000000100001010000000000001000000000000010000000100000000000000000000000000100000000000010010111011010001111100101010011001100000001001111000101110111111011100000111001010101101000010011100000010010001000110001000011001101010011001000000000000011001000010010011101011000000000000000000000000000000000000000000000000000000000000000000000000000000000000000000000000000000000000000000000000000000000000000000000000000000000000000000000000000000000000000000000000000000000000000000000000000000000000000000000000000000000000000000000000000000000000000000000000000000000000000000000000000001000010000010000001100000000000000000000000000000000000000000000000000100000000000000001100000000100010000000010000000000000000001000000000000000000000000000000000000000000000000000000000000000000000000000000000000000000000000000000000000000000000000000000000000000000000000000000000000000000000000000000000000000000000000000000000000000000000000000000000000000000000000000000000000000000000000000000000000000000000000000000000000000000000000001100100111011011111111011100011000100001011010111110110110000110001110001100000000000000000001011000000000000000000000000000000000010001000000000000000000000000000000000000110000100001100010000000100100000010000100000010000000000000000000000000001000010000000000010000000000000000000000000000100000000000000000000000000000000000000000000000000000000000000000000000000000000000000000000100000000000000000000000000000000000000000000000000000000000000000000000000000000001000001001000000001000001010000100010000000100000000000000000100000000000000000000000000000000001000000000000000000000000000000000000000000000000000000000000000000000000000000000000000000000000000000000000000000000000000000000000000000000000000000000000000000000000000000000000000000000000000000000000000000000000000000000000000000000000000000110111011000001111011000110011000110010111011001100001000111101101100101000010000000110100010001000110010010100010001011011010001011010010001000000011001000001000001000100001000000000010010010010101000100000000100010010100100100011000000000001100100000110000000010001000000100000000000000111010111000000000000010100000000001000000000000000000000000000100000000000001000000000000000000000000010000000000000010000000001001000000000000000000000000000000000000000000000000000000000000

aCoboOP011819 11011101110101010011011001010101101011101111010110110001110101101101101010010010001011001101101010101101011010101011101000101011001101001011100100010011100110101001000101111010010100100010100010001010001011010000000101100001000001011010010011011000101100100000100101000000010010001010010010001100001000100010000001000000000010101111010100010101001010101010000100000010000000000100000000000000010001010010100100100000000100000000100010000000100001000010000001000010000100000111100010100111011101111100111111110100110101010011011101000100101101010100110110110000100110111010111011010001010001010000101111000110100000010010000010010000010000010000000001001000100000011100000101001100011000000100100100000001011000000000000101010010000000000001100010000000000100010001000000000000000000000000101000000000000100000000000001000000000000000000100000000000000000000????????????????????????????????????????????????????????????????????????????????????????????????????????????????????????????????????????????????????????????????????????????????????????????????????????????????????????????????????????????????????????????????????????????????????????????????????????????????????????????????????????????????????????????????????????????????????????????????????????????????????????????????????????10110101100001111101110111010000101010100101001000101011101101010100111001011001010110010101100100101111010010001100001000111111111000011010101001110101110101001100000010110100011010100100010001011101000100101101101001000011000100000000001111010000010000010001010101010010100100101100000010010000101000100000000011001000000100001010001000000000010000000010000100010010011000000010000001000000000001001000000100000100000000000000000000000000100000100000000000000000000000000100100000010011100110001011010110110011100011010011001011001111010101001100110010000100100110110100000001110011011010001000001000001110010010010100101000000000010000000100011010110100010101010000100001100100010100101010100101011010001000010000001010010100101000000000000000100010101000000000000000001000100000000000000001000001000100010000000000000100010000100000010000000100000000000000000000000000010000000000000101000100000010000??????????????????????????????????????????????????????????????????????????????????????????????????????????????????????????????????????????????????????????????????????????????????????????????????????????????????????????????????????????????????????????????????????????????????????????????????????????????????????????????????????????????????????????????????????????????????????????????????????????????????????????????????????????????????????????????????????????101101010101101010101010101100101011010111001001010010010010100110101101010101101000010010101000011001011010011110100000001010101001000111000001100001100100010000100000110000011000011000001010001101000010100001110000000010000000010100001000000010101000000000000000000000000010100001000000000100000000011000001000000001000100000000000000100000000000000000000000001000000000000101000000000000000000000000100010000000000000000000000000001000000000000011011100110101110111010101110111111101001101010100101101101101011111101101010001101011001111101011100000111011010101010101000101101010101110100101101000100110010100010100111110101011000001010101000000001000101010001101001000110001010000000010000000001110010001000100100000000100000000101001000100000100000000001000000000000000100100010000100000001001000000000000000000000100100000000000000101000000000000000100000000000000000000001000000000000000000000000000000000000000000011010101011011010100101010001010101101000101001001010001011010010101000101101001010011101000000010100010111000011001000100011010100000100110101101001000101011000100100001001010110101101001011100100001000101001000011010010010000100010010000000010000000110000010010101010000000000010001001000100010001100000001000001000101100000000000000100101000100100000001000010000101001000000000000000010000000000000100000000000000000000000???????????????????????????????????????????????????????????????????????????????????????????????????????????????????????????????????????????????????????????????????????????????????????????????????????????????????????????????????????????????????????????????????????????????????????????????????????????????????????????????????????????????????????????????????????????????????????????????????????????????????????????????????????????????1010111001000110101111011011010110101111001111011001011101010110001011001101001001101101010000110000001110101001000001000000101000000000000010011010010001110001101001110100000101100100000110110010000100000100000110000000000001000001000001010010000000000010000000000101100000011000000010011000000000000000000000000000000100000000000010000000010000000000010000000000010000000100000000000000000101100010100100010100011010110110101010000101011001000101011000010101100101011111000110111001110011011101000101010000010011010101000100100110010101100110100101111000100011000000000110000010000000111010010001010000000101000010000010010010110010100001011010000100000001010010000000100000010010100010000000000000001000010000000100000100000000000000000000010010110000100000101000000000000000000000000000000000000000010000000000010110000010000000000000000000011000000000100000000000000000010011010111101010111011010100111101010100101001010111011000010101010110101110111011001000101100100110010001110110000110010010100000111010100101101010101000100100010010101000101100000011000001010011000010000010001001000001000010100100000101000100001100000000000001000001000000010100010000010000000000001000000000001000001000000000000001000000011100000000000000000010000000000000010000000000100000000000000000000001000000000000100001010011010101110100111011101011101111011011111011110010111110011000001001111000101100100101111100100110100010110010010100000111101011100010010010001011010000000000110100101000000001000000001100101000011000110100100000100101000100010000000100000000000000000100010000000000000000100000011000000101000000000000000100000000000000001000000000000000000000000000000100000000010000000000000100000001001000000000000001000000000000000001000000000000000000000000010000000000000000000000000000000??????????????????????????????????????????????????????????????????????????????????????????????????????????????????????????????????????????????????????????????????????????????????????????????????????????????????????????????????????????????????????????????????????????????????????????????????????????????????????????????????????????????????????????????????????????????????????????????????????????????????????????????????????????????????????????????????????????????????????????????????????????????????????????????????????????????????????????????????????????????????????????????????????????????????????????????????????????????????????????????????????????????????????????????????????????????????????????????????????????????????????????????????????????????????????????????????????????????????????????????????????????????????????????????????????????????????????????????????????????????????????????????????????????????????????????????????????????????????????????????????????????????????????????????????????????????????????????????????????????????????????????????????????????????????????????????????????????????????????????????????????????????????????????????????????????????????????????????????????????????????????????????????????????????????????????????????????????????????????????????????????????????????????????????????????????????????????????????????????????????????????????????????????????????????????????????????????????????????????????????????????????????????????????????????????????????????????????????????????????????????????????????????????????????????????????????????????????????????????????????????????????????????????????????????????????????????????????????????????????????????????????????????????????????????????????????????????????????

aCoboOP011820 11011001110101101011011001010101111011101111010110111001110101101101111010110011011011001111101110001101111010101001011101001011001101001011100001010011100110001001000101101011101110100010110111001011101010011000101101100001101010111011100000000100101100101000000101000000110000000010010100000100011000110010010001000100000000100001011000011001001010001000000010000010000000001010001001010100010001000000100000100001000010000001100001000000000000000110011001000000000100000111100011110111011101111100111111110101110111010011011101000000101101010101110110110001100110111010110011010101010011111000101111000110100000010010011010010000010000010010101101001000100000011100000111100100011000001100100100100101011010000100000101010010100100000001101010000000000100010001010000001000000000000000101100000000000100000000000001000000000001000000100000000000000000000????????????????????????????????????????????????????????????????????????????????????????????????????????????????????????????????????????????????????????????????????????????????????????????????????????????????????????????????????????????????????????????????????????????????????????????????????????????????????????????????????????????????????????????????????????????????????????????????????????????????????????????????????????10110111101101111101110111010100101010100101001000111111101101010100111001011001010100010001100100101111010001001100001010101101111000011010101011110101100100001100000001110101011010100100010001011100000110101101101001100011000100000000001001010000010000011001000101000010100100101100000000010000101000110100000000110100000100000011001000000000010000000010000000010001011000000010000001000000000001001000000010000001000000000000000000000000010000010000000000000000000000000100100000010011000110001011011010111011100011010011001011001111000101001100110010000101100100110100000001111011011100001000101000001110010010010100111000000000010000000100011010110100010101011010100001100100010001101010100101000110001000010000001010010100000000001000000000100010101000000000001000001010100000000000000011010001000100010000000000000100010000100000000000000100000000000000000100000000000000000000000101000101000000000??????????????????????????????????????????????????????????????????????????????????????????????????????????????????????????????????????????????????????????????????????????????????????????????????????????????????????????????????????????????????????????????????????????????????????????????????????????????????????????????????????????????????????????????????????????????????????????????????????????????????????????????????????????????????????????????????????????101111010101101010101010101100001011011100001001010000010010100011101101010101101000010010100000011001010110011110110010001000101001000111001001000001100000010000101001010000011000011000001000001101000010110001010000100010000000100100001000000010100100000000010000000000000000100001000000000000100000011000001000000001000000000000000000000000000000000000000000001000000000000000000000000000000000010000100010000000000000000000000000001000000000000011011100011011110111010010110110110101100101010100101100110001010101011100010001010100011111101000100000101001000010000000000000100000100110100101100000010010010100010100011100001101000001000000000000000001100010001101000000100000000000000000000000001000000000000100100000000100000000100001000100000110000000001000000000000000001100001000000010000001000000000000000000000000000000000000000101000000000000000100000000000000000000000000000000000000000000000000000000000000000011010101111011001000101110011010101011000101001101001101011011010101000101101001010011101100010010100110111010011101001010111000100001100110101101011000001011001100101001001000110101001001011100100001000110001010011010000010000000010010000000010000000111000010011001010000000000010001001100100100001000000010000001000101100000000000000100101000100100000001000000000000010100000000000000000000000000010100000000000000000000000???????????????????????????????????????????????????????????????????????????????????????????????????????????????????????????????????????????????????????????????????????????????????????????????????????????????????????????????????????????????????????????????????????????????????????????????????????????????????????????????????????????????????????????????????????????????????????????????????????????????????????????????????????????????1010111001100110101010011101010110110101101111011011010101010110001111001101001001101101010000110000001100100001000001000000101000001000000010011000010001000001101001101100000001100100000010100010000100000100000100000000000001000001000001010010000000000010000000000101100000011000000010001000000000000000100000000000000001000000000010000000000000010000010001000000010000000100000000000000000101010010100100011001010100100110101010010101011001000111011100010101100100011011000110110000110011011101101001011100101011000101000100100100010101100110100101111000000011000000000111000010000000111010010000010001000110000010000010010010110010100001101011000100000001010010000000100000010010100100001000000000001000011000000100000100000000000000010000010100010000100000101000000000000000000000000000000000000000010000000000010100000010000000001000000010011000000000100000000000000000010010011011100010110110010100111001101100101001010101011010010101010100100110111011010000101100010110010001010100000010010100100000110010100101100110111000010100011011101101101010000010000010000011000010100010001010100001010010100100100001101100010100100000000001000000100000011000010000110000000000000000000000000000001000000000000000100000000101000100000000000001000000000000010000000000100000000000000000000001000000000000101001010010010101100101011011111011101101011010111011110011111101011000001001111010101110101101111110110111000010111010010100000110011001100010010000000010010000001000110100011000000001000000001100001000011000100100100000100101000100000000000100000000000000000100000000000000000010100000011001000101000000000000001100000000000000001000000000000000000000000000000100000000000000000000000001000000001000000000000001000000000000000001000000000000000000000000000000000000000000000000000000000??????????????????????????????????????????????????????????????????????????????????????????????????????????????????????????????????????????????????????????????????????????????????????????????????????????????????????????????????????????????????????????????????????????????????????????????????????????????????????????????????????????????????????????????????????????????????????????????????????????????????????????????????????????????????????????????????????????????????????????????????????????????????????????????????????????????????????????????????????????????????????????????????????????????????????????????????????????????????????????????????????????????????????????????????????????????????????????????????????????????????????????????????????????????????????????????????????????????????????????????????????????????????????????????????????????????????????????????????????????????????????????????????????????????????????????????????????????????????????????????????????????????????????????????????????????????????????????????????????????????????????????????????????????????????????????????????????????????????????????????????????????????????????????????????????????????????????????????????????????????????????????????????????????????????????????????????????????????????????????????????????????????????????????????????????????????????????????????????????????????????????????????????????????????????????????????????????????????????????????????????????????????????????????????????????????????????????????????????????????????????????????????????????????????????????????????????????????????????????????????????????????????????????????????????????????????????????????????????????????????????????????????????????????????????????????????????????????????

aCoboOP011834 11011001110101010011011001010011101011101110010110001001110101101101101010011010011011001111101100001001111010101001101101001011101101001001000100010011100111000001000101111010000100100010100010100010101011101001100101100001000000111011110000001011101000100000100101000001111000000010011010001100001100101010001001010000100000000001011010000010001010001001001000000010000010001000001000100000101001000000100000100000010010000001000010100000000001001000000001000011000100010111100011110111111101111100111111110100110111011001011101010100100011010100110110110001100110111011110011010101011011011000101111000110100000010010011010010000010000010010101101001000110000011100000101101100011010001100110100100101011010000100000101010000100100000001101010000000000100010001010000000000000000000000100100000000000010000000000011000000000001000000000000000000000000000????????????????????????????????????????????????????????????????????????????????????????????????????????????????????????????????????????????????????????????????????????????????????????????????????????????????????????????????????????????????????????????????????????????????????????????????????????????????????????????????????????????????????????????????????????????????????????????????????????????????????????????????????????10110100010101111101110111010101111010111111010000110110101101010100111001011001010100010101100100101111010001001100011110111101111011011010101010110101100110001110100010100101011010100100010001011101000110101101101101100011001100000000101001010000010000010001010101010010101100101000000010010000101000110100000010001000000101001010001000000000010000000001000100010010011000000010000001010000000001001000000010000110000000000000000000000000100000000000000000000000000000000100100100010011100110001011010010111011000011010011011011001101000101001100110001000100100110001110000001111010011000001000000100001101010010011100111001000000011000000000011010110100010100111010100001010101010001111010100001010110001000010000001010010100000000000000100001000010101000000000001100000010100000000000000011010001000100010000000000000100110000100000000000000100000000000000000000000000000000000000000101000100100010000??????????????????????????????????????????????????????????????????????????????????????????????????????????????????????????????????????????????????????????????????????????????????????????????????????????????????????????????????????????????????????????????????????????????????????????????????????????????????????????????????????????????????????????????????????????????????????????????????????????????????????????????????????????????????????????????????????????101111010111101010101010101100001011010111010011010010010110100011101101010101101001101010100000111001010011011110110010001000100000000111000001001001100100011000101000111000010100010000001001001101000010110001010000000010000000110100001000000010100100100000100000000010000010100001000010000100100000011000001000000000010100000000000000100001000000000000000000000000000000000101000000000010000000010001100010000000000000000000000000001000000000000011011100110101110111010100111010110100000101010100101101101011011001101010011001010110101111101000100000010101010011000001000101110010100100100100101000100010010100010100111100111011000001100000000000000001101010001100000100100000000000000010000000001000000000000000100000000100000000101001000110000100100000001000000000000000101000001000000010000001000000000000000000000000000000000000000101000000000000000100000000000000000100000000000000000000000000000000100000000000000010111001010011010100101010011010101101000101001001010001011010110100000101101001010011010100000010100110111000011101000110111000100001100110101101011000101101000010101001000110110101011001011011100001000100001010010110010010000000010100000000010000000111000110011001010000000000010001001100100010001100000001000000000101100000000000000100101000100100000001000010001001101010000000000000010000000000000100000000000000000000000???????????????????????????????????????????????????????????????????????????????????????????????????????????????????????????????????????????????????????????????????????????????????????????????????????????????????????????????????????????????????????????????????????????????????????????????????????????????????????????????????????????????????????????????????????????????????????????????????????????????????????????????????????????????1010111001100110101010011011000110110111101110111011010101010100001111001101101001100111010000110000001110110001000001000100101000000000000010011101011001110000101001110100000001110100000110100010000100000100000100000000000001000000010001010010000000000010000000000101100000011000000010001000000000000000000000000000000001000000000010000000010000000000010001000000010000000100000000000000000101010010100100010101010100110110101010000111111001000111011100010101000100011010000110011001100011001101101001011101011011011101000111100100010101010110101101111000100101100000000111000010000000111010010001010001000101000010000010010010111010010011101010100100000001000010000000100000001010100100000000000000001000010000000100000100000000000000010000010100010000100000101000000000000000000000000000000000000000010000000000010100000010000000000000000010011000000000100000000000000000010101010011010010110110010100111001101000101001010101101010010101000100100010111011001100101100100110010011100100001010010100110000110010100101100110111000100000001011101000100100000110000010010011000010000010001010100001000010100100000101000100000000000000001001000000100000001100010000010000000000000000000000000000001000000000000000100000001010000100000000000001000000000000011000000000100000000000000000000001000000000000110001010010010101110100111011101011101101011100111011110010111110010000001001101010101100100101111100100011000010111010010100000110101001100010010000000010010000000000100100101010000001010000001100101000011000100100100000100101000100010000000100000000000000000100010000000000000010100000011001000101000000000000000100000000000000001000000000000000000000000000000100000000010000000000000000000000001000000000000101000000000000000010000010000000000000000000000000000000000000000000000000000??????????????????????????????????????????????????????????????????????????????????????????????????????????????????????????????????????????????????????????????????????????????????????????????????????????????????????????????????????????????????????????????????????????????????????????????????????????????????????????????????????????????????????????????????????????????????????????????????????????????????????????????????????????????????????????????????????????????????????????????????????????????????????????????????????????????????????????????????????????????????????????????????????????????????????????????????????????????????????????????????????????????????????????????????????????????????????????????????????????????????????????????????????????????????????????????????????????????????????????????????????????????????????????????????????????????????????????????????????????????????????????????????????????????????????????????????????????????????????????????????????????????????????????????????????????????????????????????????????????????????????????????????????????????????????????????????????????????????????????????????????????????????????????????????????????????????????????????????????????????????????????????????????????????????????????????????????????????????????????????????????????????????????????????????????????????????????????????????????????????????????????????????????????????????????????????????????????????????????????????????????????????????????????????????????????????????????????????????????????????????????????????????????????????????????????????????????????????????????????????????????????????????????????????????????????????????????????????????????????????????????????????????????????????????????????????????????????????

aLedeCB011900 11011101010101010011011001010101101011101110010100111001110101010110101010110010101010001010101010001101001010100101111101010011010101001011011001101111000110010001001101101010110100010100110011000010000100011000000100100001100001011011100000111011100100101000000001001010010000000000010000000100001000101010000001010000000000101001010111010010001010011000110101110011000000000000000011000001100010000000100011000101000100000010000010000100000010000000000010000000011010010111101010000110101101101000011011010100110100011011001001100001000101010100110110010100100100101010010001010001010001010000101110100110100000010110001010100010110100110100001001001000100010011100000111101100111000001100110100100011011010000110101001010010000010000001101011000000100000100001100000100000010000000010100100100000000100100000000001000010000001000000000000100000000000000????????????????????????????????????????????????????????????????????????????????????????????????????????????????????????????????????????????????????????????????????????????????????????????????????????????????????????????????????????????????????????????????????????????????????????????????????????????????????????????????????????????????????????????????????????????????????????????????????????????????????????????????????????10101000100010100101100100010100100010101101100100110101001101010100111001010001010100100001100100011111010001001101001001101101111011010010111000110010100110101001000111010101010010100010010001011110000100101010101100100011101101000011001100000100010000010001010101010010010100101010000000010010110000110000000000011000100100000011010000000000010000001001000000010010001010000000000001000001000000000000000100000000000000010000000100000000010000000000000000000000000000001000000000000011100110001011010010110011000001100001011011001101010101000010110010001001100100101010000001110011010000101001000000001001010000010010111010000001101000100000011010100101010001010000100000000000010010101010100000010010001000000000001010010010101000000000000000000000101000000000001000001000000000000000000000000000000100010000000000000000100000100000000000000100000000000000000100000000000000000000000101000100000010000??????????????????????????????????????????????????????????????????????????????????????????????????????????????????????????????????????????????????????????????????????????????????????????????????????????????????????????????????????????????????????????????????????????????????????????????????????????????????????????????????????????????????????????????????????????????????????????????????????????????????????????????????????????????????????????????????????????101111010101101010101010100100101011010110001001010010011010110111101111010101101001010010101000011000010011011111010000001000101100000110101010001001100001010000101001010000001000011000101010101101000010100001010001000100000000100101001000000010100001000000010000000010100010100001000000000100000000001000001000000000000000000000000000100000000000000000000000101000000000000000000000000000000001000000100000000000000000000000000000001000001000000011011101010110110111010101111010110101010101010100101101101011010101001000011001010110011011001010101000111001010100000101000000101011000100100100101000100010010000010100111100101011000001000101000000001001110010001101000001100001010000000011001001001000100001000000100000000100000000101001000100000100100000001000100000000000110000000001000010000010000000000000000000110000000000000000001001000000000000001000000000000000000000000000000000000000000000000010000000000000000001010101011010011000101010011010101001000100001101010001011010010100000100100001000011101000000010100110011010010100010110000010100110100110100100101000100101000100100001000000110101100010010101100001000101000001011010010010000000010010000000010000010110000010011001100000100000010001001000100010101000000010000000000101000000000000000100001000100100000001000000000000010100100000000000000000000000000100000000000000000000000???????????????????????????????????????????????????????????????????????????????????????????????????????????????????????????????????????????????????????????????????????????????????????????????????????????????????????????????????????????????????????????????????????????????????????????????????????????????????????????????????????????????????????????????????????????????????????????????????????????????????????????????????????????????1010111001001110101111011011100110110101001101011001011101010100001011001101001001101101100000100001001100111001010011000100101001001111000010010101010001010010101001110100000111100110000010101010100100000110000100001000000101101011001111010010000000000010000000000101101000011000000010010000000000000000000000000000000100000000000010000000000000010000010001000000010000000100000000000000001011010010100100010010110010100110101010000111011001000101110100010101000101101010000110010001000011001101001001010000011011011110000101101010000101000111100101111001100010000000000110001010010000110010010010010001000101000010000010011010110010100001101000100100000000010000000000100100000010100000001001000010010000010001000100000100000000000000010000101010010011000000101000000000000000000000000000000000000000010000010000101000000100010000000000000010000000000001000000000000000000000001011011101001010111010100111001100000101001010101101000011000000010100100111001011010100101000100011001110100000111001001110000110001100101010110011010001000001001100101001000000001000000010001000010000010001000000001000010100000000101000100000000000000000001000001000000110000110000000000000000000000000000000000001000000000000000010000001000001100000000000001000000000000001000000000000000000000000000000000000000000000101001010011010101100100011011111011101101001000111011100011101110001001001001111010101110100101111100100010001010110100010100000110100001010010010000010100010000001000100101001000001101000001001000001000011000100100100000000001001100010000100100000000000000001100010000000000000000100000011000000101000000000000000000000000000000001000000000010000100000000000000100000000000000000000000000000000001000000000000001000000000000000000000100000000000000000000000000000000000000000000000000000??????????????????????????????????????????????????????????????????????????????????????????????????????????????????????????????????????????????????????????????????????????????????????????????????????????????????????????????????????????????????????????????????????????????????????????????????????????????????????????????????????????????????????????????????????????????????????????????????????????????????????????????????????????????????????????????????????????????????????????????????????????????????????????????????????????????????????????????????????????????????????????????????????????????????????????????????????????????????????????????????????????????????????????????????????????????????????????????????????????????????????????????????????????????????????????????????????????????????????????????????????????????????????????????????????????????????????????????????????????????????????????????????????????????????????????????????????????????????????????????????????????????????????????????????????????????????????????????????????????????????????????????????????????????????????????????????????????????????????????????????????????????????????????????????????????????????????????????????????????????????????????????????????????????????????????????????????????????????????????????????????????????????????????????????????????????????????????????????????????????????????????????????????????????????????????????????????????????????????????????????????????????????????????????????????????????????????????????????????????????????????????????????????????????????????????????????????????????????????????????????????????????????????????????????????????????????????????????????????????????????????????????????????????????????????????????????????????????

aLedeCB011901 11011001110101010011011010010101101011101110000100110001100101101110101010010110001010100101011001010001011010101011010101001011010101001011000100101111100110100101000110101010110010100100000010010010011010011001000101000001100000010010000000101000100100101000000101000001010000000000010100000100001000111010010001010000000000101001011101000010001010001000000100000110000000000000100001000000100110000000100001001101000100000001000010101000110010000010000010000010011000010111101010100110101101101100010111010100110110011001011001000001000101010100110110100100000100101011010001000001011001010000101010000110100100010110001010010010111000110000101001001000101000011100000101101100111100001100100100100001111010000010101001010010000100000001100011000000100000100001100000000000000000000001100100000000000010000100000101000000000001000001000000100000000000000????????????????????????????????????????????????????????????????????????????????????????????????????????????????????????????????????????????????????????????????????????????????????????????????????????????????????????????????????????????????????????????????????????????????????????????????????????????????????????????????????????????????????????????????????????????????????????????????????????????????????????????????????????01000000000001001000000010000000000010000000000000000000000000000000000000000100000000000000000000000000000000000000000000000000010000001000100000010001000100000000000000000000000000000100000000000000000000010000100000000000000000000000000000000000000000000000000000000000000000000000000000000000000000000000000000000000000000000000000000000000000000000000000100000000000000000000000000000000000000100000000000000000000000100000000000001001000000000000001000100000001100000000000000000011000110001011011010110011000011010101011011001101010101000100110010101010100100001010000001110011010000101001000000001010010010010010110010000001010000100000011010100101010101011000100000000010010100101010100001010100001000000000001010010100001000000000000000011000101000000000001100001000100000000000000000000001000100010000000000000000100000100000000000001000000000000000000100000000000000000000000101000100000010000??????????????????????????????????????????????????????????????????????????????????????????????????????????????????????????????????????????????????????????????????????????????????????????????????????????????????????????????????????????????????????????????????????????????????????????????????????????????????????????????????????????????????????????????????????????????????????????????????????????????????????????????????????????????????????????????????????????101111010101101010101010100100101011110110101001010010010110100110101101010100111101010010100000011010010111011110110000001000101000000110101001101001100001010000101001010100001000011000101001001101000010100101010000100000000000100101001100000010101001100000000000000010100010100001000000000100000000001000001000010001000000000000000000100000000000000000000000001000000000000001000000000000000001000000100000000000000000000000000000001000001000000010111100110110110111010101110110110110110101010100101101101001010101101000010001010110001011101000100000111001010100000100000001101010100100100100101000100010010100010101011100101111000001010101000000001001110010001101001000100001010000100010000000001010110001000000100000000100000000101001000100000100100010001011000000000000110000000000100010001010000000000000000001001000000000000000000101000000000000001000000000000000000000000000000000000000000000000000000000000000000001010101010011010000101010001010101010100100001101010001011010010100000101100001000011101000000010100100111010010011001010000010100110101010101101001000100101000100100001000000110101100001010101100001000101000001010010000010000000010010000000010000010110000010011001010000000000010001001000100000011000000010000000000100000000000001000000001011000100000001000000000000010100000000000000010000000000100100000000000000000000000???????????????????????????????????????????????????????????????????????????????????????????????????????????????????????????????????????????????????????????????????????????????????????????????????????????????????????????????????????????????????????????????????????????????????????????????????????????????????????????????????????????????????????????????????????????????????????????????????????????????????????????????????????????????1010111001001110001111011011010110000111000110111001011111010100001111001011000101101101000010100000011001100000000011000001101000000000000110110100000001000000101001100100000001000000000000100000000100000000000100001000000000000000000001000000000000000010000000000101100000001000000010001000000000000000000000000000000100000000000100000000000000010000000000000000010000000000000000000000001001010110000100010101011010100110101011000100011001000101111100010101000101101010100111011001101011101101101001010000010101001101000100101010010101000110100101111000100001000000000111001010010000111010010100010001000100100010000100011010110010100001101001000100100000010000000000100100010010100000001001000010010000011010000100000100000000000000010000110010010000100100101000000000000000000000000000000000000000010000100000101000000100000000000000000100100000000001000000000000000000000010010111000001010010010100111001100000101001010101101010010101000100100110111001010100101101000100010001100110000010010100110000110010100101100011001000011100001010100001001000000011000010000011000010000010001001000001000010100100000101010100000100100000000001000000100000101000010000100000000000000000000000000000001000000000000001000000001000000100000000000010000000000000100000000000100000000000000000000010000000000000101001010010010101100100101011001011101101011010111011110011101110010001001001111010101110100101111100100110101010110110010100000110101001100010010000000100010110001000100101001010000011000001001000001000011010100100100000101001001100010001000110000100000000001000010000000000000000100000011001000101000000000000000000000000000000001000000000000000000000100000000100000000010000000000000000000000001000000000000001000000000000000010000010000000000000000000000000000000000000000000000000000??????????????????????????????????????????????????????????????????????????????????????????????????????????????????????????????????????????????????????????????????????????????????????????????????????????????????????????????????????????????????????????????????????????????????????????????????????????????????????????????????????????????????????????????????????????????????????????????????????????????????????????????????????????????????????????????????????????????????????????????????????????????????????????????????????????????????????????????????????????????????????????????????????????????????????????????????????????????????????????????????????????????????????????????????????????????????????????????????????????????????????????????????????????????????????????????????????????????????????????????????????????????????????????????????????????????????????????????????????????????????????????????????????????????????????????????????????????????????????????????????????????????????????????????????????????????????????????????????????????????????????????????????????????????????????????????????????????????????????????????????????????????????????????????????????????????????????????????????????????????????????????????????????????????????????????????????????????????????????????????????????????????????????????????????????????????????????????????????????????????????????????????????????????????????????????????????????????????????????????????????????????????????????????????????????????????????????????????????????????????????????????????????????????????????????????????????????????????????????????????????????????????????????????????????????????????????????????????????????????????????????????????????????????????????????????????????????????????

aLedeCB011902 11010011010101010011010001010101101011101111010110111001110101101101111010001011011011100111101101010111111010100101101110011001011110101000100000010111100110101110111101101101101110100011100010101010011010000000000101100000000000111010000000001000100100110000000001000000011010000000001000000110001000111001000011000000000000000001011000000000100101000100000000001010000000000100000000010000001000100000010000110000000000000000000000000000000000000000000000000000000000010111100010100110001101111100011111010100110111011011011101010000100101010101110110110101100110101011010001010001011010110000101011100110100000011010011010100010110000110100101001001000010000011100000101100100111010011100110100100101011010000010101001010001000100000001101011001000010000100001010001010000010000000000100100000000000100000100000011000000000001000001100000101000010000000????????????????????????????????????????????????????????????????????????????????????????????????????????????????????????????????????????????????????????????????????????????????????????????????????????????????????????????????????????????????????????????????????????????????????????????????????????????????????????????????????????????????????????????????????????????????????????????????????????????????????????????????????????10111000000001110101110111010011111110110111101101111111101111010110111001011001010110110001110110111111011110101101101101111111111111011010101111111101110101111100100111010101010011100100010001111110010110101101101101100011101101000000001111111100011000010001010101010010010100111100000000010011100000110000000000001010000110000011001000000000010000000001000000010010001000000000000001000010000000000000000100000000000000010100000000000000100000100000000000000000000000010000000000010011100100001011010010110011000011010101011011001101010101000110110010101000100100100110000001110011001000001001000000001010010011010010110000000001010000100000011010100101010101011000100000000000010010101010100001010110001000000000001010010000001010000010100000000000101000010000010000001000100000000000000010000001000100010000000000000000100000100000000000000100000000000000000100000000000000000000000101000100000010000??????????????????????????????????????????????????????????????????????????????????????????????????????????????????????????????????????????????????????????????????????????????????????????????????????????????????????????????????????????????????????????????????????????????????????????????????????????????????????????????????????????????????????????????????????????????????????????????????????????????????????????????????????????????????????????????????????????101100010100101011101010100100001011010111110001010010010110100110101111010101101001010010100000011001010011011110110010001000101101000111001010101001100000010000101010010000001000011001001010101101000010100101010000100010000000100101001100000010100101100000100000000010100010100001000000000100000000001000001000000001000000000000000000100000000000000000000000001000000000000001000000000000000001010001110000000000000000000000000000001000001000000011011011010101110111010101110110110101010101010100101101101011010101101000010001101011001011001010100000111001010010000101000001101011000100100100101000100010010100010101001100101111000001010101000000001001110010001101001100100101010000100011000000001010100001000000100000000100000000101001000100000100100001001010000000000000010000000000100000001010000000000000000000001000000000000000000001000000000000001000000000000000000000000000000000000000000000000000000000000000000010111001010011010000101010011010101100100101001101010001011010010100000101100001000011110010000010100110111000010101101010011010100110100111001101001000100101010100100001000000110101100010010101100001000101000001001010010010000000010100000000010000010110000010011001100000000000010001001000100010011000000001000000000101000000000001001000001011000000000001000000000000001100000000000000000000000000000100000000000000000000000???????????????????????????????????????????????????????????????????????????????????????????????????????????????????????????????????????????????????????????????????????????????????????????????????????????????????????????????????????????????????????????????????????????????????????????????????????????????????????????????????????????????????????????????????????????????????????????????????????????????????????????????????????????????1010111001101110101111011011010110100111001111011001011101010100001111101101001001101001000100100010001000110001010011010000101001000110000010011110010001010000101001110100000101100100000010100010100100000010000100001000000101101001001101010010000000000110000000000101101000011000000010001000000000000000000000000000000100000000000100000000010000000000010001000000010000000100000000000000000111010010100100010000010010100110101001100110111000100101001100010010100000101110010010010001000010000010000100100000000000000000000000000100001001000001000101000000000000000000000100000000000000000010000010010000000100000010000000000010100000000000000000010000000000000000000000000000000100000000000000000000000000010000000000000000000000000000000000010000000010000000101000000000000000000000010000000000000000000000000000101000000100000000010000000000000000000000000000000000000000000010010111110001011010010100111001100000101001010101011010010100000100100110111011001100101001000100011001100100001011010001110000110010100001110011001010001100011010101001010000000010000001010011000100000010001001000001000010100100000101000100001100100000001001000000100000001100110000100000000000000000000000000000001000000000000000100000001000000100000000000010000000000000100000000000100000100000000000000010000000000000101001010010010101100100111011101011101101011000111011110011101110011000001001111000101010100101111110100110101010110010100100000110101011110011010100010100010100001000110100101010000011000000001000001000011000100100000000011001001100010001000110000100010000000110010000000000000000100100011001000101000000000000010000000000000000001000000000000000000000100000000100000000010010000000010000000000001000000000000001000000000000000010000010000000000000000000000000000000000000000000000000000??????????????????????????????????????????????????????????????????????????????????????????????????????????????????????????????????????????????????????????????????????????????????????????????????????????????????????????????????????????????????????????????????????????????????????????????????????????????????????????????????????????????????????????????????????????????????????????????????????????????????????????????????????????????????????????????????????????????????????????????????????????????????????????????????????????????????????????????????????????????????????????????????????????????????????????????????????????????????????????????????????????????????????????????????????????????????????????????????????????????????????????????????????????????????????????????????????????????????????????????????????????????????????????????????????????????????????????????????????????????????????????????????????????????????????????????????????????????????????????????????????????????????????????????????????????????????????????????????????????????????????????????????????????????????????????????????????????????????????????????????????????????????????????????????????????????????????????????????????????????????????????????????????????????????????????????????????????????????????????????????????????????????????????????????????????????????????????????????????????????????????????????????????????????????????????????????????????????????????????????????????????????????????????????????????????????????????????????????????????????????????????????????????????????????????????????????????????????????????????????????????????????????????????????????????????????????????????????????????????????????????????????????????????????????????????????????????????????

aLeleCB010205 11011001010101010010011001010101101011101110010100110001100101101101101010010010001010000100001000000001001000100000001010100001001101001001000000010111000010010001001101001010000100000100010010000010000010001001100101100000000001010010101000001000101100101000000001000001010000010000110100000100011001101010001001000000001000100000010000000110001010001000100001100010000010001000010000100000100010000000100001000000000100000000000010000000010000000000000010000000001000010111100010000110001101001010011111010111110101011011011001100101000111010100110110110101100110111101010001010001010010110000101111110110100000011011000010100000000000110000000001000000100000011100000101000100111000001100010100000001011000000010000001010000000000000001100010000000000000100001100000000000000000000000100100000000000100001000000100000000000000000000000000000000000000000????????????????????????????????????????????????????????????????????????????????????????????????????????????????????????????????????????????????????????????????????????????????????????????????????????????????????????????????????????????????????????????????????????????????????????????????????????????????????????????????????????????????????????????????????????????????????????????????????????????????????????????????????????10111010010101111101110111010101111010110111101100110011101101010100111001011001100110110001100100001011010010101101001100101101111010011011011010110000110110001000000111000101010010000000010001011110000100001001101001100000001100010001000101000010010000010001010001010010000100101010000000000000100000010000000000011000000100000010000000000000010000000000000000010000000000000000000001010000000000010000000000000000000000000000000000000000000000000000000000000000000000000000000000000011000110001011010011110011101011010011011011101101010101010110111010001100110110001110000001110010010000001000000000010001010000111000100000000000100110000000011000100100000001011000000000000000010110101010100000011010000010000000001010010110100000000000000000001010101000000001101000001000000000000000000110000010000000000000000000000000100000100000000000000000000000000000000000000000000000000000000101000100000010000??????????????????????????????????????????????????????????????????????????????????????????????????????????????????????????????????????????????????????????????????????????????????????????????????????????????????????????????????????????????????????????????????????????????????????????????????????????????????????????????????????????????????????????????????????????????????????????????????????????????????????????????????????????????????????????????????????????101111010101101010101010100100101011010110001001010010010110100110101111010101110000010010100010011010010011011111010000001000101100001110101010001001100100011000101000010100011000011001001000001111000010100001010000000000000000000101001000000011101001000000000000011000000000100001000000000000000000001000001000000000000000000000000000000000000000000000000000001000000000000000000000000000000000000000100000000000000000000000000000001000001000000011011100011011110111010101110110110101010101010100101101111011011101101100010001101110001011001010101001111010010010010100000110100010101100100101101000100111010100010100111100101011000001010000000000001001101010001101001100100000000000000000000101000100110000000000100000000100000000101001000100000100000000001000100000000000010000001000000000000001000000000000000000000000000000000000000101000000000000001000000000000000000000000000000000000000000000000000000000000000000010111011111011001001101010001010101101000101001001010001011001010100000100100101000011010100000000000000111000010101001010000010101000101010000000101000100001000100100010000000111000000001000101100001000101000000011000010010000000010100000000010000000110000010011001100000000000000001001000000000001000100000000000000100000000001000000000001000000100000001000000000000010100000000000000000000000000000100000000000000000000000???????????????????????????????????????????????????????????????????????????????????????????????????????????????????????????????????????????????????????????????????????????????????????????????????????????????????????????????????????????????????????????????????????????????????????????????????????????????????????????????????????????????????????????????????????????????????????????????????????????????????????????????????????????????1010111101001100101010011011000110101110000101011000011101010100011011011101001001100100000000100000001100100101000001000000101000101000000010010010011001000000101001110100000101101000000010100001000100000010000100001000000001000000000001000010000000000010000000000101100000001010000010001000000000000000100000000000000001000000000100000000000000010000010010000000010000000000000000000000001001101010100100010000110010100110101010000110011001001001011000010101100101101010000111011001100011101101101001011000000011001101000101100100000101010111000101111001000001000000000111001000000000110010010000010000000100000010000100011010110010100000001010100100000000000000000000100000000000100000000000000000010001010000000100000100000000000000010000010011010000000000101000000000000000000000000000000000000000010000000000011000000100000000010000000000000000000000000000000000000000000011010011100010111010010100111001101000101001010101011000010100000000100110110011010100101100000100010010100100000010000100110000110010100101000011001010001000001000100000100010000010000001000001000100000010000001000001000000100000000101000000000100000010000001000000100000001000000000000000000000000000000000000000001000000000000001000000001000000000000000000010000000000000100000000000000000000000000000000010000000000000101001000010010101100100111011101011101101001000011011110011101110101000001001111010101110100101111101100010001010110000010000000110101001000010011000000100000100000100100001001000000001000000000100001000011000100100000000000001000100010000000100000000000000000100000000000000000000100000011000000101000000000000000000000000000000001000000000000000000000000000000100000000000000000000000000000000001000000000000001000000000000000000000000000000000000000000000000000000000000000000000000000??????????????????????????????????????????????????????????????????????????????????????????????????????????????????????????????????????????????????????????????????????????????????????????????????????????????????????????????????????????????????????????????????????????????????????????????????????????????????????????????????????????????????????????????????????????????????????????????????????????????????????????????????????????????????????????????????????????????????????????????????????????????????????????????????????????????????????????????????????????????????????????????????????????????????????????????????????????????????????????????????????????????????????????????????????????????????????????????????????????????????????????????????????????????????????????????????????????????????????????????????????????????????????????????????????????????????????????????????????????????????????????????????????????????????????????????????????????????????????????????????????????????????????????????????????????????????????????????????????????????????????????????????????????????????????????????????????????????????????????????????????????????????????????????????????????????????????????????????????????????????????????????????????????????????????????????????????????????????????????????????????????????????????????????????????????????????????????????????????????????????????????????????????????????????????????????????????????????????????????????????????????????????????????????????????????????????????????????????????????????????????????????????????????????????????????????????????????????????????????????????????????????????????????????????????????????????????????????????????????????????????????????????????????????????????????????????????????????

aLeleCB010206 11011001010101001010010101000101101011001010010100010001100101010101101010001010001010100101001000001001001000100001011100101011101101001001000000010101000010010001010101101000110100000100000010000010000110001001000100100000000010010010000010100010100000000000000001000000010000001000011000000100011010100010000001010000010011101011001000000010001010010100100000000010000001000000000000100000100010000000100001000011000000000000000010000000100000010010000010000000001000010000000011000111001101111100000010000100100000000000001001000001000000000000000000000000000000000000000000000000010000000000000000000010100000000000000000000000000000100000000000000000000000000000000000000000000000000000000000000000000000000000000000000000000000000000000000000000000000000000000000000000000000000000000000000000000000000000000000000000000000000000000000000000000000000????????????????????????????????????????????????????????????????????????????????????????????????????????????????????????????????????????????????????????????????????????????????????????????????????????????????????????????????????????????????????????????????????????????????????????????????????????????????????????????????????????????????????????????????????????????????????????????????????????????????????????????????????????10111010101001101101101111011000101010101111010000110111101100010100111001011001010110100000100100011011010001101100001010101101011101010010111000110000110100001000100110100111010010000000010000001101000100000010101001100000001000100000000011000010010000010001010001010010000100101000000000000000100000010000000000011000000100000000010000000000010000000000000000000000000000000000000001100000000000000000000000000000000000000000000000000000000000000000000000000000000000000000000000010111110010101011010111111011110111100101011001001010010101000110111010001100110110000010010001110011010000000000000000001000010000010001000000000000010000000000001000000000000000000000000000000000010010101010100000000110001000000000001000010000101010000000000000001000101000000001000000000000000100000000000010000000000000000000000000000000000000000000000000000000000000000000000000000000000000000000000101001000000010000??????????????????????????????????????????????????????????????????????????????????????????????????????????????????????????????????????????????????????????????????????????????????????????????????????????????????????????????????????????????????????????????????????????????????????????????????????????????????????????????????????????????????????????????????????????????????????????????????????????????????????????????????????????????????????????????????????????100101010101101011101010100101101011010110111001010010110010110010101101010100100000010010101011011010010011011110010010001000101100001110110011001001000000010010101000010100101000011010101001000101000000000001010000000000000001000001001000000010101001000000000000000000000000100001000000000000100000001000001000010000000000001000000000000000000000000100000000001000000000000000000000000000000000000000110000000000000000000000000000001000001000000011011100010111110111011101111010111101101001010100101101101011010101001100011001010101001001001010101100010001010100010001000110100010101100100011101000100110010100010101010011011011000001000000000000000101101010001101001101100000000000001000000101001000100000000000100000000100000000000001000100000000000000001000100000000001110000001000000010000010000000000000000000000000000000000000000101000000000000001000000000000000000000000000000000000000000000000000000000000000000010111001111010001000101010001100101001000101101011010101000010010100100101100101000011110100000000000000011000010100101010000010101000101010100000011000100001000100100110000001011000000100100100000001000100000000010100010010010000010000000000010000000110000010101001100000000000000010001000000000001000100000000000000100001000001000000000001000000100000001000000000000010100000000000000000000000000000100000000000000000000000???????????????????????????????????????????????????????????????????????????????????????????????????????????????????????????????????????????????????????????????????????????????????????????????????????????????????????????????????????????????????????????????????????????????????????????????????????????????????????????????????????????????????????????????????????????????????????????????????????????????????????????????????????????????1010011101001100001010011011000100110110000100110000011101010100011011001101001001100100000000100000001100100101001001000010101000001000000010010000010001000000101001110100000101101000000010100000000100000010000100001000000001000000000001000010000000000010000000000111100000001000000010001000000000000000010000000000000100000000000100000000000000010000010001000000010000000000000000000000010001010010000100010000010010100110101001000101111001000111011100010101000100000000100110110001100011000001000001010000000000000000000000000100000000000000000101101000000000000000000110000000000000010010010000010000000100000010000000001010110010000000000000000000000000000000000000100000000000000000000000000000000000010000000000000000000000000000000000000000000000000000000000000000000000000000000000000000000000000000000000000000000000000000000000000000000000000000000000000000000000000000010011001001010010010100011001000000101100010101001000010000100100001100110001001000000100000100010001100101001010000010110000000000000100000010000000001000001000100000100000000000000000000001000010000000000000000001000000000000000101000000000000000000000001000000000000000000000000000000000000000000000000000000000000000000000000000000000000000000000000000000000000000000000000000000000000000000000000000000000000000000101001000011010101100100011011110110001101000100011011000011101010000000011001111010101110100101111010101010001010010001000000000110010001100010011000001100000000001000100000101000001001000000000000001000011000100101000000000001000100010000000100000000000000000100000000000000000000100000011000000100000000000000000000000000000000001010000000000000000000000000000100000000000000000000000000000000001000000000000010000000000000000000000000000000000000000000000000000000000000000000000000000??????????????????????????????????????????????????????????????????????????????????????????????????????????????????????????????????????????????????????????????????????????????????????????????????????????????????????????????????????????????????????????????????????????????????????????????????????????????????????????????????????????????????????????????????????????????????????????????????????????????????????????????????????????????????????????????????????????????????????????????????????????????????????????????????????????????????????????????????????????????????????????????????????????????????????????????????????????????????????????????????????????????????????????????????????????????????????????????????????????????????????????????????????????????????????????????????????????????????????????????????????????????????????????????????????????????????????????????????????????????????????????????????????????????????????????????????????????????????????????????????????????????????????????????????????????????????????????????????????????????????????????????????????????????????????????????????????????????????????????????????????????????????????????????????????????????????????????????????????????????????????????????????????????????????????????????????????????????????????????????????????????????????????????????????????????????????????????????????????????????????????????????????????????????????????????????????????????????????????????????????????????????????????????????????????????????????????????????????????????????????????????????????????????????????????????????????????????????????????????????????????????????????????????????????????????????????????????????????????????????????????????????????????????????????????????????????????????????

aLeleCB011917 11011101010101010010010010100101101011001110010110110001100101000101101010001010001010100101011000001101001000101101001100100011001101001001000000010101000110100001000100101000010100000100010010000010000010011001000100000000100000010010000000000000101100100000001001000000010000000000010000000100001001100010000001000000000000100000010000010001001010001000000000000010000001000000000000100000010001000000100001000001000010000000000001000000000000000000000001000000001000010110110111000111001101101101011111010111110101011011001101100101000101010100110110110101100110101010010000010001110010110000101110010110100100000110000010100000010000110000000001000000100000111100000101101100111000001100010100000001011000000011000001010000000000000001100110000000000000100001100000000000000000000000100100000000000100010000000100000000000000000000000000000000000000000????????????????????????????????????????????????????????????????????????????????????????????????????????????????????????????????????????????????????????????????????????????????????????????????????????????????????????????????????????????????????????????????????????????????????????????????????????????????????????????????????????????????????????????????????????????????????????????????????????????????????????????????????????10110100010010110101110101010000100010100101100000110111101101010100111001011001010110010000110010011111010000101101001000101101111010010011001001110000110110001000100111000111010010000000010001011101000110001001101010000000000000000001000111000000010000011001010001010010000100100010000000000000100000010000000000011000000100000010010000000000010000000000000000000000000000000000000001110000000000010000000000000000000000000000000000000000000000000000000000000000000000000000000000001011001010001011010010010001010011000001011011001100000100000100111010001000110110101110000001110011001000000000100000010000010010011100000000000000010000000000001010000100000000010000000000000000010110101010100000010110000000000000010100010010100000000000000000001000101000000000001000001000000000000000000000010000000000000000000000000000100000100000000000000000000000000000000000010000000000000000000101000100000010000??????????????????????????????????????????????????????????????????????????????????????????????????????????????????????????????????????????????????????????????????????????????????????????????????????????????????????????????????????????????????????????????????????????????????????????????????????????????????????????????????????????????????????????????????????????????????????????????????????????????????????????????????????????????????????????????????????????100111010101101011101010100100101011010110011000110010010110110010101101010101100001001110100010011000000011011100110001001001101000001110001001001001100100011000101000010010011000011000101000001101000000100101010000000000000010000001001000000010101001000000000000110000000000100001001000000000000000001000001000001000000000010000000000000000000000000100000000001000000000000000000000000000000000000000010000000000000000000000100000000100000100000011011101010111110110010010101010111101011011110100100101101011010001001100010001010101001001011010101000010001000010001101000101100010100100100101101000010010010000010100101010011011000001000000000000000101101010001001000101100000000000001000000001001000100000000000100000000100000000000000000100000000000000000100010000000000010100001000000010000001000000000000000000000000000000000000000101000000000000000100000000000000000000000000000000000000000000000001000000000000000010111001111010001000101010001000101001000100001001010001010000010100000100000001000011011000000000000000111000010011101100000010101000111010100000101000100000100100100001000000110000000001000101000001000100000001010100100010001010010000000000010000000000000010111001000000000000000100001000000000001000000000000000000100000000000000000000001000000100000001000000000000001000000000000010000000000000000100000000000000000000000???????????????????????????????????????????????????????????????????????????????????????????????????????????????????????????????????????????????????????????????????????????????????????????????????????????????????????????????????????????????????????????????????????????????????????????????????????????????????????????????????????????????????????????????????????????????????????????????????????????????????????????????????????????????1010111001001100101011011011010110101101000101011000011101010100011011001101101001001000000000100000001100100101001001000001101000001000000001010000010001000000101001110100000011101000000010100001000100000010000100001000000001000000000001000010000000010010000000000101100000001000000010001000000000000000100000000000001000000000000010000000000000010000010001000000010000000000000000000000000101010010100100010010010100100110101001000111011001000101011000010101000001011011000110011001101011001101100001011000000001010101000100100100000101000110100101111100000001100000000110001000000000110010010000010000000100000010000010011010110010100000001000000100000000000000000000100000000000100000001000000000001000010000000010000100100000000000010000010000010000000000101000000000000000000000000000000000000000010000001000010100000100001000010000000010000000000000000000000000000000000001010111010101010110010110111001000000101001010101101000010001000100000100111011011000101100001100010001110100000010000100110000101010100101000010101000001000001000100001100010000000000001010001000010000010000000000001000000100000000101000000000100000000000001000000100000000000000000000000000000000000000000000000001000000000000001001000001000000000000000000010000000000000100000000000000000000000000000100010000000000000101001000011011001100101011011111011101101001000011011000011111010100010001001111010101100100101111100110010001010110000010000000110100001000010110100000100010100000000100000001000000001000001000000001000011000100100000000000001000100000001000100000000000000000100000000000000000000100000011000000101001000000000000000000000000000001010000000000000000000000000000100000000000000010000000000000000001000000000000001000000000000000000000000000000000000000000000000000000000000000000000000000??????????????????????????????????????????????????????????????????????????????????????????????????????????????????????????????????????????????????????????????????????????????????????????????????????????????????????????????????????????????????????????????????????????????????????????????????????????????????????????????????????????????????????????????????????????????????????????????????????????????????????????????????????????????????????????????????????????????????????????????????????????????????????????????????????????????????????????????????????????????????????????????????????????????????????????????????????????????????????????????????????????????????????????????????????????????????????????????????????????????????????????????????????????????????????????????????????????????????????????????????????????????????????????????????????????????????????????????????????????????????????????????????????????????????????????????????????????????????????????????????????????????????????????????????????????????????????????????????????????????????????????????????????????????????????????????????????????????????????????????????????????????????????????????????????????????????????????????????????????????????????????????????????????????????????????????????????????????????????????????????????????????????????????????????????????????????????????????????????????????????????????????????????????????????????????????????????????????????????????????????????????????????????????????????????????????????????????????????????????????????????????????????????????????????????????????????????????????????????????????????????????????????????????????????????????????????????????????????????????????????????????????????????????????????????????????????????????????

aMamoOC011836 11011001110101010111011011010101111011101110010100110011110101101101111010110111111011101111011001001001101010101101010100101011010101001011101011011011100111110101001000101010110100100101100011011010101010010100100101100000101011011010100000011010100100100000100101001010111000001011010000000100011000100010010001010000110110100001010110110110001010010100000101110110100000001010001011000000101010000000100001001000100100000000010010000000000110000010000110010010011000010111100011110110011101111101111111010100110111010011011101100101001101010101110110100101100100111101010101110101011011011000101111000110100010010011001011100010010000110101001101001000110000011100000101101000111010001110110100100101011010100100001001010000000100000001101011001000000100100001010000100000010000000000101000010001001000000000110011000000011001000001000010110100100000000????????????????????????????????????????????????????????????????????????????????????????????????????????????????????????????????????????????????????????????????????????????????????????????????????????????????????????????????????????????????????????????????????????????????????????????????????????????????????????????????????????????????????????????????????????????????????????????????????????????????????????????????????????10110101101010110101010111010100111010100101010100111011101101010110111001011001010100010000101000101011010010101101001010101111011001010011101110110110100101101000000110100101010010100100010001111101000110101010101001110111001111100100001110101000011010010001010101010010010100110010000000001000100000110100000000001000100100000010001000000000010000001010000000010011001000010000101010100000000110000000010110001000000000000000000000000000100000001010000000000000010000010001000000010011000110001011010010111011100111010011011001101101000101010110110010000100101110101100000001110011010000101001001000010101011010010100111010100001110000100000111110110101010101011010100001100000010100101010100001011010001000000010101010010001010110100010000001000010101000000011000000000000100000000000000011000010000100010000000000000000101000000000000000001000000001000000000000000000000000000000100101001000001011000??????????????????????????????????????????????????????????????????????????????????????????????????????????????????????????????????????????????????????????????????????????????????????????????????????????????????????????????????????????????????????????????????????????????????????????????????????????????????????????????????????????????????????????????????????????????????????????????????????????????????????????????????????????????????????????????????????????101101010101101010101010100110001011010100010001010010010110100110101111010101101000010010100000011001011011011101011000001100101100010110100010101001100001010000000001010100011000011000101000101101000010100001010000000000000000100101001000000010101000100000100000010000000010100001000010000100000000010000001000000000000000000000000000000000000000000000000000001000000001001010000000000000001000010000010000000000000000000000000000000010001000000011011101011011110111010101110110111101001101010100101101111001011110101100010001101111001111001011100010111101010100000100000110100011001100100101101000100110010100010100111100101011000001100101000000001001101011101101001100110001010000011011000101001010111011000000100000000100000000101001000100001010101010001011000000000100011000010000101010001010000000000000000000110000000000000000001001000100000100001000001000000000000000000000000000000000000000000000000000000000000010110101011011001000101110001010101100100101001111110011101011010110000101101001101011110100100010100010111000011101101100011010100110101010101101011000101101000100100110001000111001000010011101101001000110001000011110010010000000010100001001000000001110000010011010100000000000010001001000100010101000000110100000000101100000000001001000101001000000000001000000000000010100000000000000000000100000001000000000000000000000000???????????????????????????????????????????????????????????????????????????????????????????????????????????????????????????????????????????????????????????????????????????????????????????????????????????????????????????????????????????????????????????????????????????????????????????????????????????????????????????????????????????????????????????????????????????????????????????????????????????????????????????????????????????????1010111001001110001111011011010110101111001101011001011101010100001011001101101001001001000000100000001100100001000001000000101101000001000010011101010001100000111001110100000001111000000010100100000100000100000100001000000001000000100100100010000000010010000000000101100100001000000010001000000000000000000000000000000010000100000100000001000000000000010001000000010000001100000000000000001001101010100100010010010100100110101011001111011001001001011000010101000101101010000110011001101011001101101001110000011011010101011010101000001001000111100101111001000101000000100111000010100000111010010100010001000101010010000010011010110010100001001010100100000001010000000000100000010010100100010000100100110000010000000100000010000000000000000000010000010000100000101000000001000000000000000000100000000000010001000001001000001000000001010000000001000000000000000000000000000000010001010111101010111010010100111001101010101001010101011011010100010000100110111011101000101000010100010010100100000110001100110100111010101001011010101000101000011011101101001010000010000010000001000101000101001011000001001010100100000101000110000100100000001001000000100000011000010000100000000000000000000000000000001000000000000001000000001010000000000000000010000000000000100100000000100000100000000000000010000000000100101001010011110101100100111011101011101101010000111011110011101110001001001011111010101110100101111110110110001010110100110000000110101001101010010010000100010100001000110100101000001011000001001000110000011000000100000000010101000100010000000110000000000000000100010000000000000000100100011001000101000000000000000100000000000000001000000000000000000000000000000100000001010000000000010000000000001000000000000010100000000000000000000000000000000000000000100000000000000000000000000000000??????????????????????????????????????????????????????????????????????????????????????????????????????????????????????????????????????????????????????????????????????????????????????????????????????????????????????????????????????????????????????????????????????????????????????????????????????????????????????????????????????????????????????????????????????????????????????????????????????????????????????????????????????????????????????????????????????????????????????????????????????????????????????????????????????????????????????????????????????????????????????????????????????????????????????????????????????????????????????????????????????????????????????????????????????????????????????????????????????????????????????????????????????????????????????????????????????????????????????????????????????????????????????????????????????????????????????????????????????????????????????????????????????????????????????????????????????????????????????????????????????????????????????????????????????????????????????????????????????????????????????????????????????????????????????????????????????????????????????????????????????????????????????????????????????????????????????????????????????????????????????????????????????????????????????????????????????????????????????????????????????????????????????????????????????????????????????????????????????????????????????????????????????????????????????????????????????????????????????????????????????????????????????????????????????????????????????????????????????????????????????????????????????????????????????????????????????????????????????????????????????????????????????????????????????????????????????????????????????????????????????????????????????????????????????????????????????????????

aMamoOC011837 11011001110101010011010001010101111011110111011110001101100110101110111010110010001010010101011010010001101010101101011101000011101101001001000100100011000010000001000101100000000100000101010011000010100010101001000101110000100011011010100000001000101000100000000001010000010100001010011100100100001001011010010001010000000000100001010000011010001010010100000101100110100100001000001001000000100010000010100001101001000100000000000010000000100010000000000010000010001000010111100011001110011101111101111111010101110111010001011101010100101101010101110110100101100100111101010101110101010010011000101110000110100010010011011010100010010100110101001101001000100000011100000101101100111100001110110100100101011010100110001001010001000100000001101011001000000100100001110001100000010000000000101000010000101000010000110011000000000001000001000011010100000000000????????????????????????????????????????????????????????????????????????????????????????????????????????????????????????????????????????????????????????????????????????????????????????????????????????????????????????????????????????????????????????????????????????????????????????????????????????????????????????????????????????????????????????????????????????????????????????????????????????????????????????????????????????10101000010000110101010101010001101010110111101101110011001101011000111001011001010110110001101000101011010010101110001000101100111101010010101000110000100100101100000010010101011010000010010001101110010100101010101101110010101111000010001110100000111011010001010101010010010100100010000000001000100000110100000000010000100100000010011000000000010000000010000000010010001000010000101010100000000001010000100100000100110000000000000000000100100000000100000000000000010000010001000000010011100110001011010010110001100111100011011011101101000110010110110010000100101110101100000001110011010000101001001000010100010110010001001010100001101000110000111000110101010101011010100001101000010010101010100001001110001010000010101010010110000101010100010001000010101000000010001000000000100000000000000000000010000100010000000000000000011000100000000000001000000000000000001000000000000000000000100101001000001010000??????????????????????????????????????????????????????????????????????????????????????????????????????????????????????????????????????????????????????????????????????????????????????????????????????????????????????????????????????????????????????????????????????????????????????????????????????????????????????????????????????????????????????????????????????????????????????????????????????????????????????????????????????????????????????????????????????????101111010101101101101010100100001011010110001010010010011010100110101101010101101000010010100000011000010011011101011000001010101000010110101010101001100001010000100001010000011010011001001000101101000010000001010001000000000000001001001000000010101000100000100000000000000010100001000010000100000000010000001000000000000000000000000000000000000000000000000000001000000001000010000001000000000000000000110000000000000000000000000000001100000000000011011101010101110111010101101010110100000101010101101101110101011101101100010001111011001111101010101010111101010100010101000110101011010100100101101000101010010100010100111100011011010001100101000000101001101011001101001101110001010000101011001001011010110011000000100000000100100000101001000100001000100000001011000000000100001001010000101000001010000000000000000001010000000000000000001001000100000000001000001000000000000000000000000000000000000000000010000000000000000010110101111010001000101010011010101101000101001101010001011011010110000101101001100011110100100010100110111000010100001010011010101000100010101101011000101001000100100010001000111001000010011101100101000110001001011010010010000000010100010001010000001110000010011001100000000000010001001000000100101000001000100000000001100000001001001000011001000100000000000000000000010100000000000000000000100000000100000000000000000000000???????????????????????????????????????????????????????????????????????????????????????????????????????????????????????????????????????????????????????????????????????????????????????????????????????????????????????????????????????????????????????????????????????????????????????????????????????????????????????????????????????????????????????????????????????????????????????????????????????????????????????????????????????????????1010011001000110001011011011000110110111001101010001011101010100001011001111001001101001000000100000001100100001000001000001101101001001000010011100010001100000111001110100000011111000000010100100000100000110000100001000000001000000001001000010000000010010000000000101100010001000000010001000000000000000000000000000000010000100000100000001010000010000010001000000010000000100000000000000001001010010000100010000010010100110101010000111011001001001011000010101000101100010000110011001100011001101001001110000000011011000111111000100000101000111000101111001000010000001010110001100100000111010010000010001000101010010000010011010110010100001001010100100000001010000000000100000000010100100001000010000110000010000000100000100000000000000010000010000010000100000001000000000000000000000000000100000000000010001000001001000001000000001000000000000000000000000000000000000000000000000000111100000000000000011000000000000000000000000000000000000000000000000000000000000000000000000000000000000000000000000000000000000000000000000000000000000000000000000000000000000000000000000000000000000000000000000000000000000000000000000000000000000000000000000000000000000000000000000000000000000000000000000000000000000000000000000000000000000000000000000000000000000000000000000000000000000000000000000000000000000101001010010010101100110011011101011001101101000111011110001101101010000011011101010101110110101111100100110001010110010100000000110101001001010010000000100010000001000110000101000000111000001000000100000011000000100100000110101000100010000000100000000000000000100010000000000000000100000011001000101000000000000000100000000000000001000000000000000000000000000000100000001010000000000010000000000001000000000000010100000000000000001000000000000000000000000100000000010000000000000000000000??????????????????????????????????????????????????????????????????????????????????????????????????????????????????????????????????????????????????????????????????????????????????????????????????????????????????????????????????????????????????????????????????????????????????????????????????????????????????????????????????????????????????????????????????????????????????????????????????????????????????????????????????????????????????????????????????????????????????????????????????????????????????????????????????????????????????????????????????????????????????????????????????????????????????????????????????????????????????????????????????????????????????????????????????????????????????????????????????????????????????????????????????????????????????????????????????????????????????????????????????????????????????????????????????????????????????????????????????????????????????????????????????????????????????????????????????????????????????????????????????????????????????????????????????????????????????????????????????????????????????????????????????????????????????????????????????????????????????????????????????????????????????????????????????????????????????????????????????????????????????????????????????????????????????????????????????????????????????????????????????????????????????????????????????????????????????????????????????????????????????????????????????????????????????????????????????????????????????????????????????????????????????????????????????????????????????????????????????????????????????????????????????????????????????????????????????????????????????????????????????????????????????????????????????????????????????????????????????????????????????????????????????????????????????????????????????????????????

aMamoOC011948 11011101010101011011011011010101101011101111010100110101110101101101111010110011011011001101101010001001001010100001011001000011001101001011110100011011100111010001101000101010010100100100110011000010000010111101000111100001101011011010100000001001111100100000100001001000110000000010010111000100001001010010000001010000100000000001010000010010001010010000100001100110100000001010100011000100010010000010101001000001100100000000000010000000100010000000000010000010001000010111100010100111011101111001111111010100110111010001001101100000101101010101110110100101100100101011010101010001010011010000101110000110100010010011011010100010010100110100101001001000110000011100000101101000111100001101110100100101011010100110001001010000000010000001100011001000000100100001100000100000010000000000101000010000100100010000110001000000011001000000000000110100000000000????????????????????????????????????????????????????????????????????????????????????????????????????????????????????????????????????????????????????????????????????????????????????????????????????????????????????????????????????????????????????????????????????????????????????????????????????????????????????????????????????????????????????????????????????????????????????????????????????????????????????????????????????????10101000100000100101110101010000101010100101100000110111101101010100101001011101010110110010101000101011010010101101001000101101111001010010101010110110110110111000000110010101011010000100010001111110000100101010101001110010001111110000001110100000010001010001010101000010010100100010001000010000100000110100000000010000100100000010101000000000010000001010000000010010001000010000100010101000000001000000010110000100000000000000000000000010100000001010000000000000010000010001000000010011100110001011010010111001100111100011011011101101000110010110110010000100101110100100000001110011010000101001001000001101011010010101001010100001010000101000110110110101010101011010100001010000010010101010100001011110001001000010000100010100000001000101010001000010101000000011000000000000100000000000000000000010001100010000000000000000101000000000000000001000000001000000000000010000000000000000100101000100001011000??????????????????????????????????????????????????????????????????????????????????????????????????????????????????????????????????????????????????????????????????????????????????????????????????????????????????????????????????????????????????????????????????????????????????????????????????????????????????????????????????????????????????????????????????????????????????????????????????????????????????????????????????????????????????????????????????????????101111010101101010101010100101001011010110001010010010010010100010101101010101100000010010100000011000010010011111011000001010101000010111101010001001100000010000100001010100011010011001001000001101000001010001010001000000000000100101001000000010101000100000000000010000000010100001000010000100000000010000001000000000000000000000000000000000000000000000000000001000000001001010000000100000000000000000110001000000000000000000000000001010000000000011011101011011110111010101100111110110000101010101101100100001011101001000011001101110001111011010101010110101010100010100000110101011001100100101101000100110010100010101011100101011010001100101000000101001101010101101001001110001010000101011001101010010010011000000100000000100000000101001000100001010100010001011000000000110001000001000100000001010000000000000000000110000000000000000001001000000000000001000001000000000000000000000000000000000000000000010000000000000000010110101111011001000101010011010101101000101001001010001011011010110100100101001010011111000000010100110111010010010101010010010101001101010101101011000100101000100100010001000101001100010010101100101000100001001011010010010000000010010010001010000001110000010011001100000000000010001001000100100101000000110100000000101100000001000001000101001000000000001000000000000010100000000000000000000000000000100000000000000000000000???????????????????????????????????????????????????????????????????????????????????????????????????????????????????????????????????????????????????????????????????????????????????????????????????????????????????????????????????????????????????????????????????????????????????????????????????????????????????????????????????????????????????????????????????????????????????????????????????????????????????????????????????????????????1010111001001100101011011011010110110111001101011011011101010100001011001101101001001001000000100000001000100001000001000000101001001001000010011100010001100000111001110100000011111000000010100100000100000110000100001000000001000000001001000010000000010010000000000101100100001000000010001000000000000000000000000000000001000100010100000001000000000000010001000000010000001100000000000000001001101010000100010000010010100110101010000101011001001001011001010101000101101011000110011001101011001101101001110000001011011110101011000100000101000110100101111001000001000001000110001010000000111010010000010001000101000010000100010010110010100001001010000100000001000000000000100000000010100100001000100100110000010000000100000100000000000000010000010000010000100000101000000000000000000000000000100000000000010000000001001000001000000000100000000000100000000000000000000000000000000010010011100001010010010100111011101000101001010101011010010100000100101110111111011000101100010100011001100100000010010100110100101010100101011010001000101000001011101101001010000011000000000001000100000010001010100001001010100100000001000100000100100000001001000000100000001100010000100000000000000000000000000000001000000000000001000110001010000100000000000010000000000000100100001000100000100000000000000010000000000100101001000010010101100100101011001011101101011010111011110011111101010000011011111010101110101101111100100110001010110010100000000110101001101010010000000101010010001000110101001000000101000001000000101000001000000100100000110001001100010000000100000000000000000100010000000000000000100100011001000101000000000000000100000000000000001000000000000000000000000000000100000000010000000000010000000000001000000000000010100000000000000001000000000000000000000000000000000010000000000000000000000??????????????????????????????????????????????????????????????????????????????????????????????????????????????????????????????????????????????????????????????????????????????????????????????????????????????????????????????????????????????????????????????????????????????????????????????????????????????????????????????????????????????????????????????????????????????????????????????????????????????????????????????????????????????????????????????????????????????????????????????????????????????????????????????????????????????????????????????????????????????????????????????????????????????????????????????????????????????????????????????????????????????????????????????????????????????????????????????????????????????????????????????????????????????????????????????????????????????????????????????????????????????????????????????????????????????????????????????????????????????????????????????????????????????????????????????????????????????????????????????????????????????????????????????????????????????????????????????????????????????????????????????????????????????????????????????????????????????????????????????????????????????????????????????????????????????????????????????????????????????????????????????????????????????????????????????????????????????????????????????????????????????????????????????????????????????????????????????????????????????????????????????????????????????????????????????????????????????????????????????????????????????????????????????????????????????????????????????????????????????????????????????????????????????????????????????????????????????????????????????????????????????????????????????????????????????????????????????????????????????????????????????????????????????????????????????????????????????

aPrrdOC013131 11011001010101001010110000001101101011101110010110000001100101100101101010010010001101001101101000001101101000101001001101001011001101001001000000011011000110010001011101111010010110100110110011010010001100001000000100100001100000010010000000011110101100101010000001000000010000001000110000010100001000101010001011000010000000100101011000000010001000001000000000000010000010001000001000100000100010000000100010100101000100000000000010000000100000000001000010000010001000110111100011110110001101111110011111110101110111110011011001010000100101010101110110010101100100111010110011010101010101010101111110000110100000010110011010100000110110110000100001001000100000011101010111001100011010001110110100001101011000000100100001010000000100000001101101000000000100010001100000000000010000000000101000000000100100010000000001000000000000000000000000000000000000000????????????????????????????????????????????????????????????????????????????????????????????????????????????????????????????????????????????????????????????????????????????????????????????????????????????????????????????????????????????????????????????????????????????????????????????????????????????????????????????????????????????????????????????????????????????????????????????????????????????????????????????????????????10111010110101110101110110010101101010110111101000110011101101010100111001011001010110110001100000101111010001001100001000101101111011011000001000110000100110101000000010010101011010100010010001011100000100101001111001100001000101000000000110000000111001010001000100010010010100000000000000000000110010000000000000011000000100100010010000000000010000000000000000010010000000000000000010000000000001000000000100000000000000010000000000010000000000100001000000000000000000000000000000010011010110011011010010110011100011010001011011001101000110000100110011101000111110101000000001110011101000001000000000001001010011010011000000000000011000000001011000100000000001010000100000000011010000101010100000010010001001000000011010010000000100000100000000000000101000000001001000001000000000000000000111100001001000010000000000000000100000100000000000000100000010000000000000000000000000000000010101000100000010000??????????????????????????????????????????????????????????????????????????????????????????????????????????????????????????????????????????????????????????????????????????????????????????????????????????????????????????????????????????????????????????????????????????????????????????????????????????????????????????????????????????????????????????????????????????????????????????????????????????????????????????????????????????????????????????????????????????101111010101101011101010101100101011010110001001011010010110100010101101010101100000010010100001011001010011011110100000001000101000001110101001011001100000010000000001010100001000011001001001000101000001100001010000000000000010000001001000000010100001000001000000001010000000100001000110000000000000000000001001001000000000000000000000000000000000000000000000001000000000000000000000000000000000000000010001000000000000000000000000001000000000000011011110011011110111010101110111111101001101010101101101111001011101101100010001010101001111101110101000010101000010010101000101100010011000100101101000100110010100010100011000011011000001010000000001001001101010001101001001100001000000000011000001001010010000000000100000000100000000101001000100001010000000001000100000001000000000101000000000001010000000000000000000001000000000010000000001000000000000001000000000000000000000000000000000000000000000000001000000000000000011010101011010011000101010001010101011000101001101010001011010110101000100101001100011010100000010100110011010010101000100010010100100100110101101001100101101000010100001001000111001100001011100000001000101000000111000001010001000010000000000010010001110000010111001001000000000010001001000100100011100000001000000010101000000000000000000101000100100000001000000000000010100000000000000000000000000000100000000000010000000000???????????????????????????????????????????????????????????????????????????????????????????????????????????????????????????????????????????????????????????????????????????????????????????????????????????????????????????????????????????????????????????????????????????????????????????????????????????????????????????????????????????????????????????????????????????????????????????????????????????????????????????????????????????????1010111001001110101011011011010110110111001101011011011101010100001011011101001001101001000000100000001000110000000001000000101101001111000010011100010001100000101001110100000001100100000010100010000100000110000100001000000001000001000011010010000000000010000000000101100100001000000010001000000000000000000000000000010000000000000010000000000000010000010001000000010000001000000000000000000101010010100100010000010010100110101010000111111001000101011000010101000101111011000110110001110011101101101001010100011011001101000101100100000101100110100101111000101011000000000110001001010000111010010001010001000101000010000011011010110010100011001010110100000000010000000000100000000010100100001000000000000000010000000100000101000000000000000000010000011000100000101000000000000000000000010000000000100000010000000001000100000010000000000000000000001000000001000000000000000000010111010111101010110110010100111001100000101101010101011001010101001010010110110101100010101110100100010001100110001010010100110000110010100101000111011000001000001010100001000000000011000000000001000100000101000010000001000000100000100011000100000000000000000101101000100000011000010000100000000000000000000000000000001000000000000000100001001000000000000000000001000000000000100000000000000000000000000000000010000000000000101001010011011001100100111011101011001101001010111011100011101101001000001001111010101110100101111100100110100010110000100000000110100011000011011010000101000000001000100100101000000101000000000000001000011000100100000000010001000100000000000100000000000000000100000000000000000000100000011001000001000000000000000000000000000000001000000000000000000000000000000100000000000000000000000000100000001000000000000001000000000000000001100000000000000000000000010000000000000000000000000000000??????????????????????????????????????????????????????????????????????????????????????????????????????????????????????????????????????????????????????????????????????????????????????????????????????????????????????????????????????????????????????????????????????????????????????????????????????????????????????????????????????????????????????????????????????????????????????????????????????????????????????????????????????????????????????????????????????????????????????????????????????????????????????????????????????????????????????????????????????????????????????????????????????????????????????????????????????????????????????????????????????????????????????????????????????????????????????????????????????????????????????????????????????????????????????????????????????????????????????????????????????????????????????????????????????????????????????????????????????????????????????????????????????????????????????????????????????????????????????????????????????????????????????????????????????????????????????????????????????????????????????????????????????????????????????????????????????????????????????????????????????????????????????????????????????????????????????????????????????????????????????????????????????????????????????????????????????????????????????????????????????????????????????????????????????????????????????????????????????????????????????????????????????????????????????????????????????????????????????????????????????????????????????????????????????????????????????????????????????????????????????????????????????????????????????????????????????????????????????????????????????????????????????????????????????????????????????????????????????????????????????????????????????????????????????????????????????????????

aPrrdOC013132 11011001110101001010011001000101101011101110100100001001100101100101101010001010001011001101101100001001101010100001101100100011001001001011100000010011000011010011001101101000000110000100010011000010011100001001000100000000000000111010000000110000101100000000001001001000010000011000010000000100011000100010000011000000000000000001010000000010001010001000010110000010000000000000000000000000100001000000100001010001100100000001000010000000000000000000000010000010000101110111100011000111001101111100011111010101110101110001001001110000100011010101110110110101100110101000110001010101011010010001101111000110100000011010011010010000010000110000000001001000100000011100010101001100011000001100110100000011011000000100000001010001000000000001101100000000000100010001100000000000000000000000101000000000100100010000000001000000000000000000000000000000000000000????????????????????????????????????????????????????????????????????????????????????????????????????????????????????????????????????????????????????????????????????????????????????????????????????????????????????????????????????????????????????????????????????????????????????????????????????????????????????????????????????????????????????????????????????????????????????????????????????????????????????????????????????????10101000100100101101100101010100100010100101001100110110101101010100110001011001010110110001110000101111010001101000001000101101111011010000001000110000110101101000000010010101010010000010010000011100000100101001101001100001000101000000000111000000010011010001010101010010010100000000000000000000110000010000000000011000000100000010010000000000010000000000000000010000000000000000000010000000000001000000000100000000000000000000000000010000000000100000000000000000000000000000000000010011010110001011010010110011100011100011011011001101010110000100110010101000111111100010000001110011111000001000000000001000010000010010100000000000010000000001011000000000000001010000100000000011010000101010100000010110001001000000001010010010000010000000000000000000101000000001001000001000000000000000000000000001000100010000000000000000100000100000000000000100000000000000000000000000000000000000000101000101001010000??????????????????????????????????????????????????????????????????????????????????????????????????????????????????????????????????????????????????????????????????????????????????????????????????????????????????????????????????????????????????????????????????????????????????????????????????????????????????????????????????????????????????????????????????????????????????????????????????????????????????????????????????????????????????????????????????????????100111010100101011101010101100101011110110001001010010010110100010101101010101100000010010100000011000010010011111010000001000101000001110101001001001100000010000000000110100001000011001001001000101000001000101010000000000000010000001001000000010100101000001000000001010000000100001000110000010000000000000001000000000000000000000000000000000000000000000000000001000000000000000000000000100000000000001100000000000000000000000000000001000000000000010011100011011110111010101110110111101001101010100101101111011011101001100010001011101011001101010101000110101010010010101000100100010100100100101101000100110010100010100011000001011000001100000000001000101101010001101001101100001000000000011000001001000110000000000100000000100000000101001000100000100100000001000100000000000000000001000000000000001000000000000000001010000000000000000000001000000000000001000000000000000000000000000000000000000000000000001000000000000000010110101111010001100101111011010101101000101001101010001011010110101000110100001000011101000000010100110111010010101011110010010100100100110101100101100110101000100010001001000100101100010001100000001000100000000111000000010001000011000000000010000001110000010111001010000000000010001001000100100101100000010000000010001000000000000000000101000000100000001000000000000010100000000000000000000000000000100000000000000000000000???????????????????????????????????????????????????????????????????????????????????????????????????????????????????????????????????????????????????????????????????????????????????????????????????????????????????????????????????????????????????????????????????????????????????????????????????????????????????????????????????????????????????????????????????????????????????????????????????????????????????????????????????????????????1010111001101110101011011011010110110101001101011011011101010100001011011101001001101101000000100000001000100000000001000000101001001011000010011010010001100000101001110100000001100100000010100010000100000110000100001000000001000000100001000010000000000010000000000101100010001000000010001000000000000000000000000000000000000000000010000000000000001000010001000000010000000100000000000000000111101010100100010000010010100110101010000110111101100111011100010101100100011011000110010001110011001101100001010100100011011000000101100100000111011110100101101101010011000000010111001001010000111010010011010001000101000011000010011010110010100001001010010100000000010000000000100000000010100010001000100000000000010000000101000100000000000000000000010000010000100000101000000000000000000000010000000101000000010000000001000100000010000000000000000000001000000001000000000000000000000001010011101001010110010100111101100000101001010101011100110101000000100000110001101010100100001110010001100111001010010010110000100010100101001010001000001000001000100001000000000010000000000001000010000010000000010001000000100000000111000100000000000000000001000100000000001000000000000000000000000000000000001000001000000000000000100000001010000000000000000010000000000000100000000000000000000000000000000010000000000000101001010011010001100100111011101001001101001100011011000011101110001000001001111010101110100101111100100111000010110000100000000110001001000010010100000010000000101000110100101000000001000000000000001000010000100110000000000001000100000000000100000000000000000100000000000000000000100000011000000001000000000000000000000000000000001000000000000000000000000000000100000000000000000000000000000000001000000000000001000000000000000001100000000000000000000000010000000000000000000000000000000??????????????????????????????????????????????????????????????????????????????????????????????????????????????????????????????????????????????????????????????????????????????????????????????????????????????????????????????????????????????????????????????????????????????????????????????????????????????????????????????????????????????????????????????????????????????????????????????????????????????????????????????????????????????????????????????????????????????????????????????????????????????????????????????????????????????????????????????????????????????????????????????????????????????????????????????????????????????????????????????????????????????????????????????????????????????????????????????????????????????????????????????????????????????????????????????????????????????????????????????????????????????????????????????????????????????????????????????????????????????????????????????????????????????????????????????????????????????????????????????????????????????????????????????????????????????????????????????????????????????????????????????????????????????????????????????????????????????????????????????????????????????????????????????????????????????????????????????????????????????????????????????????????????????????????????????????????????????????????????????????????????????????????????????????????????????????????????????????????????????????????????????????????????????????????????????????????????????????????????????????????????????????????????????????????????????????????????????????????????????????????????????????????????????????????????????????????????????????????????????????????????????????????????????????????????????????????????????????????????????????????????????????????????????????????????????????????????????

aPrrdOC013134 11011001110101001010011001010101111011101110010100001001110101101010101010001010001010001101101110001001101010101001111011000011010101001011001000011111100110010101000101111010110110100010010110000010001110001000000101100001100000110001000001001000100110100000100001010000010000011000010101001100001000100010000101010000000000000101010000000010001010010010100101100110000100001000000000100000110001000000010000100000010100000001100010000000000000000010000001000010000100010111100011010111011101111100011111010100110110111001011001000001010101010100110110110101100110111011111001010101011010010001101111000111100000000011011010010000110000110000000001001000100000011100010111001100011000001100110100100101011000000110100001010001000100000001100110000000000100010001100000000000010000000000101000000000100100010000000011000000000000000000000000000000000000000????????????????????????????????????????????????????????????????????????????????????????????????????????????????????????????????????????????????????????????????????????????????????????????????????????????????????????????????????????????????????????????????????????????????????????????????????????????????????????????????????????????????????????????????????????????????????????????????????????????????????????????????????????10111011101101111101111111010000100010100111101100111111101101111100111001011001010110110001100000001111010001001100001000101101111011011000101111111100100111101000000011010101010010100010010000010110000100101001101101100001101100000000000011110000010001011001010101100010010100000000000000000000100000010000000000011000000100010100000000000000010000000000000000010010000000000000000001000000000001000000000100000000000000000000000000010000000000010000000000000000000000000000000000010011100110011011010010110011100011010011011011001101010110000100110011101000111110100010000001110011101000001000000000001000010011010010100000000000010000000000011000100000000001010000100000000001010000101000100000010010001001000000010010010010000000000000000000000000101000000000000000000000000000000000011000000001000100010000000000000000100000100000000000000100000000000000000000000000000000000000010101000100101010000??????????????????????????????????????????????????????????????????????????????????????????????????????????????????????????????????????????????????????????????????????????????????????????????????????????????????????????????????????????????????????????????????????????????????????????????????????????????????????????????????????????????????????????????????????????????????????????????????????????????????????????????????????????????????????????????????????????100111010101101010101010101100101011010100001001010010010010100010101101010101100000010010100001001001000010011110010000001000101000001110101001001001100000010000000000010100001000011000101000000101000001101001010000000000000010000001001000000010100101000000000000000010000000100001000100000000000000001000001001001000000000000000000000000000000000000000000000001000000000000000000000000000000000000001011001000000000000000000000000001000000000000010011101010101110111010101111010110100000101010100101101101001010010101010010001010110001011001010101000110101010010001101000101100010101100100101101000100110010100010100011000101011000001010000000000001001101010001101001101100001000000000010000001000010010000000000100000000100000000101001000100001010000000001001000000010000000000010000000010000001000000000000000001001000000000010000000001000000000000001000000000000000000000000000000000000000000000000000000000000000000011010101111010001000101111001010101101000101001101010001011001010110000100101001000011101010000010100100111010010101001110010010101000100110100101011000100101001100010101001000110101100001101100100001000101000000010100000010001000010000000000010000000110000010111001010000000000010001001000100100011100000010000000000001000000000001000000101000100100000001000000000000010100000000000000000000000000000100000000000001000000000???????????????????????????????????????????????????????????????????????????????????????????????????????????????????????????????????????????????????????????????????????????????????????????????????????????????????????????????????????????????????????????????????????????????????????????????????????????????????????????????????????????????????????????????????????????????????????????????????????????????????????????????????????????????1010111001101110101110011011100110110101001111011001011101010100001011011101001001101001000000100000001000101000000001000000101001001111000010011100010001100000101001110100000001100100000010100001000100000100000100001000000001000000100011000010000000000010000000000101100000001000000010001000000000000000000000000000000010000000000010000000000000010000010001000000010000000100000000000000000111011010100100010000010010100110101010000111011001000101011000010101000000000010000110010001100011001101000101010000000011001001001101000100000101100110100101111000000001000000010110001001000000111010010001010000000101000010000011011010110010100001001000110100000000000000000000100000000010100010001000100000000000010000000010000011000000000000010000010000011000100000101000000000000000000000000000000000000000010000000001000100000010000000000000000000001000000001000000000000000000000011010011100001010010010100111001100000101001010101001000011000010000100100111011001000100100000100010001110110001010001100100000100010100101000000101000001000001000100001000000000010000000000001000010000010000010000001000010100000100101000100000000000000000001000000100000010000010000010000000000000000000000000000001000000000000000100001001010000000000000000010000000000000010000000000000000000000000000000010000000000000101001010011010101100100111011101011101101001000111011100011101010000000001001111000101110100101111100100110000010110000010000000110101001000010010110000010000100001000100100101000000101000000000000001000011000100100000000000001000100000000000100000000000000000100000000000000000000100000011001000001000000000000000000000000000000001000000000000000000000000000000100000000000000000000000000100000001000000000000001000000000000000001100000000000000000000000010000000000000000000000000000000??????????????????????????????????????????????????????????????????????????????????????????????????????????????????????????????????????????????????????????????????????????????????????????????????????????????????????????????????????????????????????????????????????????????????????????????????????????????????????????????????????????????????????????????????????????????????????????????????????????????????????????????????????????????????????????????????????????????????????????????????????????????????????????????????????????????????????????????????????????????????????????????????????????????????????????????????????????????????????????????????????????????????????????????????????????????????????????????????????????????????????????????????????????????????????????????????????????????????????????????????????????????????????????????????????????????????????????????????????????????????????????????????????????????????????????????????????????????????????????????????????????????????????????????????????????????????????????????????????????????????????????????????????????????????????????????????????????????????????????????????????????????????????????????????????????????????????????????????????????????????????????????????????????????????????????????????????????????????????????????????????????????????????????????????????????????????????????????????????????????????????????????????????????????????????????????????????????????????????????????????????????????????????????????????????????????????????????????????????????????????????????????????????????????????????????????????????????????????????????????????????????????????????????????????????????????????????????????????????????????????????????????????????????????????????????????????????????????

aCyaafrCH010245 11011101010101110110110101001101101011101111010110111001110101111101111011101100011011101101011001001001111010101001011101011001001101001011001101010111000111101011001110101001110110100110100010011011001010000101000101111001000001011000011000001000100100101100000001000000010000000010010000000100001000110010000001000000000000001001010000010001001001001000000000000110000010000000000100100000010001000000010000001000000010000000000001000000001001000000000001000000000010000100000000000110001101001000000011000000100000010001001001000000010101010100010110101000000100101000010000000001010010000000101010000110100000000001000010000000010000010000000001000000000000011000000100000100001000000100000100000000011000000000000001010000000000000000100000000000000000100001000000000000000000000000100100000000000000000000000001000000000000000000000000000000000000000????????????????????????????????????????????????????????????????????????????????????????????????????????????????????????????????????????????????????????????????????????????????????????????????????????????????????????????????????????????????????????????????????????????????????????????????????????????????????????????????????????????????????????????????????????????????????????????????????????????????????????????????????????10110000000001110101000111010000100010100101010000100100000100010100100001011000010100000000000000000010000000000000000000001000000000010000000000110000100001001000000010100000010000000000000000000100000100000000101000000000000000000000000000000000010000010001000001000000000100000000000000000100010000000000000000000000000100000000000000000000010000000000000000000000000000000000000000000000000000000000000000000000000000000000000000000000000000000000000000000000000000000000000000000001000000000000000000000001000000000000000000000000000000000000000000000000000000000000000000000000000000000000000000000000000000000000000000000000000000000000000000000000000000000000000000000000000000000000000000000000000000000000000000000000000000000000000000000000000000000000000000000000000000000000000000000000000000000000000000000000000000000000000000000000000000000000000000000000000000000000000000000000000000000??????????????????????????????????????????????????????????????????????????????????????????????????????????????????????????????????????????????????????????????????????????????????????????????????????????????????????????????????????????????????????????????????????????????????????????????????????????????????????????????????????????????????????????????????????????????????????????????????????????????????????????????????????????????????????????????????????????100100010100100000101010100100001001000100010001010000010000101010000100000101000000010010100100000000000000010000001000101000100000000110000000000001000000010000000000010000000000010000000000000100000000000001010000000000000000000000001000000010100000000000000000000000000000000001000000000000000000000000001000000000000000000000000000000000000000000000000000000000000000000000000000000000000000000000000000000000000000000000000000000000000000000010001110010011110110010001100010110100000001010100100001101000010000000000010001010000101101001000001000000010000100000000000000100000000100100000101000000000010000010100000000001000000000000000000000000000101000001100000000000000000000000000000000000000000000000000000000000000000000000000000000000000000000000000000000000000000000000000000000000000000000000000000000000000000000000000000000000000000000000000000000000000000000000000000000000000000000000000000000000000000000001011111010000000000010001000000000000000001001101000000000010000000100000001000011101000000000000000010100000000001010000010010000100000000000011000100010000010000001100000000000000000000010000001000100000000000000000100000000000000000000000000000000000010000001000000100000000000000000000000000000000000000000000000000000000000000000000000000000000001000000000000001010000000000000000000000000000000000000000000000000000???????????????????????????????????????????????????????????????????????????????????????????????????????????????????????????????????????????????????????????????????????????????????????????????????????????????????????????????????????????????????????????????????????????????????????????????????????????????????????????????????????????????????????????????????????????????????????????????????????????????????????????????????????????????????????????????????????????????????????????????????????????????????????????????????????????????????????????????????????????????????????????????????????????????????????????????????????????????????????????????????????????????????????????????????????????????????????????????????????????????????????????????????????????????????????????????????????????????????????????????????????????????????????00000000010010000000010000101010100001001100101000111100000010100111010000100100001010110001110011100000111000100001000011000000000000000000000000000000000000000000000000000000000000010000000000000000000000000000000000000000000000000000000000000000000000000000000000000000000000000000000000000000000000000000000000000000000000000000000000000000000000000000000000000000000000000000000000000000000000000000000000000000000000000000000000000000000000000000000000000000000000000000000000000000010011110001010000010100011001000000101000010101001000110000000000000000110001011000000000000100010001010100001010000010110000100000000001000010000000000000001000000001000000000000000000000001000010000010000000000001000000100000000001100000000000000000000001000000000000000000000000000000000000000000000000000000001000000000000000100000001000000000000000000001000000000000010000000000000000000000000000000000100000000000101001000111011001110100011011010000000111001000001011100011101100010000000000111000101010100101111010100010000001010000000001000110000001000010010100000011000000000001100000001000000001000000000000000000010000100100000000000001000100000000000100000000000000000000000000000000000000100000011000000001000000000000000000000000000000001000000000000000000000000000000011000000000000000000000000000000001000000000000000000000000000000000000000000000000000000000000000000000000000000000000000000???????????????????????????????????????????????????????????????????????????????????????????????????????????????????????????????????????????????????????????????????????????????????????????????????????????????????????????????????????????????????????????????????????????????????????????????????????????????????????????????????????????????????????????????????????????????????????????????????????????????????????????????????????????????????????????????????1101011100001111010011010110000110000111101101111101001100001100001110001100000000000000000000101100000000000000000000000000000000000010010000000001010000000001000010000000001100001000010000010000010010001001000010000001000000000000010010000000001000010000000000010000000000000000000000000001000000001000000000000000000000000000100000000000000000000000000000000000000000000000000000000100000000000000000000000000000000000000000000000000000000000000000000000000000000000001000011000001010000000000000000100000000010000001011000000011000000000000000000100000000000000000000000001000000000000100000000001000000100000010000000000000000000000000000000000000000000000000000000000000000000000000000000000000000000000000000000000000000000000000000000000000000000000000000000000000000000000000000000000000000000000000000????????????????????????????????????????????????????????????????????????????????????????????????????????????????????????????????????????????????????????????????????????????????????????????????????????????????????????????????????????????????????????????????????????????????????????????????????????????????????????????????????????????????????????????????????????????????????????????????????????????????????????????????????????????????????????????????????????????????????????????????

aCyaafrCH010246 11011101010101010111011011010101101011101111010110111001110101101101111010101100001011101001011010001001111010101001001100101011001101001011011001011111000111101011000101101001110110100110100010011011001010000001100101111001001001011000010000001000100100111100000001000000010100000010010010000100011000110010000001010001000000101001000000000001001001001000000000000110000010000000000100000000010001000000010000101000000010000000000001000000000000100000000001000000000010000100100000000110001101101000000011000000100000010001001001000000010011010100010110100000000100101000010000000001010010000000101000000110100000000001000000000000010000010000000001000000100000011000000100000100001000000100000100000000011000000000000000000000000000000000100000000000000000100001000000000000000000000000000100000000000000000000000000000000000000000000000000000000000000000????????????????????????????????????????????????????????????????????????????????????????????????????????????????????????????????????????????????????????????????????????????????????????????????????????????????????????????????????????????????????????????????????????????????????????????????????????????????????????????????????????????????????????????????????????????????????????????????????????????????????????????????????????10110000000001100101110111010000000010100101000000100110001100010100100001011001010100010100000000001010000000000000000000001000000000010000000000110000100001101000000000100000010010000000000000000100000100000000101000000000000000000000100000000000010000010001000001010000000100000000000000000100010000000000000000000000000100010000000000000000010000000000000000000000000000000000000001000000000000000000000000000000000000000000000000000000000000000000000000000000000000000000000000000001000000000000000000000001000000000000010000000000000000000000000000000000000000000000000000000000000000000000000000000000000000000000000000000000000000000000000000000000000000000000000000000000000000000000000000000000000000000000000000000000000000000000000000000000000000000000000000000000000000000000000000000000000000000000000000000000000000000000000000000000000000000000000000000000000000000000000000000000000000000??????????????????????????????????????????????????????????????????????????????????????????????????????????????????????????????????????????????????????????????????????????????????????????????????????????????????????????????????????????????????????????????????????????????????????????????????????????????????????????????????????????????????????????????????????????????????????????????????????????????????????????????????????????????????????????????????????????100100010100100000101010100100001011000100000001010000000010100010001100000101100001010010100100000000000000010100000000100000100000000110000000000001000000010000000000010000000000010000001000000100000000000001010000000000000000000000001000000010000000000000000000000000000000000001000000000000000000001000001000000000000000000000000000000000000000000000000000000000000000000000000000000000000000000000000000000000000000000000000000000000000000000000000100010000100111010001000010110100000001010001100000100000010000000000010000100000000000001000001000000000100100000000000000100000000100100000000000000000010000000100000000000000000000000000000000000000100000001100000000000000000000000000000000000000000000000000000000000000000000000000000000000000000000000000000000000000000000000000000000000000000000000000000000000000000000000000000000000000000000000000000000000000000000000000000000000000000000000000000000000000000000011011111010001000000010001000000000000000001001101000000000010000000100000001000011111000000000000000010100000000001110000010010000100000000000011000100000000101000001000000000000000000000010000001000100000000000000000100000000000000000000000000000000000010000001000000000000000000000000000000000000000000000000000000000000000000000000000000000000000001000000000000101000000000000000000000000000000100000000000000000000000???????????????????????????????????????????????????????????????????????????????????????????????????????????????????????????????????????????????????????????????????????????????????????????????????????????????????????????????????????????????????????????????????????????????????????????????????????????????????????????????????????????????????????????????????????????????????????????????????????????????????????????????????????????????????????????????????????????????????????????????????????????????????????????????????????????????????????????????????????????????????????????????????????????????????????????????????????????????????????????????????????????????????????????????????????????????????????????????????????????????????????????????????????????????????????????????????????????????????????????????????????????????????????01011010000100010000011010100010101010000001011001000100001000000001000000000010000000010000000010000001000000000000000000000000000000000000000000000000000000000000000000000000000000000000000000000000000000000000000000000000000000000000000000000000000000000000000000000000000000000000000000000000000000000000000000000000000000000000000000000000000000000000000000000000001000000000000000000000000000000000000000000000000000000000000000000000000000000000000000000000000000000000000000000000010011100001010000010100011001000000101001011101001000110000000000000000110000001000000000000100010001010100001010000010110000000000000001000010000000000000001001000001000000000000000000000001000010000000000000000001000000100000000010000000000000000000000001000000000000010000000000000000000000000000000000000000001000000000000000100000001000000000000000000001000000000000010000000000000000000000000000000000100000000000101000000010000001100100001011100000000111001000001011100011101000000000000000111000101010100101111010100010000011010000000000000110000001000110010000000010000010000000100000001000000001000000000000000000010000100100000000000001000100000000000100000000000000000000000000000000000000100000011000000001000000000000000000000000000000001000000000000000000000000000000100000000000000000000000000000000001000000000000001000000000000000000000000000000000000000000000000000000000000000000000000000???????????????????????????????????????????????????????????????????????????????????????????????????????????????????????????????????????????????????????????????????????????????????????????????????????????????????????????????????????????????????????????????????????????????????????????????????????????????????????????????????????????????????????????????????????????????????????????????????????????????????????????????????????????????????????????????????1101011100001101010011000100000100000110101101011001001000001100001100001000000000000000000000101100000000000000000000000000000000000010010000000001010000000001001000000000001100001000010000010000010010001001000010000001000000000000000010000000001000010000000000010000000000000000000000000001000000000000000000000000000000000000100000000000000000000000000000000000000000000000000000000100000000000000000000000000000000000000000000000000000000000000000000000000000000000001000011000000001000000000000000100001000010000001011000000010000000000000000000000000000000000001000000000000000000000100000000000000000100000010000000000000000000000000000000000000000000000000000000000000000000000000000000000000000000000000000000000000000000000000000000000000000000000000000000000000000000000000000000000000000000000000000????????????????????????????????????????????????????????????????????????????????????????????????????????????????????????????????????????????????????????????????????????????????????????????????????????????????????????????????????????????????????????????????????????????????????????????????????????????????????????????????????????????????????????????????????????????????????????????????????????????????????????????????????????????????????????????????????????????????????????????????

aCyaafrCH010681 11011101110101101011011101010101111011101110010100111001110101111101101010111100011011000101011001001001111010101001101100101011001101001001100101101100000110001011001100101001110110000110100010101011001100000001100101111001001000111000101000001000100100101100000001000000010000000010001010000100001000100010000001000000000000001001000000010001001001001000000000000110000010000000000100000000010001000000010000100000000010000000000001000000000001000000000001000000000010000000100000000110001101001000000000000000000000010000001001000000000001010100000000100000000100000000010000000001010010000000000000000000000000000000000000000000000000000000000000000000000000011000000100000000000000000000000000000000001000000000000000000000000000000000000000000000000000000000000000000000000000000000000000000000000000000000000000000000000000000000000000000000000000000????????????????????????????????????????????????????????????????????????????????????????????????????????????????????????????????????????????????????????????????????????????????????????????????????????????????????????????????????????????????????????????????????????????????????????????????????????????????????????????????????????????????????????????????????????????????????????????????????????????????????????????????????????10110000000010101101001111010000100010100101000000100010001100010100110001011001010100010000000000001010000000000000000000101000000000010000000000110000100000001000000001000000010010000000000000000100000100000010101000000000000000000000000000000000010000010001010000010000000100000000000000000100000000010000000000001000000100000000000000000000010000000000000000000000000000000000000001000000000000000000000000000000000000000000000000000000000000000000000000000000000000000000000000000001000000000000000000000001000000000000000000000000000000000000000000000000000000000000000000000000000000000000000000000000000000000000000000000000000000000000000000000000000000000000000000000000000000000000000000000000000000000000000000000000000000000000000000000000000000000000000000000000000000000000000000000000000000000000000000000000000000000000000000000000000000000000000000000000000000000000000000000000000000000??????????????????????????????????????????????????????????????????????????????????????????????????????????????????????????????????????????????????????????????????????????????????????????????????????????????????????????????????????????????????????????????????????????????????????????????????????????????????????????????????????????????????????????????????????????????????????????????????????????????????????????????????????????????????????????????????????????100100010100100000101010100100001001000100000001010000000010100010001110000101100000010010100000000000000010010100000000011000100000000110000000000001000000000000000000010000000000010000000000000100000000000001010000000000000000000000001000000010100000000000000000000000000000000001000000000000000000001000001000000000000000000000000000000000000000000000000000000000000000000000000000000000000000000000000000000000000000000000000000000000000000000010001110010011110110010001100010110100000001010100101001101000010001001000010001010000001001101000001000000001000100000000000000100000000100100000101000000000010000010100000000001000000000000000000000000000100010001100000000000000000001000000000000000000000000000000000000000000000000000000000000000000000000000000000000000000000000000000000000000000000000000000000000000000000000000000000000000000000000000000000000000000000000000000000000000000000000000000000000000000000000101001111010000000000010001000000000000000001001001000000000000000000100000001000011101000000000000010010100000000001000000010010000010000000000011000100010000100000001000000000000000000000010000001000100000000000000000010000000000000000000000000000000000010000001000000000000000000000000000000000000000000000000000100000000000000000000000000000000000001000000000000001010000000000000000000000000000100000000000000000000000???????????????????????????????????????????????????????????????????????????????????????????????????????????????????????????????????????????????????????????????????????????????????????????????????????????????????????????????????????????????????????????????????????????????????????????????????????????????????????????????????????????????????????????????????????????????????????????????????????????????????????????????????????????????????????????????????????????????????????????????????????????????????????????????????????????????????????????????????????????????????????????????????????????????????????????????????????????????????????????????????????????????????????????????????????????????????????????????????????????????????????????????????????????????????????????????????????????????????????????????????????????????????????01101010000010010000010000100010101010000000010001000100001000000001000000000000000000010000000010000000000000000000000000000000000000000000000000000000000000000000000000000000000000000000000000000000000000000000000000000000000000000000000000000000000000000000000000000000000000000000000000000000000000000000000000000000000000000000000000000000000000000000000000000000000000000000000000000000000000000000000000000000000000000000000000000000000000000000000000000000000000000000000000000000010011101001010000010100011001000000101001010101001000110000000000000000110001001000000000000100010001010100001010000010110000100000000001000010000000000000001001000001000000000000000000000001000010000010000000000001000000100000000001000000000000000000000001000000000000001000000000000000000000000000000000001000001000000000000000100000001000000000000000000001000000000000010000000000000000000000000000000000100000000000101001000010011011100110011011011000000111000000001011100011111000010000000000101000101010100101111010100010000010010000000001000110000001000010010100000010000000000000100000011000000001000000000000000000010000100100000000000001000100000000000100000000000000000000000000000000000000100000011000000001000000000000000000000000000000001000000000000000000000000000000100000000000000000000000000000000001000000000000000010000000000000000000000000000000000000000000000000000000000000000000000000???????????????????????????????????????????????????????????????????????????????????????????????????????????????????????????????????????????????????????????????????????????????????????????????????????????????????????????????????????????????????????????????????????????????????????????????????????????????????????????????????????????????????????????????????????????????????????????????????????????????????????????????????????????????????????????????????1100011100001101000011000110000110000110011101111101001100001100001110001000000000000000000000101100000000000000000000000000000000000010100000000010100000000000000000000000011000001000010000010000010010000100100001000001000000000000010010000000001000001000000000001000000000000000000000000001000000000000000000000000000000000001000000000000000000000000000000000000000000000000000000000100000000000000000000000000000000000000000000000000000000000000001000000000000000000001000010000001010000000100000000100000000010000001011000000011000000000000000000000000000000000000000000000000000000000000000000000000000000000000000010000000000000000000000000000000000000000000000000000000000000000000000000000000000000000000000000000000000000000000000000000000000000000000000000000000000000000000000000000000000000000000000????????????????????????????????????????????????????????????????????????????????????????????????????????????????????????????????????????????????????????????????????????????????????????????????????????????????????????????????????????????????????????????????????????????????????????????????????????????????????????????????????????????????????????????????????????????????????????????????????????????????????????????????????????????????????????????????????????????????????????????????

aMeauOP010025 11011101110101010111011001010101111011101111010110111001110101101101101010001101001111001101100100001001011011101001101100100011001101001001000110011101010110011011101110111001100110000110100011000011001010010000001110100001001100001001010000000000100100100000100001000001011100001010001010001100001100100010000011010010010000000000000001010001001001001001110000000010000010101000010000000000010001000010010000000000000110000001000001010000100000100000000000100000000010010111110011000110101101111101011111010101110110111001101101100001010011010100110110110101100110111001110011010001010011010100101111000110100000011010001010010010011100110100101001001000110001011110000111011101111000011110110100000001111010000100100101010001000010100001101010000000001000010001000001001000010000000010100100000000000100000000100011000000000001000001000000000010000000000001010001001100011001000101110100001100101000000000010001011000100010100100000010000100010000000001000000000000001000000000000000000010000000000000000000000000010100000000000000010000010000010000000100000000000000000000010000000000000000000001000000000001000000000000000000000000000000000000000000000000000000000000000000000000000000000000000000000000000000000000000000000000000000000000000000000000000000000000000000000000010110100010101101101010101010101101010101101101010101110101110010101101001011101010100110001100100001011010001001100101010101101011011010010011011110110100100111101110001000100010010110100010000011101000101101010101101110010000100100001000000000000111000010001010101010010010100000001010000100100100000000000010000001000000101001000000000000001010000000000000000010000000000000010000001000000000101000000000000001000000001000000000000000000000000000000000000000010000000100000000000010011001010001001010010010001000001010101011011001101000101000100100010100100100100100110000101110010010000011000101001001000010000000000100001000000110100010001111001011100000100111001100001000000010010101010100000100110001000010000000100010010011001000000000010000010101000000001000000000000100000000000000000010001000000000000000000000100100000000100000000000100000000000000000000000000000000000100001000000100001010000??????????????????????????????????????????????????????????????????????????????????????????????????????????????????????????????????????????????????????????????????????????????????????????????????????????????????????????????????????????????????????????????????????????????????????????????????????????????????????????????????????????????????????????????????????????????????????????????????????????????????????????????????????????????????????????????????????????101101010100101011101010100100001011110101001001110010010010100110101111011101101000101110100010011001010010011110111000011011101001100110001101001101100110011000101001010100001000011001000100001101100010100001010000100000000010000001001000000010100101000000000000000010010000100001000000000000000000111000001000000000000000000000000000000000000000000000100000001010000000000000000000000000000000010000000100000000000000000000000000000100000000000011011110011010110111010101101011111101010101010110101101101011011001101100010011011110100111101011101000110110101010001101011010101011101101010101101000100111001100010010111100111111010000010110001000001101101011101100101001110011001000001010000100000010110011000000100000000100000000111001000100001110100000001110100000000000011001101000111000000101010000000000000010100000100000000000000101000000000000000100000000000000000000000100000000000000000000000000000000000100000010110111111111011100101111011010101101100101101101001001101010111101100101101001100011011010010010011110110100011010001010100110010100110110101100101000110100100010011100100110110111000001000011100001000101010010000100010010000000010010000001000000000101001110111001001010000000010000101000100010011010000110000000000101000000000000010000011000000000000100100000000010101010000000000000000000000000000010000000000000000000000???????????????????????????????????????????????????????????????????????????????????????????????????????????????????????????????????????????????????????????????????????????????????????????????????????????????????????????????????????????????????????????????????????????????????????????????????????????????????????????????????????????????????????????????????????????????????????????????????????????????????????????????????????????????1010111001101110101110011011100110101101001100111011011111010101011111001101001101101101011000101101001100100100010001100000101101010001001010011110010011110000101001110110100101010100000010100011000100000100000100001001000101100010000101000010000000010010000000001101100010010000000010101000000000001000000000000000010010000000000010000000000000001000010000000000010000000000000000000000000101101011010100010101011100100110101011000111011101000111011100011101000100010011000100110001100011011111100101010010010001000101000110101100010101010110100101110000100011100000000110010010011000010010110010010000000100000010000101010010110010100010000000000000000000000000000000100100000000100000000001000000000001011010000000000000000000000000000000001000010000000000000100000000000000000010000010000000100000000000000000000100000010000000001000000000000000000000000000000000000000010010010111101010110101010100111001111101101001010111011000110101001000101110111001001100101010100100010010110100001010001001110000100001110001110110101000010100111001000101001010000010010001000011001010000010001010100001110010100100000101000101000000000000101001000000100000011000010000010000100000000000000000000000001000000000000000100000001000000000000000000001000000000000010000000000000000000000000000000000100000000000101001010011010101100100111011101011101111001000111011110011101101011100001001111010101110100111111011100111101011110010010101000110100011110010010010001010010010001100110100101010000011000001001100001000011010100100000000010101011100110001000100000000000000000100010001000001000000100000011001000101000000000000000000000010000000001000000000000000000000100000000100000000000000000000010000000000001000000000000000110000000000000000000100000000000000000000010000000000100000000000000000000???????????????????????????????????????????????????????????????????????????????????????????????????????????????????????????????????????????????????????????????????????????????????????????????????????????????????????????????????????????????????????????????????????????????????????????????????????????????????????????????????????????????????????????????????????????????????????????????????????????????????????????????????????????????????????????????????110101111101110101001001011010111010011101111001011010110100110000110010110100101000110001000011011000010101010000110101000101000000101100101001100101001110010010111001000100101011100001100011010000101000100111100100000100010000000001001000000001010001000010000001010000000000001000000000000100000010100000000000000000000000000001000001000010000000000100000000000000000000000001000000010000100000000000000000000000001010000000000000010000000000100000000000000100000001???????????????????????????????????????????????????????????????????????????????????????????????????????????????????????????????????????????????????????????????????????????????????????????????????????????????????????????????????????????????????????????????????????????????????????????????????????????????????????????????????????????????????????????????????????????????????????????????????????????????????????????????????????????????????????????????????????????????????????????????????????????????????????????????????????????????????????????????????????????????????????????????????????????????????????????????????????????????????????????????????????????????????????????????????????????????????????????????????????????????????????????????????????????????????????????????????????????????????????????????????????????????????????

aMeauOP010026 11011101010101011011011010010101101011010111010110010001110101101101101010001010001011101101101001001001011010101001111100101011001101001001010010101111010111001101100100101000110110000110100010010011001010011000100100100001100100010010010000110000100100100001000001001100010100011010010000000100001000100010000001010010010000001001100001000001001001001000100000000010000001011000001001001001010001000010010000000000000010000000000001010000100000000000000001000000100010110111110011100111101101111101011111011111110111011001001001000000100011010101110110111001100110101001010011010101010010010100101111000110100000000110001010010010010000110100101001001000100001011110000101010100111000010100110100100001011010001010000101010000000100000001101010000000000000010001000001001000010000000100100100000000000100000000110001000000000000000000000010101010000000000000110001001100011001100101110100001000101000010001111001001000100110100000000010000010000000010001000010010000001000000000000000000010000000000000000000000000011100000000000000100000010000010000000100000000000000000000000000000000000000000001000000000001000001000000000000000000000000000000000000100000000000001000000000000000000000001000000000000000000000000000000000000000000000000000000000000000000000000000000000001000010111000010101110101101101010100111010101101010000100110101100010100101001001101010110110100100100001011010010001000101100101101111010011011001010110100100110101000000011010100010010000101010000001101000100001010101000110001000101000001000000001000011010010001010101010010000100000010000100100000100000000000010000001000000101000000000000000000011000000000000100000000000100000010000001000000000001000000000000000000000001000000000000000000000000101010000000000010000000100000000000010011000100001011010010010001000001000000010001001101000100000100100010100100100100100100000001110010010000101000100000101000010010100001001001000001011000101001011010010100000101011010100001000000010110101010100001000110000001010000001000010010101001000000000010000011101000000000001000001000000000000000000000000001000000000000000000001100100000000100000000001000000000000000010000000010000000000100010000000100001010000??????????????????????????????????????????????????????????????????????????????????????????????????????????????????????????????????????????????????????????????????????????????????????????????????????????????????????????????????????????????????????????????????????????????????????????????????????????????????????????????????????????????????????????????????????????????????????????????????????????????????????????????????????????????????????????????????????????101110010111101011101010100100101011010110001001010010010110101110101111010101101001001010100010111000110011011100111000011010101000001111001001001011010100111000101001010000011000011101101110001101000010100101010000001000000000100101001000000010100101010001000000010010100010100101000000000100000000001000001100001000000000000000000000010000000000000000010000001001000000100000100000000000000000001000000100000000000000000000000000000100000000000011010100010010110111010001000010110100010101010110101101101011010001001101010001010110101111101011101000111010101011001110011011101110101101100101101010100110010100010100111110101110010000001110001000001101101010011101001001110101001000001110000101000001100011000000100000000100000000101001000100001101100000011010100000000000101110001000111000000101000000000100000001001000100000001000000001000000000000000100000000000000000000000100000000000100000000000000000000000100000010110111111011011000101111011010101011000101101101001001101010111101100101101001100011011010010010100110110100011100001010100110101001101110111100101000101101000010011101000010111011000001000011100001000101010000000100010110000000010010000001000000000110001110111001001000000000010000101000100010011010000110000000000001000000000000000000001000100000000101000100000010000100000000000000000000000000000010000000000000000000000???????????????????????????????????????????????????????????????????????????????????????????????????????????????????????????????????????????????????????????????????????????????????????????????????????????????????????????????????????????????????????????????????????????????????????????????????????????????????????????????????????????????????????????????????????????????????????????????????????????????????????????????????????????????1010111001101110101011011011100110100101101100111011011101010000011111001101101101101101010000100001001110100101010001100000101001001001000010011100011001000000101001110110001011110000000010100010000100000100000100001001000001101010000101000010000000000010000000010101100010000000000010001000000000001000000000000000010001000000000010000000010000000100010001000000010000000000000000000000000101101010110110011001111010100110101011000110111101001111011110011101110101011111000111111101100011001111101001110000010001001001000110101100000101010110100101111000100001000000000110100010010000110010010100011000000100000011000110001010110010100010000000000000000000000000000001100000001000110000000000000000000000011001000010000000000000000000010000001000010000000000001000000000000000000000010000000001000000010000000000000100000010000000000000000000000000000000000000000000000000010011010111100101011010010100111001100001101101010101111110110101010000101110111101011101101000010101011001010110100111001010110000111010110001100111001000010100101001000101001011000010000001000011001011000010001010100001010010100100000110000100001000000000001001000001001000011100010000010010100000000000000000001000001000000000000000100000001000000000000000000001000000000000010000000000000000000001000000000000100000000000101001010011010101110100011011111001101111001000011011100011111101010000001001111010101010100101111010100110101011010010010101000111000001010010010011000010010000001000110100101010001101100001001100001000010010100100000000011101011101010001000100000000000000000100010010000001000000100000011001000101000000000000000000000010000000001000000000000000000000100000000100000000010000000000010000000000001000000100000001010000000000000000000100000000000000000000010000000000100000000000000000000???????????????????????????????????????????????????????????????????????????????????????????????????????????????????????????????????????????????????????????????????????????????????????????????????????????????????????????????????????????????????????????????????????????????????????????????????????????????????????????????????????????????????????????????????????????????????????????????????????????????????????????????????????????????????????????????????110101110100110101001111111000110010111001111111110100110100111000111000111101001000110011000110101000101001010000010001100101000000101001001010100111010010100000110001000100110010100001101001010011101000100100001100000100001000001001001000001000100001000010000001000000000000001000000000000100000100100000000000000000100000000000100010000010000000010000100000000100001100000101000000010000000000000000000000000000000010000000000001000000010000100000000000000000000000???????????????????????????????????????????????????????????????????????????????????????????????????????????????????????????????????????????????????????????????????????????????????????????????????????????????????????????????????????????????????????????????????????????????????????????????????????????????????????????????????????????????????????????????????????????????????????????????????????????????????????????????????????????????????????????????????????????????????????????????????????????????????????????????????????????????????????????????????????????????????????????????????????????????????????????????????????????????????????????????????????????????????????????????????????????????????????????????????????????????????????????????????????????????????????????????????????????????????????????????????????????????????????

aMeauOP010027 11011101110111010010110010000101101011010111010110110001110101101101101010001101001011000101011110101001101010101101101100100001101111001011010010010111010010011001000100110000110110000110100011111010001011100000100100111001000010010010000000110000100100100000000001000000011000000010010000010100011000100010000001010000000000001001000001010001001001001000100000000010000001000000001000011000010001000000010000000000000010000001000001000000100000000010000001000000100010000110100011000110101101101001011011010100100100010001001001000001000011010101110110111001010100111000010010010101010010010000101111000110100000001010001010100000010100010100001001001000100001011110000101011101111000010100000100100001011010000110000101010001000100000001100011000000001000010001000001001000010000000010100100000000000100000000100001000000000000000000000000100010000000000000110001001100010001000101010100001000101000000000010001000000000100000000000010000010000000000001000000000000001100000000010000010010010000000000000000001000011100000000000000100000000000010000000000000000000000000000000000000000000000000001000000000000000001000000000000000000000000000000000000100000000000000000000000000000000000001000000000000000000000000000000000000000000000000000000000000000000000000000000000001000010111001100101111101110111010000101010100101001000101110101100010100111001001001010100110100100100001011010001001000001010101101110010010001001010110000100000101101100111010111010010000101010001011110000111101001101001110000000101100001000000000010111000010001010100001010010110000100001000100000000000100000010000101000000100010000000000000000010000000000000100000000000100000010000001000000000001000000000000000000000001000000000000000000000000000000000000000000000000101000000000010011000100001011010010010101010001010010011010001101010100100100100101000100100100100101000001110010001000101000101000110100010000010001000101000001010000000001011001000100000010010011000001000000010000101010100000000110000010010010001000011011001001000010000001000000101000000000000000000000000010000000000000000000000000000000000000000000100000000100000000001000000000000001100000000000000000000100010000001000000010000??????????????????????????????????????????????????????????????????????????????????????????????????????????????????????????????????????????????????????????????????????????????????????????????????????????????????????????????????????????????????????????????????????????????????????????????????????????????????????????????????????????????????????????????????????????????????????????????????????????????????????????????????????????????????????????????????????????101110010101100111111010100110101011010110010101010010010110100010101101010101110000101010101010110001001011011110111000101011101001001111001001001001100000111000101000110100011000011000101000001101100010100101010000100000000000100101001000000010100101010000000000010010000000100101000000000000000000011000001000001000000000000000000000100000000000000000100000001010000000100000000000000000000000010000001000000000000000000000000000000100000000000011011010011010110111010101110010110101110101010110101101101011011101101100010001101110101111111010101000110010101011001110011011101010101101100101101010110101010100010101111110111111010000010110001000001101101010101101001001100001001000001010000101000000100011000000100000000100000000101001000100000110100000001010010000000001101110010000111000001001000010010000000000000000100000001000000001000000000000000100000000000000000000000100000000000000000000000000000000000100000010111011111011001100101111011010101011100101001001001001011010111101100101101001100011101000010010100110110100010100001010000110100101100110111101011000100100001010100011000010110101000010000011100001100101010010000100110111000000010010001000000000000110010010111001010000000000010000101000100010011010000110000000000100010000000000010000001000100000000100100000001001011010000000000000000000000000001000000000000000000000000???????????????????????????????????????????????????????????????????????????????????????????????????????????????????????????????????????????????????????????????????????????????????????????????????????????????????????????????????????????????????????????????????????????????????????????????????????????????????????????????????????????????????????????????????????????????????????????????????????????????????????????????????????????????1010111001001110101111011001010100100101001110111011011101010110001111011001101000101001110000101101001110101101010001100000101000000011000010011100010011100000101001110110100101111001000010100100000100000100000100001000000101100010000101000010000000010010000000000101100000000000000010000000000000001000000000000000000001000000000010000000100000000000010001000000010000000000000000000000000101101010100110010000110100100110101011000101011001100100011100010101010101011110000110110101010011011101101011111011010011000101000110000101010101000111100101100001100011100000000111010000010100111010010010011000000100100110000010001010110010100010000000000000000000000000000000100000000000100000000001000000000000011010100000000000000000000000000000001000010000000000000110000000000000000010000000000001100000000000100000000000001010001000000000000000000000000000000000000010000000010010010111110001010000010100111001100010101001010101011100110101010000100110111001001000101000010100010001010110101010001010110100101001100001000111001000100100101001000101001011010010000001000011000010000010000000100001000100100100000010000100000100000000001001000001000000011100010000000010100000000000000000000000001000000000000000100000001000000000000000000001000000000000010000000000000000000000000000000001000000000000100001000010010101100100101011011011101101001000111011110011101001011000101001111010101110100101111010100010101010010010100101000110100001100010010011000100010000001000100100101010000011000001001100001000010010100100000000011011011100110001000100000000000000000100010001000001000000100000011001000101000000001000000000000010000000001000000000000000000000110000000100000000010000000000010000000000001000000100000001010000000000000000000100000000000000000000010000000001000000000000000000000???????????????????????????????????????????????????????????????????????????????????????????????????????????????????????????????????????????????????????????????????????????????????????????????????????????????????????????????????????????????????????????????????????????????????????????????????????????????????????????????????????????????????????????????????????????????????????????????????????????????????????????????????????????????????????????????????110101111100110101001001011000111010011101111101100100110000110000110010111000000001000000000010101010100001010010100000000001001100001101001010100101010000010100100001000000100010101001100101010001001000100100011100000110010100010001101100101000100001000000000001000000000000101000000000001100000100100000100000010100100000000000100000000000000100001000000000000100000000000000000000000000100000100000010000000000000000000000000000000000000001100000010000001000000000???????????????????????????????????????????????????????????????????????????????????????????????????????????????????????????????????????????????????????????????????????????????????????????????????????????????????????????????????????????????????????????????????????????????????????????????????????????????????????????????????????????????????????????????????????????????????????????????????????????????????????????????????????????????????????????????????????????????????????????????????????????????????????????????????????????????????????????????????????????????????????????????????????????????????????????????????????????????????????????????????????????????????????????????????????????????????????????????????????????????????????????????????????????????????????????????????????????????????????????????????????????????????????

aMebaMZ011591 11011101110101010111011001000101101011001110010110101001100101101101101010001101001010101101011000001101101000101001011100100011001101001001010011010111000110001001000101100000110110000010100000000010000010000001000110100001000000010010000000000000101000100000001101000100010000000000001000000100001001100100000001000000000000001001000000000001001001001000000001000010000000000000101000010000010001000000010000010000000010000000000001000000000000000000000001000000000010010110100011100111001101101100011111010100110000110001001001000001010011010101110110011001100100101000010000110101010010010000101110101110100000001011001010100010010100110000100001001000100000111110000111010100111100010100100100100001011000001100000101010000000000100001100011000000000000010001000001001000010000000001100100000001000100000000000001000000000000000000010000000000000000000000110001001100010001000101110100000000101000010000010001010000000100000000000000000000000000010001000000010001001000000000000000000011000000000000000000000000010100000000000000100000010000010000000000000000000000000000000000000000000000000001000000000001000001000000000000000000000000000000000000000000000000000000000000000000000000001000001000000000000000000000000000000000000000000000000000000000000000000000000000000000010111000100101101101101111010101101010100101011000110110101110010010111001011001010101010000110100101011010000101011001001101011011001111011011010111001110101111011101011010110111011000100001011011101100100101001101000100001010100100001101001110000011001011011010101001010010100000010100000010100111000100100100010000100000110001010001000100000010000001001000010000001000000000110010101001000000100100000000010000000000000000000000000000001000100001000000000000000000000000000000000000011001000000101010010010001000001000101010010001101010101000100101010100100100100001000000000100010100000101000100000101000001010010001001001000000010010000000111001010100000011010000100001000100010000101010000001000110000010010010001010010000101001000000000000000010101000000000001100001000000000100001000000000001000100000000000000000000010000100100000000000100000010000000001000000000000000001000001000000100000010000??????????????????????????????????????????????????????????????????????????????????????????????????????????????????????????????????????????????????????????????????????????????????????????????????????????????????????????????????????????????????????????????????????????????????????????????????????????????????????????????????????????????????????????????????????????????????????????????????????????????????????????????????????????????????????????????????????????100110010101101011111010100100101011010110010101010010011010110111101101010101110001010010101100010001010011110111111000011010101101001110001001001001100000011000001001010000001000011001101000001101000110100011010000001000000000100101001000000010100101010001010000010010000110100101010000000100000000011000011000010000001000000000000000100001000000010000100000001000001000100000000000000000000000010000001000000010000000000000000100000100000100000011011010110011110111010101110111110101010101010100101101101011010101100101110011011111011111111010101010010101000010000100111001101010100100100100101000100111010100010100111100101011010001010110001000001101101010001101101001100001010000001101100100000010110011000000100000100100000000111001000100000110100000001010100000000001110101001000101000001001101000110001000000000100000000001000001001000000000010000100000000000000100000000000000000000100000000101000000000000100000000111001111011001000101111011011111111110111101111110101111010111101110111100001001111011010010011101111111001111111011110000111101011101110111101011101111111001010110101101010111011000011011010100001101110010000000110010010000000010010000000001000000110010110111011000001000000010000101000101100001000000001001000000000100100000000000000011000100000001101000000100000001100001000000000000000000000010000000000000000000000000???????????????????????????????????????????????????????????????????????????????????????????????????????????????????????????????????????????????????????????????????????????????????????????????????????????????????????????????????????????????????????????????????????????????????????????????????????????????????????????????????????????????????????????????????????????????????????????????????????????????????????????????????????????????1010111001101110101110011011010110111001100100110001011101010110001011001101001001101001000000100010001100101101000001000000101000000000000010010100011001100010101001110110100101100100000010100100000100000110000100001000000001100010010001000010000000000010010000000101101010000000000010001000000000000000000000000000000010000000000010000000000000010000010000000000010000000000000000000000000101010010100100011001010100100110101010000101011001000101011100011101000100011010000100111001100011010101100001010000000010100001000100000001000101000111000101101000000001000000000111010000000000111010010000010000000100000010001010010010110110010000000000000000000000000000000000100000000010100000000000000000000000011010000001000000000000000000010000001000010000000000100110000000000000000010000000000001000000010000000000000000001010001000000000000000000000000001000000000000000000010010010011000001010010010100011001011000101001010101001100010101010100100110110001010000101011010100010000100101001010100010110010110010100001100111001000100100111011000101001010000010000000000001000010000001001001000001000010100100000101000100000000100000000001000000100000011100010000001000000000000100000000000000001000000000001000100000001000000000000000000001000000000000010000000000000000000000000000000000100000000001100101000010100001100100001011010011000101001000111011010010101001010000101001101010101010100101111010100010010010010000010101010110000001010010010100000101001010001000100100101000000101000001001100001100011000100100000000010011010100010001000100000000000000000110010001000000100000100000011000000100000000000000000000000000000000001000000000000000000000100000000100000000000000000000000000000000001000000000000101000000000000000110000000000000001000000000010000100001000000000000000000000??????????????????????????????????????????????????????????????????????????????????????????????????????????????????????????????????????????????????????????????????????????????????????????????????????????????????????????????????????????????????????????????????????????????????????????????????????????????????????????????????????????????????????????????????????????????????????????????????????????????????????????????????????????????????????????????????????????????????????????????????????????????????????????????????????????????????????????????????????????????????????????????????????????????????????????????????????????????????????????????????????????????????????????????????????????????????????????????????????????????????????????????????????????????????????????????????????????????????????????????????????????????????????????????????????????????????????????????????????????????????????????????????????????????????????????????????????????????????????????????????????????????????????????????????????????????????????????????????????????????????????????????????????????????????????????????????????????????????????????????????????????????????????????????????????????????????????????????????????????????????????????????????????????????????????????????????????????????????????????????????????????????????????????????????????????????????????????????????????????????????????????????????????????????????????????????????????????????????????????????????????????????????????????????????????????????????????????????????????????????????????????????????????????????????????????????????????????????????????????????????????????????????????????????????????????????????????????????????????????????????????????????????????????????????????????????????????????????

aMebaMZ011592 01010001010101010010010000000101101011001110010110101001100101101010001010001100101010001101101000010101001000101000011100100001001101001001000000010001000110001001000101100000110110000010100000001010000010000001000100100001000000010100010000000000101000100001001001000100010000000000010000000100001000100010000001000000000000001001000000000001001001001000000001000010000000000000000000010000010001000000010000100000000010000000000001000000000000000000000001000000000010010110110010000111001101111101011111010100110000110001001001000001100011010100110110111001100100101000010001010001010010010000101110001110100000001010001010100010011000110000100001001000100001011100000111101100111010011100100100100001011010001100000101010000000010100010100010000000000000010001000001001000010000000001100100000001000100000000000001000000000000000000010000100000000000000000110001001100001001000101010100000000101000000000010001010000000100000000000010000100000100000001000000000000001000000000000000000011000000001000000000000000010100000000000000100000010000010000000000000000000000000000000000000000000000000001000000000000000001000000000000000000000000000000000000100000000000001000000000000000000000001000000000000000000000000000000000000000000000000000000000000000000000000000000000010000010111000010101110101010111010101111010101101101000100110101100010100100001011001010110111001101000101011010010001001001010101001111010010010001010110010110101101000100111010101010010000000010001011101000110101010101101000001000100000011100010100000110010010001010101001010000100011100000000010100100000000100010000001000000101001010001001000000010000010000000100010010000000000010000010000000000001000000000000000000000001000000000000000000001000010000000000000000000000000000000100000011100100001011010010010011000001010011011001001101010101000110110001000010010100001000000001100010100000101000100000101010001010010000101010010000010100000000011001010100000100110010100001000000010000101010010001000110001001000000000110010100011001000000000010000010101000000000010100001000000000100000000000000001000000000000000000000000100000000100000000000100000000000000010000000000000000000010001000000010000010000??????????????????????????????????????????????????????????????????????????????????????????????????????????????????????????????????????????????????????????????????????????????????????????????????????????????????????????????????????????????????????????????????????????????????????????????????????????????????????????????????????????????????????????????????????????????????????????????????????????????????????????????????????????????????????????????????????????101110010100101011110100100100001011010111010101010100001000100110101111010101100010010010101100011001000011011110111000001010101001001111001001001001100001010000101000110000011100011000101000001101000000100101010000101000000010100001001000000010100101001001010000110010000110100101010000000010000000101000001000001000001000000000000000100001000000000000100000001010001001000000000000000000000000010000001000000000000000000010000100000101000000000011010100010011110111010001100010110100010101010100101101101001010001001101010001011111101111011011101000010101001011001100111010101010100100100101101000010111010100010100111100101111010001010100101000001101101010001101101101100001010000001011000101000010100011000000100000000100000000111001000100000110100000001011000000000001101001001001100000001001000010100000000000000100000000101000000001000000000010000100000000000001000000000000000000000000000000101000000000001000000001011001111011011000101111011010101101100101001101001001011010111101010101100001100011101010110010010110110100011101011110000110100001100110111101011001101110001010010001000010111011010001010010100001001101010000000110010010000000010100000100010000010110000010011001100000000000010000101000101010001000000101000000000001100000000000000000011000100000001001000000000000001100001000000000000000000000000000000000000000000000000???????????????????????????????????????????????????????????????????????????????????????????????????????????????????????????????????????????????????????????????????????????????????????????????????????????????????????????????????????????????????????????????????????????????????????????????????????????????????????????????????????????????????????????????????????????????????????????????????????????????????????????????????????????????1010111001001110110010010001000100110001101100101001011101010110011011001101101001101101000000100010001100101101000011000000101001000000000011011110010001000000101001110110100101101000100000100010000100000110000100000000000001100011000001000010000000000010000000000101101010000000000010001000000000000000000000000000000010000000000010000000010000010000010000000000010000000000000000000000000101010010100110011000010100100110101010000110111101100101011100011101100110011011000110111001100011011111100001011000000011000001000110001001000101000100100101111000000001000000000111000000000000111010110000011000000101000010001010001010110110010000000000000000000000000000000000100000000010100000000000000000000000010001000001000000000000000000000000001000010000000000101010000000000000000010000000000001000000010000000000000000000010001000000000000000000000000001000000000000000000010101010111100101010110010100111001101010101001010101011010010101010100100110110101011010101001100100010001010110101010001010110010101010100001101010001000010100111001000101000110000010000000000001000010000001001000100001000100100100000101000100000000100000000001000000100000001000010000010100000000010000000000000000001000000000000000100000001000000000000000000001000000000000010100000000000000000001000000000000100000000000100101010010011001100100001011001001001101001000111011000010101001001000101001101010101110100101111010100010001010010010100101010110100001010010010101100100010010001000100100101000000101000001001100001000011000100100000000010011010100010001000100000000000000000110010001000000100000100000011000000101000000000000000000000000000001001000000000000000000000100000000100000000010000000000010000000000001000000000000010100000000000001000000000000000000000000000010001000001000000000000000000000??????????????????????????????????????????????????????????????????????????????????????????????????????????????????????????????????????????????????????????????????????????????????????????????????????????????????????????????????????????????????????????????????????????????????????????????????????????????????????????????????????????????????????????????????????????????????????????????????????????????????????????????????????????????????????????????????????????????????????????????????????????????????????????????????????????????????????????????????????????????????????????????????????????????????????????????????????????????????????????????????????????????????????????????????????????????????????????????????????????????????????????????????????????????????????????????????????????????????????????????????????????????????????????????????????????????????????????????????????????????????????????????????????????????????????????????????????????????????????????????????????????????????????????????????????????????????????????????????????????????????????????????????????????????????????????????????????????????????????????????????????????????????????????????????????????????????????????????????????????????????????????????????????????????????????????????????????????????????????????????????????????????????????????????????????????????????????????????????????????????????????????????????????????????????????????????????????????????????????????????????????????????????????????????????????????????????????????????????????????????????????????????????????????????????????????????????????????????????????????????????????????????????????????????????????????????????????????????????????????????????????????????????????????????????????????????????????????????

aMebaMZ011593 11011101110101010110011001001101101011001110010110101001100101100101101010001100101010101101001000001101101000101001011100100011001101001001001011011011000110001001010100111000110110000010100010010010000010000001000100100001000000010010010000000000101000100001001001001000010000000000010000000100011000100110000001000000000000001001000000000001001001001000000001000010000000000000100000010000010001000000010000100000000010000000000001000000000000000000000001000000000010010100010010000110001101101010011011010100100001010001001001000001010011010100110110010100100100101000010001010001010010010100101010100110100000001010001010010010011000110000100001001000100000011100000101101100011010001100000100100001011000010100000101010000000010100010100010000000000000010001000001001000000000000010100100000001000100000001000011000000000000000000010000100000000000000000010001001100010001000101110100000000101100010000010001010000000100000000000010000000100100000001000000010000001000000000000000000010000000000000000000000000010100000000000000100000010000010000000000000000000000000000000000000000000000000000000000000000000001000000000000000000000000000000000000000000000000000000000000000000000000001000000000000000000000000000000000000000000000000000000000000000000000000000000000000000010111000010101110101110101010101111010100101110000110110101110010100111001011001010110110101100100101011010010101101001010101001101010011010001010111001110101001000100011010101010011010000010001000110000100101101101001100000100100100010000000000000011010010011010100001010010100011011010000010100100000000100010000001000000100001010001000000000010000000010000000010010000000000010000001000000000001000000000000000000000000000000000000000000001000010000100000000000000000000000000000000011001000010011010010010101000001001010011001001101010101000100100011000100100100100010000000100011000000101000100000001001010010010000100111000000010000000000111000010100000001010001000001100000010000101000100001000110001000010010001010010000101001000000000000100011101000000000001000001000000010000000000011100001000100000000001000000000100000000100000000000100000000000000100000100000000000000100101000000100000010000??????????????????????????????????????????????????????????????????????????????????????????????????????????????????????????????????????????????????????????????????????????????????????????????????????????????????????????????????????????????????????????????????????????????????????????????????????????????????????????????????????????????????????????????????????????????????????????????????????????????????????????????????????????????????????????????????????????101101010111110011111010100100011011110110010001010110011010101110101101010101100000010010101010010101010011011110111000011000101000000110001001001001000001110000001000110000001000011001101000000101000010100001010000101000000010000001001000000010100101001000010000010010010110100001010000000100000000111000001000001000001000000000010100100000000000000000100000001010001010000000000010000000000000011000001000000000000000000000000000001000000100000011011100011011110111010101110110111101000101010100101101101011010010001101010001100100101011101010101000110001010011010101001001101011011100100101101000100010010100010100111100011011010000010100001000001101110010001101100101100000000000001011000101000010110000000000100000000100000000001001000100000100100000001000100000000001110000001001100000001001101010110000000101010000001000000000001001000000000010000100000000000001000000000000000000000000000000000000000000001000000010110111111011001100101110011010101100100101101101001001011010111110100101100001011011101010010010011110110100010100001010100110100001100110101101001001101110001010100001000010111011000001010010100001001101010000000110010110000000010100100000010000010100000010111001010001100000010000101000101010001000000001000000000101100100000000000000011000100000001001000000000000001010001000000000000000000000000001000000000000000000000???????????????????????????????????????????????????????????????????????????????????????????????????????????????????????????????????????????????????????????????????????????????????????????????????????????????????????????????????????????????????????????????????????????????????????????????????????????????????????????????????????????????????????????????????????????????????????????????????????????????????????????????????????????????1010111001100100101110011011010110110101001101011001011101010100001011101101001001101001000000100000001000110101000001000000101000000000000010011101010001100000101001110110000101100100000010100010000100000110000100001000000001100011000001100010000000000010000000000101100100000000000010001000000000000000000000000000000010000000000010000000000000010000010000000000010000000000000000000000000100100100010001000000100010001110101010001011001010000110000000001100000000001000100000010000100010000000000000010001001110011011110110000010000010011100100000010010100100000000000000110000000010010000000000000000100000101000001000001010010100000010000000000010000000000000001000010001001010000000000001000000100000000001000000000001110101001000000100001000000000000000000110000000000000010010000001010001000000001000100000000000010100000000000001001000000000010001000000100110010100010011011011100101010101010101011001010010101001010101011010010100001100100010110101001010101001010100010001010100101010101010110010101010100001010110101000010100111101000101001010000010000000000001000010000101001001000001100100100100000101000100000000100000000001000000100000011100010000010100000000001000000000000000001000000000000000100000001000000000000000000001000000000000010000000000000000000000000000000000100000000000100001010010100101100100001011011000001101000000011011110011101101010000000001111010101111000101111010100010000011010000000001000110000001100010010000100100000000000000100000001000000101000000000000001000010000100110000000000001000100010000000100000000000000000000000000000000100000100000011000000001000000000000000000000000000000001000000000000000000000000000000100000000000000000000000100000000001000000000000000100000000000000000000000000000000000000000010000000000000000000000000000000??????????????????????????????????????????????????????????????????????????????????????????????????????????????????????????????????????????????????????????????????????????????????????????????????????????????????????????????????????????????????????????????????????????????????????????????????????????????????????????????????????????????????????????????????????????????????????????????????????????????????????????????????????????????????????????????????????????????????????????????????????????????????????????????????????????????????????????????????????????????????????????????????????????????????????????????????????????????????????????????????????????????????????????????????????????????????????????????????????????????????????????????????????????????????????????????????????????????????????????????????????????????????????????????????????????????????????????????????????????????????????????????????????????????????????????????????????????????????????????????????????????????????????????????????????????????????????????????????????????????????????????????????????????????????????????????????????????????????????????????????????????????????????????????????????????????????????????????????????????????????????????????????????????????????????????????????????????????????????????????????????????????????????????????????????????????????????????????????????????????????????????????????????????????????????????????????????????????????????????????????????????????????????????????????????????????????????????????????????????????????????????????????????????????????????????????????????????????????????????????????????????????????????????????????????????????????????????????????????????????????????????????????????????????????????????????????????????????

aMebeMZ011603 11010001010101010010010101001101101011010110010110111001110101101101101010011001011111000111111100101001001110101001101101011011001101001001110000010110100111001101001101110011101110010110000000000001001010001000000100100001100000010010010000100000100000100001001001000000010000001010010000000100001000101010000001101000000000001001000010010001001001001000000000010110000001000000000100010000010011000000010000010000000010000000000001100000000000000000000000100000000010010111110011000111001101111001011011010100110111111001001001010000100011010100110110110101100100101000010001010001010001010000101111000110100000001011011010010000010100010100101101001000100000011110000101010100111110001110100100100011011010000100000101010000000100000011101010000000100101010001000001001000010010000010100100000000010100000000000001000000000000000000000000000000000000010000010001001101010101000101100100000000101000000000010001010000000100000000000010000000100000000001000000010000001000000100000000000010000000000000000000000000010101000000000000100000010000010000000000000000000000000000010000000000000000000001000000000010000000001000000000000000000000000000000000000000000000000000000000000000000000001000000000000000000000000000000000000000000000000000000000000000000000000000000000000000010101000010001111101111111010101111010101101101001101010001100010100110001011101010110110101101000001011011010001001001000101101111000011011011010110010100101101001100110010111010010010000010001010101000100101010101000000000000100110001001000000010011000010001001101010010000100000010000000010100100000010100010000000000000100000000000000000000010000010100000100000010000000010010000001000000000011000000000100000000000000000000001000000000000000000010000000000010000000000000000000010011001010001001010010010011000001010101011010001101010100000100101010100100100100100100100001110010010010001000101000101000010010010001001001000000010000000000011010010100000000010000100000100000010000101010100000000110001001000000001000010010101001000000000000000000101000000000000000001000100000000000100001000001000000000000001000000000100000000000000000100000000000000000010000000000000000000100001000000100000010000??????????????????????????????????????????????????????????????????????????????????????????????????????????????????????????????????????????????????????????????????????????????????????????????????????????????????????????????????????????????????????????????????????????????????????????????????????????????????????????????????????????????????????????????????????????????????????????????????????????????????????????????????????????????????????????????????????????101110010100101011111010100100001011010111001001011010010010100110101111010101100000101010100010000001001011011100111000011010101000001110001011001111100000011000001001010100011010011101001100001101000000100011110000000000000010000101001000000010100101010001000000010010100100100101010000000010000000011000001000000000010000000000000000100000000000000000100000001000000000000000000000000000000000010000001000000000000000000000000000000100000000000011011101010101110111010010110010110100010101010110101101101011010110101100010001010100101010101010101001110001000010010100001101101010100100100101101010100110010100010100111110101011010000100101001000001001101010001100101010100001100100001011000100011000110011000000100000100100000000101001000100001010100000001000101000000001110000001000101010001001000010010000000000000100000000010000001001000000000010001000001000000000100000001000000000000001000000000010000000001100000010110101111011001000101111011010101100100101101101001001011010111101000110100001000011101000010010100110110100010101001010000110100000110110101100101000101110001011010001000010110111000001010010100001000101000000000110010010000000000010000001000100010110010010011010100000100000010000101000101010011010000000100000000101000100000001000000001000100000000001000000000000101010000000000000000000000001000100000000000000000000000???????????????????????????????????????????????????????????????????????????????????????????????????????????????????????????????????????????????????????????????????????????????????????????????????????????????????????????????????????????????????????????????????????????????????????????????????????????????????????????????????????????????????????????????????????????????????????????????????????????????????????????????????????????????1010111001001111001110011011010110100101000100101001011101010110011111001101101001101001010000101001001100110101000011010000101001000001000010011100011001000000101001110100100011111000000011100010000100000010000100001000000101100000101101100010000000000010010000000101101000001000000010000000000000000000000000000000000010000000000010000000010000000000010000000000010000000000000000000000000101001010010001001010010100100101010101010001011110111000101010110001101011000100100010000010101000010001100000000000000000000000000000000000000000000000000000000000000000000000000000000000000000000000000000000000000000000000000000000010000001000000000000101000000000000000000000000000000000000000000000000000000000000000000000000000000100000000000000000000000000000000000000000000000000000010000000000000000000000000000000000000000000000000000000000000000000000000000000000000000000010011010111100010111010010100111001101100101001010101011110110100001000100110111011011010001010000100110001010110000010001001110000110010100001000111001100010100011001000101010010000001000000000001000010001010101010100001100010100110001010000100000000000000001001000000000000001000001000010000000000000000000000000000001000000000001000100000001000010001000000000001000000000000010000000000100000000001000000000000100000000001100001000010010101100100101001011011001101001010001001100010101110010100000001101000100101000101101010110010000110010010010000010110100001000110010110111100000000001100100000101001001011000011001010001011010000110100110000010011000100010000000100000000000100010010010010101000000000100100011001000001000000000000000101000010000010001000000000001000000000010100001001000100000000100001000000000100011100100000100000100000000000100000000000000000010000000000100000100000000000000000101100100???????????????????????????????????????????????????????????????????????????????????????????????????????????????????????????????????????????????????????????????????????????????????????????????????????????????????????????????????????????????????????????????????????????????????????????????????????????????????????????????????????????????????????????????????????????????????????????????????????????????????????????????????????????????????????????????????110101111010110101001100010000111010111001111110110000100000110000111010110000000000000000000011111000101111010100101000110100011101001101100010000101000100111011100000000000100001101001000001010111001000100101001000000110010100101011001000000000100001000000100101010000000000001001000001000100000000000000000100001000010000000000000000000000000000000000000000000000000000000000000000010000000000000000000000000000000000000000000000000000000000000000000000011000000001???????????????????????????????????????????????????????????????????????????????????????????????????????????????????????????????????????????????????????????????????????????????????????????????????????????????????????????????????????????????????????????????????????????????????????????????????????????????????????????????????????????????????????????????????????????????????????????????????????????????????????????????????????????????????????????????????????????????????????????????????????????????????????????????????????????????????????????????????????????????????????????????????????????????????????????????????????????????????????????????????????????????????????????????????????????????????????????????????????????????????????????????????????????????????????????????????????????????????????????????????????????????????????

aMebeMZ011604 110111011101010100110110010110111010110101110101101010011101101011011010100011010010110001011011001010010010101010010111001010111011010010011001010101011001110010110001001111001101000101100000110100100010100000000011001000010010000110000100001000001000001000010000010000000100000000100100000001000010001000100000010000000000000010010000000000010010010010000000001000100000010000000000000000000100110000000100000000000000100000000000010000001000000000000000001000000000100001101000110001110011011010010111110101001101001110010010010000001000110101001101101101011001101010000100010100010100010100001011100001101000000001110110101000000101001101001011010010001000001111100001010101001101000011100001000001010110100000000001010100000001001100011000100000001001000100010000000010000100000000011001000000000100000000000000010000000000000000000000000000000000000000010100110011000100010001011001100000001010000000000100010100001001000001000000100000101000000000011000000100000010000000000000000000100000100000000000000000000111010000000000001000000100000100100000000000000000000000000000000000000000000000010000000000000000000000010000000000000000000000000000000000000000000000000000000000000000000000000000000000000000000000000000000000000000000000000000000000000000000000000000000010000???????????????????????????????????????????????????????????????????????????????????????????????????????????????????????????????????????????????????????????????????????????????????????????????????????????????????????????????????????????????????????????????????????????????????????????????????????????????????????????????????????????????????????????????????????????????????????????????????????????????????????????????????????????????????????????????????????????????????????????????????10011001010001011010010010011000001010101011001001101010100000100110100100100100100101000100001100010010010001000100100101001010000010000101000000000010000000000111010010100000000001000100001000000010000100100100000000110001001000000001000010010101001000000000000000010101000001000000000001000100010000000000011010010000000000000000000000000100000000000000000000000000010000000000000010000000000000100001000000010000010000??????????????????????????????????????????????????????????????????????????????????????????????????????????????????????????????????????????????????????????????????????????????????????????????????????????????????????????????????????????????????????????????????????????????????????????????????????????????????????????????????????????????????????????????????????????????????????????????????????????????????????????????????????????????????????????????????????????101111010110101011101010100100101011010110101001010010010010100010101100010101100001001010100010010000101011011100111000001010101000001111001001001011000000011000001000110000011000011001101100001101000000010101010000001000001000100101001001000010100101010001000000010001100110000101000000000100000000001010001000001011010000000000000000100000000000000000000000001000000000100000100000000000100000011000000100000000000000000000000000000100000000000010011100010011110111010001100010110101010101010110101101101011011010101100010001101100101111101010101000110001010101010100001110101011000100100101101010100110010100010100111101101011100000100101001000001001101010001101101001100001100100001011000100011010100010000000100000000100000000101001000100001010100000001010101000000000110000010000100010001010000000000010000000010000000000010000000101000000000000101001001000000000100000001000000000101001000000000010000000001000000010111011111011011100101111011010001110100101001001001001101010111101100101101001100111001000010010010110110100010111001110100110010001110110101101001000100110000010100011000010110010001010010010100001100101000000000110010010000000010010000001000000000110010010010101001000000000010000101000101010011010000001000000000100000100000001000000001000100000001101000000000000001010000000000000000000000001001100000000000000000000000???????????????????????????????????????????????????????????????????????????????????????????????????????????????????????????????????????????????????????????????????????????????????????????????????????????????????????????????????????????????????????????????????????????????????????????????????????????????????????????????????????????????????????????????????????????????????????????????????????????????????????????????????????????????1010111001100110101111011011010110110101101110111001011101010110011111101101001010101101010000110001001100110101000011100000101101000001000010011100011001100000111001110100000001110100000010100101000100000010000100001100000001100000011101000010000000000010010000000101101000001000000000000000000000000000000000000000000010000000000010000000010000000000010000000000010000000000000000000000000101010010100100010000011010100110101011000101111001000100011000011101000100010010000001010001000011000001000000010000000000000000000000000100000001000000000101100000000001000000000110000000000000010010010000010000000100000000000000000010100000000000000000000000000000000000000000100000000000000000000000000000000000010000000000000000000000000000000000000000000000000000000100000000000000000000000000000000000000000000000000000000000000000000000000000000000000000000000000000000000000010010010111110001010000010100111001100010101101010101011100110101010100100110111010001010101010001100010011010100000010001001110010110010000001110110001000010000101001000101001010000010000001000001000010001011000000100001000010100010000001000100000100100000001001000000010000011000000000010000000000000000000000000000001000000000001000100000001000010000000000000001000000000000010000000000100000000001000000000000100000000001110001000010101001100101011011011011010101001000111011110010101100001100011001101000100100000101111010100010001010010000100000010110100011001110011011010100010000001001110001001010011001000000000000010000010000100101000000000001000100000001000100000000000100000000000001000000000000100000011000001000100000000000010100000000000000001001000000000010000000000000001100000000000000100000000000000010001000000000000000000000000000000000000000000000100000000000000000100000000000000000000000000???????????????????????????????????????????????????????????????????????????????????????????????????????????????????????????????????????????????????????????????????????????????????????????????????????????????????????????????????????????????????????????????????????????????????????????????????????????????????????????????????????????????????????????????????????????????????????????????????????????????????????????????????????????????????????????????????110101111100010100001001011010111000011001110110110100110100110011110111110101000001000000000110101110000010010100010000100001010000001001100011100101100000000100100001000010110001100011100001000001001000100101011100000100010000000000001000001010100001010010010001010001000000001001000100100100100000100000000000100000100000000000000010000000000000000100000000000010000000000001000000010000100000000000010000000000010000010000000000010000000000000000000000000000000100???????????????????????????????????????????????????????????????????????????????????????????????????????????????????????????????????????????????????????????????????????????????????????????????????????????????????????????????????????????????????????????????????????????????????????????????????????????????????????????????????????????????????????????????????????????????????????????????????????????????????????????????????????????????????????????????????????????????????????????????????????????????????????????????????????????????????????????????????????????????????????????????????????????????????????????????????????????????????????????????????????????????????????????????????????????????????????????????????????????????????????????????????????????????????????????????????????????????????????????????????????????????????????

aMebeMZ011605 11011101010101010111010001010101100011010111010110011001100101101101101010101001011010000101001000101001101010111000101100101011001101001001100101010111100110001001001100101000110110010110000010010011001010000001000100000001001000010000010000000000100000100001000001000000010000000010001000000100001000100010000001010000000000001001000000000001001001001000000000010010000000000000001000010000010001000000010000010000000000000000000001000000000000000000000000100000000010010111100011000111001101111101001011110100110111111011011101100000100011010100110110100101100110101000010001010101010001010000101110000110100000001011011010100000011000110100101101001000100000111110000101010100100110000100000100100101011000000100000101010000000100100001101010000000100101010001000001001000010000000010100100000000100100000000000001000000000000000000000000000000000000000000110011001100010001000101010100000000101000000000010001010000000100100000000010000000110000000001000000010000001000000000000000000011000000000000000000000000011101000000000000100000010000010010000000000000000000000000010000000000000000000001000000000000000001000000000000000000000000000000000000100000000000000000000000000000000000000000000000000000000000000000000000000000000000000000000000000000000000000000000000000000010110101010101110101110111010101101010100111011000101010101101010101111001011001010110110101100100111010010101001001001010101111111010010011011010110000100111101001101111010111010010100100010001111110100101101010101101100011000101100111010000000000011001010011010101010010110100101011000000010100100000110100100000001000000100010011001001000000010000010001000100010010000000001010100001000000000001001000000100000100000000000000000000000000000000000010000000000000000000001000000000010011001010001001010010010011000001010101011001001101010100000100101010101000100100100100000101110010010000101000000100101001001010010000101010000000011000000100111001010100000000010000100001000000010000101010100000000110001001000000000100010010101001000000000000000010101000000000000000001001000000000000000100000010000000000000001000000000100000000000000001000000000000000000000100100000000000000100000100000100000010000??????????????????????????????????????????????????????????????????????????????????????????????????????????????????????????????????????????????????????????????????????????????????????????????????????????????????????????????????????????????????????????????????????????????????????????????????????????????????????????????????????????????????????????????????????????????????????????????????????????????????????????????????????????????????????????????????????????110111010110101010111010110110001011010110000100110011010010100010000100000101100000101010100010000100000010011100001000001000101000010111001001001001000000010000000000110100001010011010101000001110000000000101010001010000000001000000001000000010100000001000000000000011100010100001010000000100000000011000001000000001010000000000000000000000000000000000000000001000000000100000000000000000000000010100000100000000000000000000000000000100000000000011011101010111110111010101110110110101010101010110101101101011010110101100010001011100101011101010101000110101010101001010001010101011000100100101101011000110010100010101011111011111100000010110001010001001101010011101101001100001100101001011000100011011010011000000100000000100000000101001000100001000100000001010101010000010110010001000101010001010000010010000000000010000000000010000001001000000000100101001001000000001100000001000000000101001000000000010000000001000000001110101111011010100101111011011101111100101101101001001011010111110101101100001000011101000010010010110110100011011011010100110101000110110101100101000100110001010000001000010110101000010010010100001000101000000000110000010000000010100000001010100010110010110010101000000100100010000101000101010001010001001000000000101000100000001000100001000100000000001000000000100001010000000000000000000000001000010000000000000000000000???????????????????????????????????????????????????????????????????????????????????????????????????????????????????????????????????????????????????????????????????????????????????????????????????????????????????????????????????????????????????????????????????????????????????????????????????????????????????????????????????????????????????????????????????????????????????????????????????????????????????????????????????????????????1010111001000111101110011011010110100101101111011001011101010100001011011101001001101101010000110001001100101101000011010000101101000001000010011100011001100000111001110100100010111000000011100101000110000010000100001100000101100000011001000010000000000010000000000101100100011000000010000000000000000000000000000000000010000000000010000000100000000000010000000000010000000000000000000000000101100010000011100100001000101110010000000100000000000000001100000000010000011000000000000000001000000000010000100100000010000001110110000000000011000100000100000000000000000000100000100000000000000000000000000000000000000000001000000000000100000010000001000000010000000000000000001000000000000000000001000000000000000110000000000000000010000000000000010000000000000000000011000000000000000100000000000010000000000000100000000000000000000000000000010010000000000010000000000000000000000010010111100100110100010100111001101010101101010101011110110101011000100110110010001010101011001100010010110110000111000001110000110010101001111010001100010100011001000101011010000011000001000001000010001001000010110001000010100110000010000100000100100000001001000000010000011000000000010000000000000000000000000000001000000000001000100000001000010001000000000001000000000000101100000000100000000001000000000000100000000001110001100010011001100101001011101011001101001000011011110010101010000110011000101100100101000101111011100010001010111010100100000010101001001110010110001100010000000011100001001100111011000010000000101010011000100100000010011011000100000001000100000000000000000000000000010000000010100000011000001101000000000000001000000000000000001000000000010000000000010000000100000000010000100001000000001010001000000000000000100000000000000100010000011110100000000000100001000010000000001010000100000???????????????????????????????????????????????????????????????????????????????????????????????????????????????????????????????????????????????????????????????????????????????????????????????????????????????????????????????????????????????????????????????????????????????????????????????????????????????????????????????????????????????????????????????????????????????????????????????????????????????????????????????????????????????????????????????????110101110100110101001100011000010010011010111011111001101001110000110000110000001010010010100010110000101001010000100100100100001100100110100001010010101001000100010000000010111000100001100011000001101101110100001100010100010101101000001100000000100101000100000001110000000001011000000000011100000101100000000000000000000001000001000000010011000000000000000000000000000000000011000101001000000000000000110110000000010100000000000000000000000000100000000000000000000100???????????????????????????????????????????????????????????????????????????????????????????????????????????????????????????????????????????????????????????????????????????????????????????????????????????????????????????????????????????????????????????????????????????????????????????????????????????????????????????????????????????????????????????????????????????????????????????????????????????????????????????????????????????????????????????????????????????????????????????????????????????????????????????????????????????????????????????????????????????????????????????????????????????????????????????????????????????????????????????????????????????????????????????????????????????????????????????????????????????????????????????????????????????????????????????????????????????????????????????????????????????????????????

aMelaurTWI01069 11011101010101101011010011010101101011101110110110111001110101111101101010101101011011101001101001001001111010101001011100101001001101001001100010010111100111001011001100111001111110010111100010001011001010010101000101111001000000111000010000001000101100100100100101000000010010001010010010000100001000110010000001010000010000001001000000010001001010001000000000000110000000000000000101100000010001000000010100100100000010000000000001000000000001000000000001000000000100010110110010000111001101101100000011010100100000010001001101000001000101010100010110100100000100101000010000000001010010000000101010001110100000000001000010000000010000110100000001000000000000011100000101010100000000000100000100000000011000000010000001010000000000000001100010000000000000010001000000000000000000000000100100000000000100000000000000000000000000000000000000000000000000000????????????????????????????????????????????????????????????????????????????????????????????????????????????????????????????????????????????????????????????????????????????????????????????????????????????????????????????????????????????????????????????????????????????????????????????????????????????????????????????????????????????????????????????????????????????????????????????????????????????????????????????????????????10110100010011101101110110010000101010100101010000100010001100010100101001011001010100010000000000001010000000000000000000001000011000010000000000110000100100001000000000100000010010000000000000000100000100000000101000000000000000000000000000000000010000010001000000010000000100000000001000000000100000000000000000000001000100000000000000000000010000000000000000000000000000000000000000010000000000000000000000000000000000000000000000000000000000000000000000000000000000000000000000000001000000000000000000000001000000000000010000000000000000000000000000000000000000000000000000000000000000000000000000000000000000000000000000000000010000000000000000000000000000000000000000000000000000000000000000000000000000000000000000000000000000000000000000000000100000000000000000000000000000000000000000000000000000000000000000000000000000000000000000000000000000000000000000000000000000000000000000000000000000000??????????????????????????????????????????????????????????????????????????????????????????????????????????????????????????????????????????????????????????????????????????????????????????????????????????????????????????????????????????????????????????????????????????????????????????????????????????????????????????????????????????????????????????????????????????????????????????????????????????????????????????????????????????????????????????????????????????100100010100100000101010100100001011000100010000010000010010100010000100000101100000010010100000000000000010011100000000011000100000000110000000000000000001010000000000010000000000011000000100000100000000000001010000000000000000000000001000000010100000000000000001000000000000000001000000000000000000001000001000000000000000000000000000000000000000000000000000001000000000000000000000000000000000010000001000000000000000000000000000001000000000000010001110010010110110010001100010110100000001010100100001101000010001000001010001101100000001101000001000000010000100000000000000100000000100100000101000000010010000010100000000001000100000000000000000000000100010001000000000000000000000000000000000000000000000000000000000000100000000100000000000000000000000000000000000000000000000000000000000000000000000000000000000000000000000000000000000000000000000000000000000000000000000000000000000000000000000000000000000000000000000011001111010000000000010001000000000000000001001010000000000010000000100000001000011001000000000000000010100000000000100000010000000100000000001011000100010000010000001000000000000000000000010000001000100000000000000000010000000000000000000000000000000000010001001000000000000000000000000000000000000000000000000000100000000000000000000000000000000000001000000000000001100000000000000000000000000000000000000000000000000000???????????????????????????????????????????????????????????????????????????????????????????????????????????????????????????????????????????????????????????????????????????????????????????????????????????????????????????????????????????????????????????????????????????????????????????????????????????????????????????????????????????????????????????????????????????????????????????????????????????????????????????????????????????????????????????????????????????????????????????????????????????????????????????????????????????????????????????????????????????????????????????????????????????????????????????????????????????????????????????????????????????????????????????????????????????????????????????????????????????????????????????????????????????????????????????????????????????????????????????????????????????????????????01101000000100010000001010100010101010000000011001000100011000000101000100010010000000010000000011000001000000000000000000000000000000000100000001000000000001000000000000000000000110000000000000000000010000000000000100000010000000000000110010000000000000000000000000000000000000000000000000000000000000000000000000010000000000000000000000000000010000000000000000000000000000000000000000000000000000000000000000000000000000000000000000000000000000000000000000000000000000000000000000000000010011101001010000010100011001000000101000010101001000010000000100000000110001001000000100000100010001010100000110000100110000100000000001000010000000000000001001000001000000000000000000000001000010000010000000000001000000100000000010000000000000000000000001000000000000001000000000000000000000000000000000000000001000000000000000100000001000000000000000000010000000000000010000000000000000000000000000000001000000000000101001000010000001010100001011010000000111000000001011000011101001010000000000101000101010100101111010100010000001010000000000000110000001000010010000000011000010000000100000001000000001000000000000000000010000100100000000000001000100000000000100000000000000000000000000000000000000100000011000000001000000000000000000000000000000001000000000000000000000000000000100000000000000000000000000000000001000000000000001000000000000000000000000000000000000000000000000000000000000000000000000000???????????????????????????????????????????????????????????????????????????????????????????????????????????????????????????????????????????????????????????????????????????????????????????????????????????????????????????????????????????????????????????????????????????????????????????????????????????????????????????????????????????????????????????????????????????????????????????????????????????????????????????????????????????????????????????????????1001011010000101000010000100000100000110101101011101010100001100001110001000000000000000000001101100000000000000000000000000000000000010010000000000010000000000001000000000001100001000010000010000010010010001000000000001000000000000010010000000001000010000000000010000000000000000000000000001000000001000000000000000000000000000100000000000000000000000000000000000000000000000000000000100000000000000000000000000000000000000000000000000000000000000000000000000000000000001001011000000101000000000000000110000000010010101011000000011000000000000000000100000000000000001000100001000000000000100000000001000000100000010000010000000010000000000000000000000100000000000000000010000000000000000000000000000000000000000000000000000000000000000000000000000000000000000000000000000000000000000000000000000000000000000000????????????????????????????????????????????????????????????????????????????????????????????????????????????????????????????????????????????????????????????????????????????????????????????????????????????????????????????????????????????????????????????????????????????????????????????????????????????????????????????????????????????????????????????????????????????????????????????????????????????????????????????????????????????????????????????????????????????????????????????????

aMelaurTWI01070 11010001010101010011010010010101100011101110010110011001110101111101101010101101011010100101101001001001111010101001101100101001001101001011100010010111000110101011001100111001110110010111100010001011101100001011100101111001000001001000100000001000101100101000100001000000010001001010010010000100001001100010000001010000010000001001000000010001001010001000000000000110000000000000001000100000010001000000010100101000000010000000000001000000000001000000000001000000000100010110100011000110001101101100000011010100100000010001001001000001000101010100010110010100000100101000010000000001010010000000101010000110100000000000000010000000010000110001000001000000000000010100000101000100001000000100000100000001011000000000000001010000000000000001100010000000000000010001000000001000000000000000100100000000000100000000000101000000000000000000000000000000000000000????????????????????????????????????????????????????????????????????????????????????????????????????????????????????????????????????????????????????????????????????????????????????????????????????????????????????????????????????????????????????????????????????????????????????????????????????????????????????????????????????????????????????????????????????????????????????????????????????????????????????????????????????????10110100010101111101110111010000110010101111101000100010000100010100101001011001010110010000000000001010010000000000000010001000000000010000000000110000100001001100100000100000010010000000000000000100000100000000101000010000000100000000000000000000011000010001000000010000000100001000000000000000100000000000000000000001000100000000000000000000010000000000000000000000000000000000000001000000000000000000000000000000000000000000000000000000000000000000000000000000000000000000000000000001000000000000000000000001000000000000010000000000000000000000000000000000000000100000000000000000000000000000000000000000000000000000000000000000010000000000000000000000000000000000000000000000000000000000000000000000000000000000000000000000000000000000000000000000000000000000000000000000000000000000000000000000000000000000000000000000000000000000000000000000000000000000000000000000000000000000000000000000000000000??????????????????????????????????????????????????????????????????????????????????????????????????????????????????????????????????????????????????????????????????????????????????????????????????????????????????????????????????????????????????????????????????????????????????????????????????????????????????????????????????????????????????????????????????????????????????????????????????????????????????????????????????????????????????????????????????????????100100010100100000110100100100001011000100010000010000010000100110001100000101100000010010100100000000000010010100001001101000100000000110000000000000000001010000000000010000000000010000001000000100000000000001010000000000000000000000001000000010100000000000000000000000000000000001000000000000000100001000001000000000000000000000000000000000000000000000000000001010000000000000000000000000000000010000000000000000000000000000000000000000000000000000000000000000000000100000000000000000000000000000000000010000000000000000000000000000000000001000000000000000001000000000000000000000000000000000000000000000000000000000000000000000000000000000000000000000000000000000010000000000000000000000000000000000000000000000000000000000000000000000000000000000000000000000000000000000000000000000000000000000000000000000000000000000000000000000000000000000000000000000000000000000000000000000000000000000000000000000000000000000000000011011111010000000000011001000000000000000001000110000000000010000000100000001000011101000000000000000010100000000000100000010000000100000000000111000100000000010000001000000000000000000000010000001000100000000000000000010000000000000000000000000000000000010001001000000000000000000000000000000000000000000000000000100000000000000000000000000000000000001000000000000001010000000000000000000000000010100000000000000000000000???????????????????????????????????????????????????????????????????????????????????????????????????????????????????????????????????????????????????????????????????????????????????????????????????????????????????????????????????????????????????????????????????????????????????????????????????????????????????????????????????????????????????????????????????????????????????????????????????????????????????????????????????????????????????????????????????????????????????????????????????????????????????????????????????????????????????????????????????????????????????????????????????????????????????????????????????????????????????????????????????????????????????????????????????????????????????????????????????????????????????????????????????????????????????????????????????????????????????????????????????????????????????????01101010100100010000011010100010101010000000111001000100001000000101000100010010000000010000000010000001000000000000000000000000000000000100000001000000000001000000000000000000000110000000000000000000000000000000000100000010000000000000100010000000000000000000000000000000000000000000000000000000000000000000000000010000000000000000000000000000000000000000000000000000000000000000000000000000000000000000000000000000000000000000000000000000000000000000000000000000000000000000000000000000011011100001010000010100011001000000101001010101001100010000000000000000110001001000000100000100010001110100000110000100110000100000000001000010000000000000001001000001000000000000000000000001000010000010000000000001000100100000000010000000000000000000000001000000000000001000000000000000000000000000000000000000001000000000000000100000001000000000000000000001000000000000010000000000000000000000000000000001000000000000101001000011000001010100011011010000000101000000001011100011101001010000000000101000101010100101111010100010000011010000000001000110000001000010010000000010000010000000110000001000000001000000000000000000010000100100000000000001000100000000000100000000000000000000000000000000000000100000011000000001000000000000000000000000000000001000000000000000000000000000000100000000000000000000000000000000001000000000000000010000000000000000000000000000000000000000010000000000000000000000000000000???????????????????????????????????????????????????????????????????????????????????????????????????????????????????????????????????????????????????????????????????????????????????????????????????????????????????????????????????????????????????????????????????????????????????????????????????????????????????????????????????????????????????????????????????????????????????????????????????????????????????????????????????????????????????????????????????1101011110001101000011000110000100000111101111011101010000001100001100001000000000000000000001101100000000000000000000000000000000000010010000000000010000000000001000000000001100001000010000010000011010010000000000000001000000000000000010000000001000010000000000010000000000000000000000000001000000001000000000000000000000000000100000000000000000000000000000000000000000000000000000000100000000000000000000000000000000000000000000000000000000000000000000000000000000000001001011000000101101010000000000110001001010010001011000000011000000000000000000100000000000000001000100001000000000000110000000001000000100000010000010000000010000000000000000000000100000000000000000010000000000000000000000000000000000000000000000000000000000000000000000000000000000000000000000000000000000000000000000000000000000000000000????????????????????????????????????????????????????????????????????????????????????????????????????????????????????????????????????????????????????????????????????????????????????????????????????????????????????????????????????????????????????????????????????????????????????????????????????????????????????????????????????????????????????????????????????????????????????????????????????????????????????????????????????????????????????????????????????????????????????????????????

aMezeDI011410 11010001010101010010010000010101100011001111010110101001110100101101101010001001001011001101011110101011111010101001011100101011001101001001100001011110100111101001001100110000110110000110100111010011001010010000000101100011001100011010010000100000101100110001010011010000010100010010010000000100001001111010000001000000000000100001010000010001001001001000100010000110000001000000000100100000010001000010100000100000000010100000000001000000100001000000000001000000000100010110110011100111001101111100001111011101110010111001011101010001010011010100110110111001010110111000010001010101011010010100101111100110100000011011111001100011010100110100101001001000010001011101000101110100111100111100100101101011011010000100101101010000000010000001101011000000000000100001010001000000010100000100110100000000000100000100001011000000001000000000000000100000000000000000110011001101010001100101111100000000101000011001111001010000110100100100000010000010100100000001000000010000001000000000010000000111000010000000000000001000011100000000001000100000010000010000000000100000000000000000010000000000000000000001000000000001000001001001000001000000000000000000000000010000100000001000000000000000000000001000000000000000000000000000000000000000000000000000000000000000000000000000000100001000010111000010101111101101101010100111010101111101000101010101100010100101001011001010111111100110100001011010001001001001000101001111001010011001011111010100101001111100000110001011011010101010000010101110100101101101001000111000101110101100000000000111010111001010110101010110100001010100000110100100000100110010000001000010101000010000000000001010000000010000101000010000000000010000001010000000001000001000010000000000001000000000000010000000000001000100000000010010000100000000100010001000100001011010010010011000001010001011010001101000101000100110010101000100100100100000001110010010000101101101000001010010010100001000000000000011000000000111000000100100001010010100000100000010000101000100001000110010000010000000110010010101001000000000000000000101000000001010100001000000000000000000000001010000000010000001000000000100010000000000000000000000000000000010001000000000000000101010000000100001010000??????????????????????????????????????????????????????????????????????????????????????????????????????????????????????????????????????????????????????????????????????????????????????????????????????????????????????????????????????????????????????????????????????????????????????????????????????????????????????????????????????????????????????????????????????????????????????????????????????????????????????????????????????????????????????????????????????????111101010100100011011011100101001011010110110111010010011010101110101101000101100011011010110100011000000010010110111001011101101100001111001001001001100001011000001001010100001000011000001000000111000000100101110000101000000000000001011000000010100101001000000000010010010010100101010000000010000000011000001000000000010000000000000101000000000000000000100000001000001000100000000100000000000000010000001000000000000000000000000000001000000000000010111010101011110111010110110110110101001011010110101101101011010101101000010001011110100111001001101010111011010010000100101001101010110100100101101100110110010100010100111100111111010000010101101000001011101010101101001100100001000000001111000100101001100011000010100000001100000001101001000100000110100000001000101010001011010110101000101000101001000000000101000000000100100101000000001001000000000000100100000100000000100000001000000010000000000000000000000000001000000001010101111011010100101110011010101100100101001101001001011010111110100101100001001011101000010010010010110101011101101010100110011001100110101101011001101100001010011101000010111011001001010010110011001101001000010110010101000000110010001100000100011110010010011001001000100001010000101000100010011010000110101000001101110100000000001001001010100000011001000000000101001010000000000000000000000000010100000000000000000000000???????????????????????????????????????????????????????????????????????????????????????????????????????????????????????????????????????????????????????????????????????????????????????????????????????????????????????????????????????????????????????????????????????????????????????????????????????????????????????????????????????????????????????????????????????????????????????????????????????????????????????????????????????????????1010111001100100101111011001000100101110011101011001011101010100011111001011101001101101110000110001001100100101010011010001101001000101000010011101010011010001101001110100100111110101000010100011000101000010000100001000000101101000111111000010000000100010000000000101101010011000000010000000000000000000100000000000000010000000000010000000000000001000010001000000010000000000000000000000000101101010100110011010011010110110101011000110111101100101011100010101000100011111000101111001100011001101101001111001010001000001010110101000000101100110100101101001000001000000000111000000010000011010110000010101000110010011000010011010110110000000000000100000000000000000000001100000000000100000000000000000000000010001000000000000000000000000000000001000011010100001101010000000000000000010010000000001000000010000000000010000010100001000000000000000000000000000000000000000000000010010010111100001010100010100111001101111101001010101011000110101010110100110111011011010101010110101010001010100001011001010110100111010100001100111001010011100011011000101010010000011000101000011001010001101101001100001000010100100000111100100001000100000101001000000101000011100010000010011000000001000000000001000001000000000000000101100101000001010000000000001000000000000010000000000000000000000000000000000100000000000100101000010011001100100101010101010000101001001011011000010101010000110011000101000101010100101111010100010000010110010010100100110010001000110010010011011001000000110110100101100001101000010101100001011011010100100100001010001010100110101000110010100010000000100010000101001000000100000011000100000010000000000001000000011000000001000001000001010000000000000010100010000010000010000001000000000001000000000100001010000000000100000000000000000000000000000001100100000000000000001000000000???????????????????????????????????????????????????????????????????????????????????????????????????????????????????????????????????????????????????????????????????????????????????????????????????????????????????????????????????????????????????????????????????????????????????????????????????????????????????????????????????????????????????????????????????????????????????????????????????????????????????????????????????????????????????????????????????110101111000111101011111011000110100111111111111010100110001111101111011111000001100001000100110101100010000010000100110000101000000101001000101110101010001000000100000100100110000100001100001001001001000100100011100101110000100000001001000000000100001000000000001010000000000000000000000000100000000100000000000000000000000000000000000000000000000000000000000000000000000000001000000010000000000000000000000000000010010000000000000000000000000000000000000000000000000???????????????????????????????????????????????????????????????????????????????????????????????????????????????????????????????????????????????????????????????????????????????????????????????????????????????????????????????????????????????????????????????????????????????????????????????????????????????????????????????????????????????????????????????????????????????????????????????????????????????????????????????????????????????????????????????????????????????????????????????????????????????????????????????????????????????????????????????????????????????????????????????????????????????????????????????????????????????????????????????????????????????????????????????????????????????????????????????????????????????????????????????????????????????????????????????????????????????????????????????????????????????????????

aMezeDI011411 11011101110101011011011011010101101011101111010110110001110101101101101010101110001010101101101100110011001010101001011101010011001101001011110001010101100111010011001100111000010100000110100111010010001010100001000110110001010100011010100000110000101000101001010001001000010100010100010000000100001000101010000001000001000001000001010000010010001010001000100010000110000001000000010100100000100010000010100000100000000010000000000001000000000001000000000001000000001000010110100011000110101101101010011111011101110111010001011101000001000011010100110110101001100100101000010001010101010010010100101110100110100000011011011010100001010100110001001001001000110010011111000101110100011000111101110100100011011010000110101101010000000010000001101011000000000000100001010001000000010000000010100100000000000100000000010011000000000100000001000001000000000000000100110011001100010001100101110100001001101000011000110001010000100100000100010010000100100100010001100000010001001000000000000000000011000010000000000000001000111100000000000000100000010000010000000000000000000000000000010000000000000000000001000000000001000001001000000001000000000000000000000000010000100000001000000000000000000001001000000000000000000000000000000000000000000000000000000000000000000000000000000000001000010101000010001111101001111010000110010101111110000101010101101011010111001011001010111111100100000011011010001001000001100101001011010010010011010110010110111101001100010110100010011000110010001010101000100001100101001101000000101000001101000000000111011010001010100001010110110001000000010100100111000110000010000001000000100010010001001000001010000000000000100000010000000010010000110100000000001000000000000000000000001100000000000010000000000001000000000000000000000000000000000000001000100000000010010010011000001000000010000001000000100000000100000001000100100100010000000100010010000101001000000001010010010010001001001000000010000000001011000000100010010010010100000100000010010001000101001000110010000010000001010010010101001000000000000001000101000000001010100000000110010000000000100001010000000010000001000000000100001000000001000000000000000000000100000000000000000001001010000001000000100000??????????????????????????????????????????????????????????????????????????????????????????????????????????????????????????????????????????????????????????????????????????????????????????????????????????????????????????????????????????????????????????????????????????????????????????????????????????????????????????????????????????????????????????????????????????????????????????????????????????????????????????????????????????????????????????????????????????110101010101101011111010100110101011010110110001010010010010100111101101011101101011010010101100011001000011011110111000101010101001011110001001001001100101011000001000110100011000011001101010001101001010100101010010100000000000100101001000010010100101000000000000010010010010110011001000000100000001011011001000000000100000000000000000100000000000000000100000001001001000001001000000000000000000010000001000000000000000000000000000001001000000000010011110010111110111010101110010111101001101010110101101101011010101001100010001101110101011001010101010110111010100010000001010101011010100100100101000100010010100010100111101011011100000010100001000001001101010001101100000100000000000001011000001001001100000000010100000001100000000101001000100000010100000001000101010000001011000001010000000101010000000001000000000000000000010000000001001000000000000001001000000000000000000000000000000000000000000000000000000000000000011010101111011011100101110011010101010101101001101010001101011010100000101101001011011101100010010010110111000011011001110000110100101100111001101011001100101001010110101000010110101010001010010100001001101001000010110010111000000010100001101000100011110010011011001010000000000010001001000100100011100000110101000001101110010000000010101001010100000001101000000000101001100000000000000000000000000100100000000000000000000000???????????????????????????????????????????????????????????????????????????????????????????????????????????????????????????????????????????????????????????????????????????????????????????????????????????????????????????????????????????????????????????????????????????????????????????????????????????????????????????????????????????????????????????????????????????????????????????????????????????????????????????????????????????????1011111001101110101111111011010110100111011111011001011101110100011011001101101010101001010000110001011100111101010010110001101001000101000010011101010011110001101001110100101111110001000010100101000101000110000100001000000001101010111001000010000000010010000000000101101000011000000010001000000000000000100000000000000010000000000010000000000000000000010010000000010000000000000000000000000101010010100100011010011010110110101010000110111101101101011100011101000100011010000100110001000011001101101001111010010001000101001110101000001001000110100101111001000001000000000111000000010000011010110000010101000100010010000010011010110010000010000000100000000000000000000001100000000000100000000001000000000000010001000001000100000000000000000000010000010000100000101000000000000000000010000000000001000000010000000000010000000100001000000000000000010000000000000000000000000000010010010111110010111010010100111011101010101001010111011000110101001000100110111011011010101001000101010001010100101110101010110100111010100001100111001010011100011011000101010010000011000101010011001010001101001010100001000010100100000111000100001100100000101001000000101000011100010000010011000000001000000000001000001000000000000000100100101000011010000000000010000000000000010000000000000000000000000000000001000000000000100001010010010001100100001011010011000101001000001011100010101010010000011101101010101010100101111010100010101010010010010101000110100001000010010010010011000000000100110000101000101011000001000100001010011010100101000000010011010100110001000100010100000000000100010001000101000000100000011001001000010000000000010000000000000000001000001000001000000000000000010100000000010000100000001000000000101000000000000001010000000000100000000000000000000000000000000000100001000000000000000000000???????????????????????????????????????????????????????????????????????????????????????????????????????????????????????????????????????????????????????????????????????????????????????????????????????????????????????????????????????????????????????????????????????????????????????????????????????????????????????????????????????????????????????????????????????????????????????????????????????????????????????????????????????????????????????????????????110101111100110101011011010000111000011001111101110100110000110001110001110010001000000000000010101000000100000000000001101101000010001001000000010101010010100000110100000001111000101001010001010001001000100100001000110110010101000000001100000000100001000010000011011011000000000011000100001100000100100000000000010000100000000011000010010000001000000000000000000100000000000001001000010000101000000001000000000001110001110000000100000000000000000000000000001000000001???????????????????????????????????????????????????????????????????????????????????????????????????????????????????????????????????????????????????????????????????????????????????????????????????????????????????????????????????????????????????????????????????????????????????????????????????????????????????????????????????????????????????????????????????????????????????????????????????????????????????????????????????????????????????????????????????????????????????????????????????????????????????????????????????????????????????????????????????????????????????????????????????????????????????????????????????????????????????????????????????????????????????????????????????????????????????????????????????????????????????????????????????????????????????????????????????????????????????????????????????????????????????????

aMezeDI011412 11010001010101010010010001000101101011001111010110110001100101101101101010001001001010000101101000101011001010101001111101010011001101001011000000101100100010010101001100111000110100000110100011010010001010100001000100100001101000010010010000100000101100111001010011001000010000010010010000000100001000101010000001001000000000000001010000010010001010001000100010000110000001000000000100100000100010000010100000100000000101000000000010000000100010000000000010000000001000010110100011000010001101101101011111111100110010110001001101000001010101010100110110101001100100101000010001010101010010010100101110100110000000011011001010100010110100110100101001001000110001011110000101010100111000011100100101100011011010000100101101010000000100000001101011000000000000100001010001000000010000000100100100000000000100000000010011000000000000000001000001000000000000000000010001101100011001000101110100001000101000000000010001110000000100000000000010000011100100000001000000000000001000000000000000000011000010000000000000000000011100000000000000100000010000010000000000000000000000000000000000000000000000000001000000000000000000000000000000000000000000000000000000000000000000000000000000000000000000001000000000000000000000000000000000000000000000000000000000000000000000000000000000000000010110100010001111101010111010100101010100101000000101110110101010100101001011001010110110110101000111011010001101010001100101010011011010010101010110010100101101001100010100101010100000100010001011101000101101010101001000001000111011001000000000000011000010001010111001010000100000010000000100100100000000000010000001100000101000010001101000000010000000010000100010010000000010010000110100000000001000000000000001000000001000000000000000000000000000000100000000000010000100000000100000001000100011010010010010011000001000000011010001101000100000100110010101000100100100000000001110010010000101001110000001000010010010001001001000000101000000000001000000100010000010011000000100000010010100000101000000110100000010000000100010010101001000000000000001000101000000001100100001000100010000000000100000100000100010000001000000000100010000000001000000010000000000000010000001000000000001000010000001000010100000??????????????????????????????????????????????????????????????????????????????????????????????????????????????????????????????????????????????????????????????????????????????????????????????????????????????????????????????????????????????????????????????????????????????????????????????????????????????????????????????????????????????????????????????????????????????????????????????????????????????????????????????????????????????????????????????????????????101110010101100011010010100100011011010100110101010010010010100110101101010101100011010010101100011001000010011100111001101010101001011110001101001001000100011000011000110000101000011001011010001101000010100011010000100000000000100101111000010010101101010000000000010010000011100111010000000100000001011000001000000000101000000000000101010000000000000000000000001000001001001000000010000000000000010000001000000000000000000000000000001000000000000011111101011011110111010101110111110110110101010100101101101011010101101000010001011110101011001011101000110011100010010101101001101011010100100101101101010110010100010100111100111111010000010101001000101001101010101101101100100001000000001011000100111001110011000010100000001100000010101001000100000010100000001000100010001101011001000110101010001001001010000101000000001010100101000000001011000000000000100100001000000001000000001000000010000000000000000000000000001000000110111011111011011000101111011010101011111101001101001001011010110101100100100001000011101000010010010110110101011111001010100110100101110110001101011000101110001010011101001010111011010001010010110011000101001000001010010110000000010100001000000100011110010010011001010000000000010000101000110010011100001010101000000100100000000000011000001010100000001101000000000100001100000000000000000000000000010100000000000000000000000???????????????????????????????????????????????????????????????????????????????????????????????????????????????????????????????????????????????????????????????????????????????????????????????????????????????????????????????????????????????????????????????????????????????????????????????????????????????????????????????????????????????????????????????????????????????????????????????????????????????????????????????????????????????1010111001100100101111011001010100110101001100100001011101010100011011001101001010101101010000110010001100100101010010110001101001000101000010011110010011110001101101110100101111111001000010100101000101000110000100001000000101101000101101000010000000010010000000000101101010010100000011000000000000000000100000000000000010000000000010000000010000000000010001000000010000000000000000000000000101101010010110011001011100100110101011000111111101100101011100010101100101011011000110110001100011101101101001111001010001000101001110100100001001010111000101111000000001000000000111000000100000011010110100011000000110010011000100011010110110000000000001000000000000000000000001100000000000100000000000000000000000010001000000000100000000000000000000010000011000100000101000000000000000000010000000000001000000010000000000000000010100001000000000000000100000000000000000000000000000010000010111100001010010010100111001100000101001010101011001010100101000100110111001011010101010101100010001010100001010000100110100111010100001001011001000010100011011100101010010000011000101001011001101001010001001000001000010100100001001100100100000100000101001000000100000011100010000100011000000000000000000000000001000000000000000101000101000011010000000000010000000000000010000000000000000000000000000000001000000000000101001100011010101100100001011010011001101001010011011100010101101001000010000101000101010100101111010100010100010010010010100100110100001001110010011010100010000000100110101001100010101000001001000011010011100100100100000011101010100101001000100010100010000000100010001000111000000100000011001000001010000000000001100000010000001001000000000001000000000000000100100000000010000100000000000000010001000000000100000100000000000100000000000100000100000000000000000100001000000000001000000000???????????????????????????????????????????????????????????????????????????????????????????????????????????????????????????????????????????????????????????????????????????????????????????????????????????????????????????????????????????????????????????????????????????????????????????????????????????????????????????????????????????????????????????????????????????????????????????????????????????????????????????????????????????????????????????????????100101110000110100001001010000110000011001111101110100110000110000110000100000000000000000000010101000000000000000000000000000000000001001000000000101000000000000100000000000100000100001000001000001000000000000000000000000000000000000001000000000100001100000000101000001000000100001000000000100000000100000000000000000000000110000000010000010100000000100000000000000000000000101000010010000000000001000000000000100010010000000100000000000000000000000000000100100000000???????????????????????????????????????????????????????????????????????????????????????????????????????????????????????????????????????????????????????????????????????????????????????????????????????????????????????????????????????????????????????????????????????????????????????????????????????????????????????????????????????????????????????????????????????????????????????????????????????????????????????????????????????????????????????????????????????????????????????????????????????????????????????????????????????????????????????????????????????????????????????????????????????????????????????????????????????????????????????????????????????????????????????????????????????????????????????????????????????????????????????????????????????????????????????????????????????????????????????????????????????????????????????

aMezeMU011548 00100001010101010010010010000101101011010110010100010001100101010101111010001011001010001001001000010001011010101001111100100011001101001001010001010101000110010001000100101000110100000110000010010010001011101001000100100001000011011000010000100000101100100000100001001000010000000000010000000100001000101010000011001000000000000001010000010001001001001000100000000010000001000000010000100000100001000010100000100000000010100000000001000000000000000000000001000000000100010110100011100111001101101100011011011100110011111001001101100001100011010101110110101001100100101000010101010001010010010100101111000110100000011011101010011010010100110000101001001000100000011111000101011100111000011100100100101011011010000100001101010000000100000001101011001000100000100001000001000100010000000010000100000000000100000000000001000000000000000000000000010000000000000100010001001100010001000101011100000000101100000100010001110000000100000000000010000011000100000001000000010000001000000000000000000011000010000000000000000000011100000000000000100000010000010000000000000000000010000000010000000000000000000001000000000010000001000000000000000000000000000000000000010000000000001000000000000000000000001000000000000000000000000000000000000000000000000000000000000000000000000000000000010000010111000010000101101001100010000100010100111000000100111101110010100101001011001010110110000110000101011100001101000001100101001111000010000001010111000100001101000100110100101010010101001010000000100000110001010101000000011000000100001101000000000011010010001000001000010011100001000000010011000100000010000010000001000000100000000000000000000010000000010000001000000000000010010000010010000000001000000000000001000000000000000000000010000000000100100000000000000000000100000000000010011100100001011010010010011000001010101011010001001000110000100110010001001000100100100001001100010010000101000010001001000010011010101001010010000011000000000011000010100000101010000100001000000010000101000100000000110001000010000000100010000101001000010000000000010101000000000100000001000100000000000000000000010000000000000000000000000100000000000000000001000000000000000100001000000000000000100010010001000000100000??????????????????????????????????????????????????????????????????????????????????????????????????????????????????????????????????????????????????????????????????????????????????????????????????????????????????????????????????????????????????????????????????????????????????????????????????????????????????????????????????????????????????????????????????????????????????????????????????????????????????????????????????????????????????????????????????????????100100010100100010111010100100101011010110010001010010010010100110101101010101100000010010110100010001000010011111011001101010101000000110001001001001100000110000100000110000001000011000100010001101000001100101010000100000000000100101001000000010101001010001010000010001000000110101000000000010000000011000001000001010001011010000000000100001000000000000100000001000001000100000100010000000000000011000000100000000000000000000000000001000000000000010011100010011110110010001100010110100000101010100101101101010010101001000010001101110101011101010101000110011000010100000001000101010100100100101101001010110010100010100111111011111010000010101001010001001101010001101001101100001000001001111000100010010110011000000100000000100000010101001000100000110100001001010100000001101110110001000101010101001000000000001000000101000000000000000001001000000000100001001000000000001000000001000000000000000000000110000000110001000000010111001111010011000101111011010101011000100101101010001011010111100100100101001000011101100010010010110110100011110001110001010100100110010011100011000100110001010100001000010110101001010010011110001000101000000011010010110000000110100001101000100000110010010010101100000100100010000101000100010011100001000100000000001110100000000001000001000100000011001000000000001101100000000000000000000000000000100100000000000000000000???????????????????????????????????????????????????????????????????????????????????????????????????????????????????????????????????????????????????????????????????????????????????????????????????????????????????????????????????????????????????????????????????????????????????????????????????????????????????????????????????????????????????????????????????????????????????????????????????????????????????????????????????????????????1010111001001101101110011011010100101101001101010011011101010100001011001101001010101101100000100010001100100101010010100000101001000111000011011100010011110001101001110100000011111011000110100100000100000110000100001100000001101010011101100010000000010010000000000101101010011000000010001000000000000000100000000001100010000000000100000000100000010001010001000000010000000000000000000000001001010010100110011000110100100110101011000110111101100100011100010101100101011011000110110001110011001101100101011000000001000101000110000101000101000111010101111000000011100000010111000000010000111010110000011001000100010010001100011010110111000010001000100000000000000000000001100100000000100000000001000000000000010001000100000100010000000000010000010100010000000000101010000000010000000010000000000000000000010000000000011000000100010000000000000000000000000001000000000000000000000010110111100001010000010100011001000000101001010101011010110100000000100110111001101000101010001100010101010110101010001100110100111010100001000010001000101100011001001101001011000011000101000011000010001010000010100001011010100100000101110100000000100000001001000000100000001000010000100001000000001000000000000000001000000000000000100000001000010000000000100010000000000000010100000000000000000001000000000001000000000000100001010110011001100100001011011011001101001000011011101010101101001000010001101000101010100101111010100011010010110010001101000010100001000110011000000100000000101100110000101000000101000000000000010000011010110100000000000001000100010101000100100100000000111010010010000000000000100000011000101000100000000000110100100000000000001000010000001000100000000000000100000000001001100000010000000000001000100000100010000000000000000000000000000000100001000000000000100000000000000001000000000???????????????????????????????????????????????????????????????????????????????????????????????????????????????????????????????????????????????????????????????????????????????????????????????????????????????????????????????????????????????????????????????????????????????????????????????????????????????????????????????????????????????????????????????????????????????????????????????????????????????????????????????????????????????????????????????????110101111100011101001011011010110010011010111101111100100000110000111000111111000000000001001011101100100100010000001100010000001000101001001000010101000000000100100101000100110000101001100001000011011010110100001000101110100100101010101101000000100001000000000001000000000000000000000000000110000000100010001000100000000000000000000000010000000000000100000000000010000000000000000001010000000000100000010000000000100000000000000000000000000000100000110000000000000000???????????????????????????????????????????????????????????????????????????????????????????????????????????????????????????????????????????????????????????????????????????????????????????????????????????????????????????????????????????????????????????????????????????????????????????????????????????????????????????????????????????????????????????????????????????????????????????????????????????????????????????????????????????????????????????????????????????????????????????????????????????????????????????????????????????????????????????????????????????????????????????????????????????????????????????????????????????????????????????????????????????????????????????????????????????????????????????????????????????????????????????????????????????????????????????????????????????????????????????????????????????????????????

aMezeMU011549 11011101010101010111011100000101101011110111010110010001110100101101101010001011001010101101011100010001111010101101111100100011001101001001100101010111000110010001000100111000110110000110000010010010001011101001100100100001000101010010010000100000100001000000110001001000010010000000010000000100001000101010000011010000000000000001010000010010001001001000100000100010000001000000000000100000100010000010100000100000000011000000000001000000000001000000000001000000000100010110100011100110001101101101011011011100110011111001011101000001100011010101110110100101010100111000010011010001010010011000101110100110100000011011001010100010010100110000101001001000100000011110000101011101011001110101100100100011011010000100001101010000000101000001100011001000100000010001000001000100010000000010100100000000000100000000000011000000000000000001000000100000000000000000010001001100011001000101110110000000101100000100000001010000000100000000000000000010000000000001000000000000001000000000000000000011000000000000000000000000011100000000000000100000010000010000000000000000000000000000000000000000000000000001000000000000000000000000000000000000000000000000000000000000000000000000000000000000000000001000000000000000000000000000000000000000000000000000000000000000000000000000000000000000010110101010010111101010111010100101010100101101000101010101101010100101001011001010110100100110000101011010001001000001100101001011010010000001010111001110101001000100110110101010011000100010001001100000100101010101001000010000100000011101000000000111010010001010101000010100100100001000000010100000000010000010000001000000100000010001000000000011000000000000100010010000000010010000010010000000001000000000000001000000000000000000000000010000000000000000000000010000000100000000000010011000100001011010010010011000001010101011011001101010101000100110010101010010100000100000000110010001000101010100001001010010011000000100110010000011000000000011000001100000000110000100001000000010000101000100101000110001000010000000100010000101001000010000000000010101000001000001000001000100000000000000000010010000100000000000000000000100000000000000100000100000000000000101000010000000000000100001010000100100010000??????????????????????????????????????????????????????????????????????????????????????????????????????????????????????????????????????????????????????????????????????????????????????????????????????????????????????????????????????????????????????????????????????????????????????????????????????????????????????????????????????????????????????????????????????????????????????????????????????????????????????????????????????????????????????????????????????????110110010110100010110010100100001011010110010001010010010010100010001101010101111000101010100100010001001011011110111100101010101000011110001001001000100000111000101000110100011000111000111111000101000110100011010000000000101000100101011000000111100101010001010000010000100000100101000000000100000000011000001000001001010111010000000000100001000000000000000000001000000000100001000000000000000000010000001000000000000000000000000000001000000000000011011100010111110111010010111010110101010101010110101101101011010101101101010001011100101011101011101000111011010010000100011010101010110100100101101000100110010100010100111110001011010000010110000010000101101010001101101101100001010001001111010101010010110011000000100000000100000010101001000100000100100000001000100000001101010100101000101010001001000000000001000100001000100000000000000101000000000010000101000000000001100000001000000001000000000000000000000000001000000010111001111011001000101110011010101010100100101001010001001010111101000101100001010011101100010010100110110101011011001111000110100100110010110100101000101110001010001001000010111011000001010011110001000101000000000110010010000000110010001101000100000110010010001001000000100100010000101000100010001100001010000000000101110000000001011000001000100000001001000000000100101000000000000000000000000000010100100000010000000000000???????????????????????????????????????????????????????????????????????????????????????????????????????????????????????????????????????????????????????????????????????????????????????????????????????????????????????????????????????????????????????????????????????????????????????????????????????????????????????????????????????????????????????????????????????????????????????????????????????????????????????????????????????????????1010111001101111001111011101101010110110101101011011011101010110001011011101010110101001100000100001001100100101110010110000101001000101000011011100011011110001101001110100011011100111000110100101000100000110000100001100000001101011011101000010000000010010000000000101101010011000000010000000000000000000100000000001100010000000000100000000010000010001010010000000010000000000000000000000000101010010100100011000011100110110101011000110111001000100011100010101100101010110000100110001100011011101100001110000010001000101000110000101000101100111010101101000000011100000010110000000010000111010010000010100000100000011001010011010110110000010000000000000000000000000000000100000000000100000000001000000000000010001000101000100000000000000010000001100010000000000101010000000001000000010000000000001000000010000000000011000000010010000000000000000000000000001000000000001000000010101010111010001011010010100111001101000101001010101011100110101010100101110110001101000101010100100010001010100001010001010110100101010100001101011001000010100111001001101001011000011000001000011001010001010000010100001011010100101000101110100000100100001001011000001001000001000110000010000100000001000000000000000001000000000000000100000001000010000000000100010000000000000010000000000100000000001000000000001000000000000100001010010010101100100101011010011001101001000001011000010101100001100011001101000101010100101111010110010101010010010100100000110100001010010011001010100011000001000100101001100001011010101010000101010011010100100000000001001010100000001100100000100010000101100011001000100010000100000011010001100010000000000010000000000000000001010001000001010000000000000100100000000010000101000010000000000001000000000001010000000000000101001111000000010100001000000000000100000000000000000000000000???????????????????????????????????????????????????????????????????????????????????????????????????????????????????????????????????????????????????????????????????????????????????????????????????????????????????????????????????????????????????????????????????????????????????????????????????????????????????????????????????????????????????????????????????????????????????????????????????????????????????????????????????????????????????????????????????110101111100110100001101011000111010011001110111110100110100110001111010110101000000101010000111101000101000010000000001100101010000101001101010110101000000001100010000000000110010101001100001010011101010100100001000010100010100101010101100001010100001100010000001010000000001001011010000000100000010100000001001001000100000000010000000010101000000000100000000000000000000000000000000010000010001010000000000000000010010000000001000000000000000000000010000001000000001???????????????????????????????????????????????????????????????????????????????????????????????????????????????????????????????????????????????????????????????????????????????????????????????????????????????????????????????????????????????????????????????????????????????????????????????????????????????????????????????????????????????????????????????????????????????????????????????????????????????????????????????????????????????????????????????????????????????????????????????????????????????????????????????????????????????????????????????????????????????????????????????????????????????????????????????????????????????????????????????????????????????????????????????????????????????????????????????????????????????????????????????????????????????????????????????????????????????????????????????????????????????????????

aMezeMU011550 11011101010101010010110100000101101101101110010110110001110100101101101010001011001010000101011100101001001010101001111101010011001101001001100101101101100110001001000100101000110110000110000010001010001011101001000101100001000000011010010000100000101010100000110001001000010000000000010000000100001000100010000011010000000000000001010000010001001001001000100000000010000001000000000100100000100010000010100000100000000010000000000001000000000001000000000001000000000100010110100011100111001101101101011111010100110010110001011101100001000011010100110110101001110100101000010011010101010010010100101110100111000000011011001010101010010100110000101001001000100000011101000101010100110000110100100100100101011000000100001101010000000100100001101011001000100000010001000001000100010000000010100100010000000100000000000001000000000000000001000001000000100000000000010011001100011001100101110100001000101000000000010001010000101100000000000010000011000100000001000000010000001000000000000000000011000010000000000000001000011101000000000000100000010000010000000000000000000000000000010000000000000000000001000000000001000001000000000001000000000000000000000000010000000000001000000000000000000000001000000000000000000000000000000000000000000000000000000000000000000000000000001000010000010110100010010100101000100010100101010100101100100101110101101010100101001011001010100110100100000111011010001001000001000101000011000010000001010111010100001001000100010100101010010010100010001111100000110001010101000000011000111100101100000000000011011011001010100010010010100000001001010010000100000000100010000001100000101000010001100000000010000100001000100000010000000010010000001010000000001000000000100000000000000000000000000010000000000000010000000000000000000100000000100010011001000001011010010010011000001010101011011001101010101000100101001100100100100100100000101110010001000101000100010001000010010100100101001010000011000000000011000000100000001010000101001000000010000101000100000100110001000010000000100010000101001000010000000000010101000000000000000101000100000000000000010000001000100010000000000000000100000000000000100000000000100000000100001000000000000000100000010000101000010000??????????????????????????????????????????????????????????????????????????????????????????????????????????????????????????????????????????????????????????????????????????????????????????????????????????????????????????????????????????????????????????????????????????????????????????????????????????????????????????????????????????????????????????????????????????????????????????????????????????????????????????????????????????????????????????????????????????101110011101110011111010100100001011010111010001010100010010100110101101010101101000010010100100101001001011011100111001101010101001001111001001001001010000111000101000110000011000011000101000001101000011010101010000100000000000100101001000011010100101010000010000010001100000100001010000000100000000001000001000001000001000010000000000010010001000000000100000001000001001000000100000000000000000010100001000000010000000000000000000001000000000000010010100010011110111010001100010110100000111010110101101101010010101001001010011101110101111101011101000111011010010100101001000101010100100100101101000100110010100010100111110111111010000010101001010001001101010001101101101100001101001001111110101010010010011000000101000000100000010101001000100000110100001001000100000001000110101101010100000101001000010000001000010101000100000000000001001000000000010001000000000000001100000001000000001001000000100101000000100001000100000111001111011011000101111011010101100100101001001010001001010110101000110101001000011001100010010010110111001011100001111001010100100110010101100101000100110000010001101000010111011001010010011110001000101000000000100010010000000110100001101000100000110010010010110100000100000010000101000100010011100001001100000000101100100000001011000001000100000011001000100000101000100000000000000000000000000000100100000001000000000000???????????????????????????????????????????????????????????????????????????????????????????????????????????????????????????????????????????????????????????????????????????????????????????????????????????????????????????????????????????????????????????????????????????????????????????????????????????????????????????????????????????????????????????????????????????????????????????????????????????????????????????????????????????????1010110101101101101111011011010110110111101111011001011111110110011011001101001110101101010000101010001100110101010011010000101001000111000011011100011011110001101001110100101011111011000110100101000100000110000100001000000001101011000101000010000001010010000000000101100100011000000010001000000000000000100000000000100010000000000100000000010000000000010010000000010000000000000000000000000100000000000000000000000000000000000000000000000000000000000000000000000000000000000000000000000000000000000000000000000000000000000000000000000000000000000000000000000000000000000000000000000000000000000000000000000000000000000000000000000000000000000000000000000000000000000000000000000000000000000000000000000000000000000000000000000000000000000000000000000000000000000000000000000000000000000000000000000000000000000000000000000000000000000000000000000000000000000000000000000000000010110111100001010010010100011001100000101001010101011000110101001000100110111001101000101010010100010101010100001010000010110010110010100001000010001001010100111011100111001011000111000001010001000110001010100001100011010010100100001111100100000000100000001011101001010000001100010000010000000000001000000000001000001000000000000000100000001000001000000100100010000000000000010000000000100000000000000000000001000000000000100001000010011001100110101011011011010101010101011011101010101101011110011001101000101110100101111010010010000010010010010101000010101001100110011010101101010100101000100100101000010101000001001101010010011010100101000001111001010100110001000100010100010000001100010001000100000000100000011001000000110000000000010000000000000000001000000000001000000100000000000100000000010001010000010000000000001100000000100010000000000010100101011001000000000011000000000000100000000000000001010000000???????????????????????????????????????????????????????????????????????????????????????????????????????????????????????????????????????????????????????????????????????????????????????????????????????????????????????????????????????????????????????????????????????????????????????????????????????????????????????????????????????????????????????????????????????????????????????????????????????????????????????????????????????????????????????????????????110101110100010101011001010000110000011001111101110100110000110010110000101100000001000001000110101001100000010000000000101001010000001001000010100101010000000100100000000000110000101101110101000001001000000100001000010100010110101010101101101100101111101010000101010000000000001000000000000110000000000000100000001110000000000000000010010000000000000110000001100000000000000000000000010000000000000000000000100000111010000000000000010000000000000000000000001000000001???????????????????????????????????????????????????????????????????????????????????????????????????????????????????????????????????????????????????????????????????????????????????????????????????????????????????????????????????????????????????????????????????????????????????????????????????????????????????????????????????????????????????????????????????????????????????????????????????????????????????????????????????????????????????????????????????????????????????????????????????????????????????????????????????????????????????????????????????????????????????????????????????????????????????????????????????????????????????????????????????????????????????????????????????????????????????????????????????????????????????????????????????????????????????????????????????????????????????????????????????????????????????????

aMezeMZ011614 11010001010101010010010100001101100011001110101110101001100101010010101010001001001010000101011100101001111100101101111101010011001101001001010001101101000110011001000100101000110110000110000010011010000100100001110100100001000000010010010000100000100100100000000001000100010000001000010010000100001001001010000001010000000000001001000000000001001001001000000010100010000001000000100100100000100001000000010000100000000010000000000001000000000000000000000001000000000100010111110011000110001101101100011011010100110101111001001001000100100011010100110110101001100100101000010001100101010010010100101110000110100000001011001010011010010100110001001001001000100000011011000101010100111000010100000100000101011010001000001100010101000100110001100011000000000000010001000001000100010000000010100100000001010100000000000011000000000000000000000001000000000000000000010001001110010001000101110110001000101100000000010001010000000110000000000010000010000100000001000000000000001000000000000000000011000000000000000010100000011101000000000000100000010000010000000000000000000000000000010000000000000000000001000000000010000001000000000000000000000000000000000000010000100000011000000000000000000000001000000000000000000000000000000000000000000000000000000000000000000000000000001000010000010110001000100110101100101010000110010100101100000100010001101010000100001010000010110100100000000011011100000010000001001101001110000010000001010110100100001001100100110100101010010000000010000001100000110001001101000111000000101100001000010100000010011010001000001000010000100000000101000000100100000000010010000001000000101000000000000000001010000000100000000000000000000000010000011010000000001000000000000000000000000000000000000000000000010000000000000000000000000000000000100000011000100001000100010010011000001000100010010001000000100000100100010001000000100100100001101100010001010001001010000001000010000010001001010000000010000001000011000000100000001010000000001000000010000100010100000000110001000010000000100010000101010000000000000000010101000000010001000000100000000000000000000011010000000000000001001000000100000000001000000000000000000000000000000000000000000000100010000001000000010000??????????????????????????????????????????????????????????????????????????????????????????????????????????????????????????????????????????????????????????????????????????????????????????????????????????????????????????????????????????????????????????????????????????????????????????????????????????????????????????????????????????????????????????????????????????????????????????????????????????????????????????????????????????????????????????????????????????101110010100110011111010100101001011010110011001010010011010100110001101010101100000010010101010010001010010011110101001101010101001001110001001001001000000011000001000010000001000011000101010001101010101000001010000001000000010110001001001000010100001011000010000010010100000100001010000000010000000001000001000000000000011010011000000100000000000000000100000001000000001000000000000000000000000010000001000000000000000000000000000001000000000000011011110011011110111010001100010111101001101010101101101101011011001101101010001111110101101101011101000111010000010000100011101101010101100100101101001000110010100010100111100111100010001010101001010000101101010001110100101100001001000001011000100000001110001000000101000000100000010101001000100010100100000001010100000000001110100001000010000001010101010110000000000000000100000010000001001000000000000101000000000000000100000001000000000001000000000000000000000001000000000111001111010011000101110011001001001000100101001010001011010010100110100100001000011010000010010010000111000010110001010100110101000110010010000111000100100001010010001001010111001001010000011000001000100000000000100000110000001010100001001000000010100010010010101000100100000000000001000101000001100000001001000000101010000000001001000001000000000001001000000000000101100000000000000000000000000000100000000000000000000000???????????????????????????????????????????????????????????????????????????????????????????????????????????????????????????????????????????????????????????????????????????????????????????????????????????????????????????????????????????????????????????????????????????????????????????????????????????????????????????????????????????????????????????????????????????????????????????????????????????????????????????????????????????????1010111001100100001010010001010100100101001101010001011101010100001011001101101101101001010000100010001100110101100000100000101000001111000010011100010001000010101001110100100101111010000010100100000101000110010100001001000001000010111101000010000000010111000000000101101000011000000000001000000000000000100000000000000010000000000100000010000000000000010010000000100000000000000000000000001001001001010101011000010101101010101000101000111010111010110001010110010001010011010010101010101101100001011001000101101011101100001010000000000000010000100010110011101100100010101100000000001000000000110000000000000001001010000010000000100000101000000101101001000000000000000000000000000000000000001000000000100000000000000000000000001000000000000000000000000000000000000010001010000000011000000000000000000000000000000000000000000000000010000000010000000000000000000000000000000000000000010011100001010100010110011001100000101001010101001000110001000001000110110001011000001000000100010001010100001010000100110000100010000001000010001000010000011001000101010010000010000000000001000010000010100000000001000000100100000010100101000000000100000001000000000000011000000000010000000000000000000000001000001000000000001000100000001000010000000001000010000000100000010000000000000000000000000000000001000000000000100101000010101001100100001011011011001101001000011011110010101000001000010000101010101010100101111010100010000010010110010001000010000001000010010000000100010010001000110100101000000101010011011000001000011000100100000010000001000100010001000100000100000000000100010001000100000000100000011000000100000000000000001000000000000000001000000000000000000000000000000100000000000000100000000000000000001000000000000001000000000000000000000000000000000000000000001000100000000000000000000000000???????????????????????????????????????????????????????????????????????????????????????????????????????????????????????????????????????????????????????????????????????????????????????????????????????????????????????????????????????????????????????????????????????????????????????????????????????????????????????????????????????????????????????????????????????????????????????????????????????????????????????????????????????????????????????????????????100001001100010000011001001000000000000001000000100000000000000010000000000000000001000000000000001000000000000000010000100000010000000000000100000000010000000010000000000000000000000000000000000001000000000000000000000000010000000000100000000000100000000000000000000000000000000000000000000000000000000000000000000000000000000000000000000000000000000000000000000000000000000000000000000000000000000000000000000000000000000000000000000000000000000000000000000000001111???????????????????????????????????????????????????????????????????????????????????????????????????????????????????????????????????????????????????????????????????????????????????????????????????????????????????????????????????????????????????????????????????????????????????????????????????????????????????????????????????????????????????????????????????????????????????????????????????????????????????????????????????????????????????????????????????????????????????????????????????????????????????????????????????????????????????????????????????????????????????????????????????????????????????????????????????????????????????????????????????????????????????????????????????????????????????????????????????????????????????????????????????????????????????????????????????????????????????????????????????????????????????????

aMezeMZ011615 11011101110101011010011101010101101011101110101110110001100101101101101010101101101011000101011100101001001010101001111101000011001101001001010001010101000110001001000100101000110110000010000010010010000100101001000100100001000000010010010000100000101100100000000001000100010000011010010010000100001001001010000001010000000000001001000001000001001001001000100001100010000001000000000100101000100010000010100000100000000010000000000001000000000001000000000001010000001000010111100010000110101101101100011011010100110110010001001001000101000011010100110110101001100100101000010101010101010010011000101110000110000001001011011010101010010000110000101001001000100001011101000101010100111000101100000101100101011010001000100100010010000100110001101011000000000000010001000001000000010000000010100100000001000100000000000011000000000000000001000001000100000000000100010001001100011001100101110100001010101100100001010001001100000110000000000010000010000100000001000000010000001000000000000000010011000000000000000000001000011100000000000000100000010000010010000000000000000000000000010000000010000000000001000000000001000001000000000000000000000000000000000000010000100000011000000000000000000000001000000000000000000000000000000000000000000000000000000000000000000000000000001001000000010110101010010110101010101010100101010100101010000101010101101010100101001011001010110110000101000001011010001001000001000101101011000010001001010110000100101101000100000100001010011001101011001010100010100101010101001000111000101100011101000110000010011010001010101010010011100011001001000010000101000000100010000001000000101000011011000000001010000000001000001010010000000010010000001010000000010000000000100001000000001000000000000000001000000010000100000000001000000110000000100010011000100010101010010010101000001010010011001101101010100000100100010101010100100100100000001100011000000001001100000001001010000010000101001000000010000000100011000000100010000110000100001000000010000100110100000000110001000010000000010010000101001000000000000000010101000000010000000001000100000000000000000011010000000000000001000000000100000000101000000000100000000000000000000000000000000000100001000000100000010000??????????????????????????????????????????????????????????????????????????????????????????????????????????????????????????????????????????????????????????????????????????????????????????????????????????????????????????????????????????????????????????????????????????????????????????????????????????????????????????????????????????????????????????????????????????????????????????????????????????????????????????????????????????????????????????????????????????100100011101110011000010100100001011000100001001010010010010100010101101010101100000010010100010011001010011011110111001101010101000000111001001001001100000010000001001010000011000011000100110001101001001010001010000101000000000000101001000000010101001011001010000010000100000100001010000000100000000011000001000001000000110001011000000100001001000000000100000001000000000100000100000000000000000010000001000000000000000000000000000001000000000001011111110011011110111010101110110110101001101010101101101101011010011101100110001010100101001011000101000010010001100000000000001100010101100100010101000100010010100010100111010111011010000000100001010110101100010011101101001100001010000001011000010000001101001000000101000000110000001101001000100010010011000001010101000000001110100001000100000001001101010010000000000001000100000010000001001000000000000101000001001000000100000001000000000101000000000000000000000001000000010110101111011011100101111011010101110100101001101010001101011010101000101100001000011101010010010010110111000010011001010100110100100100110001100101000100100001010011101001010110101000010011011100001000101000000001010010101000000010000001001000100010110010010010101010100100000010000101000101010001000001001001000000101110000000001010001001000100000001001000000000100000100000000000000000000000000000100000000000000000000000???????????????????????????????????????????????????????????????????????????????????????????????????????????????????????????????????????????????????????????????????????????????????????????????????????????????????????????????????????????????????????????????????????????????????????????????????????????????????????????????????????????????????????????????????????????????????????????????????????????????????????????????????????????????1010111101100100001111011011010110100101101101011001011101010110001111001101001110101001010000100010001100110101000011000000101000010111000010011100010001100010101001110100100101101010000010100100000101000110010100001001000001000000111001010010000000010111000000000101101010011000000010001000000000000000100000000000000010000000000010000000010000000000010010000000100000000000000000000000000110100000000000000001010000000000010000101000000010000010000000000000000000000000000000000000000000000000000000000000000000000000000000000000000000000000000000000000000000000000000000000000000000000000000000000000000000000000000000000000000000000000000000000000000000000000000000000000000000000000000000000000000000000000000000000000000000000000000000000000000000000000000000000000000000000000000000000000000000000000000000000000000000000000000000000000000000000000000000000000000000010011010111100001011010010101011001001000101001010101011100110101010100100110110101101010101100100100010001010100001011001010110010110010100001000010101000010100101001000101001011000010100010000001000010001001100000100001000010100100000100100101000100100000100001001001001000011000010000010000000000001010000000000000001000000000001000100100001000011000000000000010000000000000010100000000000000000000000001000001000000000000110001010010011001100100101011011011010101001001011011110010101101001100011001101000101010100101111010100010101010010110011000010110100001010010010100110100010000001000110101001100000101000001001000001010011010100100100011011011000100100000000100010100000000000100010001000000100000100000011001000000001000000000010100000000000000001000000000010000000000100000100100000000010001001000010000000000001000000000000001000000000000000000000000000000000000000000100000100010000000000000000000000???????????????????????????????????????????????????????????????????????????????????????????????????????????????????????????????????????????????????????????????????????????????????????????????????????????????????????????????????????????????????????????????????????????????????????????????????????????????????????????????????????????????????????????????????????????????????????????????????????????????????????????????????????????????????????????????????110101111100110101011101011010011010011001111111010100111100110101110111110111001111010101000111101110100011011000001110111001001001101001001110110101011000010101100100000001110000100001100001001001101010100110111000011100100100101001101101000010100011000000000011010001000011001101000010000100000100100000000001000000100000010010001010010000010000000100000000000010000000000001000000110010010000000000010000000000010010000000000100010000000000100000000000001000000001???????????????????????????????????????????????????????????????????????????????????????????????????????????????????????????????????????????????????????????????????????????????????????????????????????????????????????????????????????????????????????????????????????????????????????????????????????????????????????????????????????????????????????????????????????????????????????????????????????????????????????????????????????????????????????????????????????????????????????????????????????????????????????????????????????????????????????????????????????????????????????????????????????????????????????????????????????????????????????????????????????????????????????????????????????????????????????????????????????????????????????????????????????????????????????????????????????????????????????????????????????????????????????

aMezeMZ011618 11010001010101010011010100000101100011001111010100110001110100100100111010001001001010101001011010001001101011101001011100100011111101001001100101010111000110011001000100111000110100000110001010011010000010101001010101100001000000010010010000100000101100100000100001001000010000110010010010000100011000100010000001010000000000000001010001000010001010001000100001100010000001000000000100101000100001000010100000100000000010000000001001000000000000000000000001000000001000010110100010000110001101111100000011010100110000010001001001100101100011010100110110111001100100101000010001100001010010010100101110100110000001001011001010100010010100110001000001001000100001011110100101001100111000101101010100100011011010000100100101010000000100110001100011000000000000010001000001000000010000000010100100000001000100000000000011000000000000000000100001000000000000000000110001101110011001101101110110001000101100000000010001010000100100000001000010000010000100000001000000000000001000100000000000000011000000000000000000000000011100000000000000100000010000010010000000000000000000000000000000000010000000000001000000000010000001000000000000000000000000000000000000000000100000001000000000000000000000001000000000000000000000000000000000000000000000000000000000000000000000000000001000010000010111001010101111101110111010001111010100101101000101010101100010100101001011001010110110100101000011011011001001000001010101001011000010001001000110000100101101000100010100101010010000101010001001100000101001101101001101010000101100001101000110000011010010001010001010010000100001001001000010000100000000100010010001000000100000010001000000000010000000000000000010010000000010010000001010000000001000000000100000000000000000000000000000000000000000000000000000000000000010000000000000011010101010001010010010101000001010110010010101101010100000000100010000100100110110000000101100010100000001000110000000110010010010001001010000000010000000100011001000000010000000000000001000000010000100000100000000110000000000000000000010000101000000000000000001000100100000000000000000000000010000000001000000000000000000000000000000000100000000000000000000000000000000000000000000000000000000000000000000000000000000??????????????????????????????????????????????????????????????????????????????????????????????????????????????????????????????????????????????????????????????????????????????????????????????????????????????????????????????????????????????????????????????????????????????????????????????????????????????????????????????????????????????????????????????????????????????????????????????????????????????????????????????????????????????????????????????????????????100100010100101011111010100100001011010110110101011010011010110110101101010101100000010010100000010001010010011101010011011100101101001110001001001001100000111000001000110000011010011001001100001101000101010001010010001000001010000101001001000010100101011000010000010000100100100101001000000010000000001000001000001000000111010000000000100001000000000000000000011000000000100000100000000000100000010000001000000000000000000001000000001000000000101010111100010011110111010001101010110100000101010110101101101011010101001001010001010100101011101010101000111011010010000100011101101010101100100101101000100110010100010101011100111111010101010101001000101001101010001101101001100001001000001011000100000000100011000000101000000100000010100101000100011010100000001000100000000001010101001010101000101001101010100000000000001000100000000000001001000000000000100100001001000000100000001000000000101000000000000000000000001000000000110101111011011000101110011010101010100101001001001001011010110100010101100001000011101100010010100110110100010111001010100110101000100110001100101000100100000010001101001010110101001010010010100001000100001000000110110101000000010100001001000100010110010010010110100000100000010000101000101010001100000101001000000101100100000001001000001000100000001001000000000100101000000000000000000000000000000100000000000000000000000???????????????????????????????????????????????????????????????????????????????????????????????????????????????????????????????????????????????????????????????????????????????????????????????????????????????????????????????????????????????????????????????????????????????????????????????????????????????????????????????????????????????????????????????????????????????????????????????????????????????????????????????????????????????1010111001100110001111011001010110100111001101011001011101010110001111001101001010101001010000110010001000101001010011010000101000010111000010011100010001000001101001110100000101111010000011100100000101000110010100001001000001000010111101010010000000010010000000000101100100011000000010001000000000000000100000000000000010000000000010001000010000000000010010000000010000000000000000000000000100000000000000000000000000000000000000000000000000000000000000000000000000000000000000000000000000000000000000000000000000000000000000000000000000000000000000000000000000000000000000000000000000000000000000000000000000000000000000000000000000000000000000000000000000000000000000000000000000000000000000000000000000000000000000000000000000000000000000000000000000000000110100000000000000000000000000000000000000000000000000000000000000000000000000000000000000000000000000000000000000010010011011100001011010010100111001001010101001010101011000110101001000100110110011101010101101000100010001010100001010000010110110110010100001010010101000010100111011000101001011000011010001000011000010001001100001000001000010100100000101100101000100100000100001000101001000011000010000010000000000001010000000000000001000000000001000100100001000101000000000000010000000000000010100000000000000000000000001000001000000000000101001010010011001100100101011011010100101001001011011010010101101010000011001101000101010100101111010100010101010010010100000010110100001000010010100010100010100101001110001001000000011000100001000001010011010110100100001011001010100110101000110010100011000000100010001000010100000100000011001000011000000000000001000000010000001001000000000000000000000000000100100000000010001000000000010000000001000000000100001000000000000100000001000000000001100000000100000100000000000011100000000000???????????????????????????????????????????????????????????????????????????????????????????????????????????????????????????????????????????????????????????????????????????????????????????????????????????????????????????????????????????????????????????????????????????????????????????????????????????????????????????????????????????????????????????????????????????????????????????????????????????????????????????????????????????????????????????????????000101110100110100001101011010111010010101101110010010011100011001011001111000100100100001000001001010100100010010100101100101010000001001001000100101011000101001001000001001100001000010100010100110011011010000100000010001000100011000110010100001000010000000000000101000000000100000000000100110000000010000100000000000000000000110000000000000000000010100000000000001100000100001000100010000000000000000000100000000100000000000000000100000000000000000000000000100000000???????????????????????????????????????????????????????????????????????????????????????????????????????????????????????????????????????????????????????????????????????????????????????????????????????????????????????????????????????????????????????????????????????????????????????????????????????????????????????????????????????????????????????????????????????????????????????????????????????????????????????????????????????????????????????????????????????????????????????????????????????????????????????????????????????????????????????????????????????????????????????????????????????????????????????????????????????????????????????????????????????????????????????????????????????????????????????????????????????????????????????????????????????????????????????????????????????????????????????????????????????????????????????

aTrbbMZ011594 110110111101010110110101110110111110111011101011101010011101011111011110101011000110100001011010010011011110101010011011001010111011010011111001010101111001100100110011011010101101101101101001110100110110101100111001011110010001010110010110100010001011001010001011010011010101000010101100100001000111001000100100010000000000000000010101000100010010101010000000000000100000100000000000000100000100010000000100001000000000110000000000010000000000000000000010010000000000101101101100111001100111011111010010110101001100001100110111010000010101010101001101100110000001001010000100000000010100100100001010110001101000000000100000100000000100100100000000010000000000000111000001010011000111000011000001000000010110100000000001000100000001000000011000100000000000001000010000000010000000000000001001000000000001000000100000010000000000000000000000000000000000000001000111111011100110011011011101100011101010110101011110110110001101001011010100110101101101000010010100010101111010001000001100001011110001100000010010000100001111010100000010001000001100000100100100000000010001100001001000010000001000000000010000000000100000010011000000100000000000000000000001000100011000000110000000000000000100000010001000000000000000000000000000000000000001000000010000000000000000000000000010000100100101110000101011101011101110101101110101001010100001011101011110101001110010110010101100101011010000010110100101011010010101010011010010100110110101100101001010010101001110101010110100000000100010101010001111010101010011001010101001000000000000100001100000100010101010100100001000010110000000100001001001001000100100010000001010000100000010000000100000000010001000100100010000000100000010000000000010000000000000000000000000000001000000000000000000000000000000000000000000000000000000100110001000000110100100100110100010001010110010011010101010001001100100101001001001001000000011100101100001010000000001010000101100100010010010000000100000000000010000000000000000100001000000000000100011100001000010001100000000000000000000100001000001000000000000000001010000000000000000010000000000000000000000000100000000000000000000000001000000010010000000000000000000000000000000000000000000001000100000010000001000001111100011101100011011110110111010000100100101101000011001101010001111011101011011100101010100110110001111101011111011101000100000000001010101100010000010100000000011010010000001110101000100001001101001000010100010010010110000110100010000111000000000000000000010001010000001010000001000000000001001000000000000100010100000010000000000000111000000000000001000000000000000010000000000000000000000000000000000000000000000000000000000000000000000000000000000000010111101011110111111101110111010111111011011100111101001000111011110111101010111010001001010010010100000101001110101001010110110100100011110101000100101000001100000100001000011101001100011101001010100001001000101000000000000000000110100100000001010000000100000100000001000000110000110000101000000000001100000100000000100000000000000000001010000000000000100000000101001000000000000000000000000000001000000000000000000000000000000000000100000000000001011111010101111011101010111011011010100110101010010110110101101010110110001000110010010101100101010100011011100010101010000100110101100010010010010100010001101010001010101110010101110000010011000000000100110001000110010100010000100000000001100010000000010000100000010000000010000000010100100010000010000000000100000000000000011000010100010000000001000000000000001000010000000000000000000000100010000000000100000000000000000000000000000000000000000000000000000000000000000101011100111101101100010101001101010101111010110100100100101101011010010011110001101001101001000001000001010100001000000111000001010000010000000000010100010000000010011001000000011010000001001110100001100010000000000010001001000000001010000100000000000111000001001100101000000000001000010100010010000110000100100000000000110010000000000000000100010000000100100000000010100110000000000000000000000000000010000000000000000000000010111101110111110100100101001110111101111101011110111101001010011100011100011101011011001110101011100100011110111011110110110101010011010100010001001111111000100110101101111010100100100101011010010101010001010000000001011101001101101101000101000010000010000001100000010001000101010111001001011011100000000100010000110110000000000000001100000101010010000000100011010001000010001000100000000010000000000001001000001000010000000000000101011110100110000111001000101011011011100110011100101111101010000001000100110101000100101000010001000100010000100001001000010100000101000001101110101000100001011110111010000000110000000011010010110010001110000010000101000001100001011000100001000000000001000000010010110000000001000001000000001000000000001000010000000000000000100010010100000000000000001000000100010000000000000000000000000010110101011011101000101101010011010101100011111100100010101110101010110010110001100010011000101001100111100000001000001000100000000000000010000100100011100010011100000000100000000010000000000000011001011000001000000010100001100001001101011001000000000000010000000000000000000000010000000000010000000000000000000000001000000000000000000000000000001000000000001000000000000100000000000000000000000000000000100000000000000000000000001100000000000000000000000000000000000000000000000000000001001011110001011011001010011100110010010100101010111111011010000100010011011101000100000100000010001100101011010001001010011001011101010000100011100101000100001101100010101001000001000000000000100001100010100100100000100010010010000001000000000000010000010000100110101000000110000000001000000000001000000000000000000100000000000000010000000100000001000000000001000000000000010000000000000000000000000001100001000000000000010100101001001010110010101101110101110111100100011101111001110111010101001100111101010111010010111101110011011001011001010000000011011000110011101101100110001000000010010010101101000000100000100110000110001100010010000010001100100010011000100011000000001000000011001000000000010000010000001100100010100000001000000010000001000000000100000000000000000000011000000010000000100000000000001000000000000100000000000000010000000000000001001100000000000000000000010000001001000000000000000000000010111101011010111011010101010100011010010110001111101110100111101110000010000111011011100110001100111100100010101010110011110101110100111101110100010010000011010101100010000101011111110000001001100001011110001001010000001001101000000001010001001000010000001000100000001000000000000000000100000000000001000000010010000000010001000100000000000000000000000001000001000000001000000000000000000000000000001000000000000000100000100000000000000010011000000001101011011001101010110010110001110100110101110011001110101101100011110011110100010010000110001101101011010111000101101011010011001001010011010101101010100100000101101001101001100101100111001110110010011101001000010000101100001000000011010000000001001101000100000011000000000000010000000000001100000000000000000000000000000110000100000000100000000000010000000000100000000000000000000000100000000000000000000000000000100000000000000000000000000000000000000000000000000001001001011100011111101011100100100110111011011011100011110101111001000100011001010101001100010000001000101001000100100001111001110001001100100000010001010001010010100010000100100000000100000001000001000010000000000000000100100011010100000000000001000001000000100000000000001000000001000001000000000000000000000100000000000000000010000000000000110111001010010110011000001011000111010100011001100001000101001001100100000110000000110101100001100110011010100011010001000010000010000010001010000010001000000000000000100001000010001100000000000101000000100000100000000110000000010000000000000110000000000000000001000000000000000000000000000000100000000000000010000000000000000100000000000000000000010000000000000010000000000000000000000000000000100000000000000000000000000000000000000000000000000000010000000000000000000000000000
[truncated: 725,775 more chars]
